# Exploring the effectiveness of flavone derivatives for treating liver diseases: Utilizing DFT, molecular docking, and molecular dynamics techniques

**DOI:** 10.1016/j.mex.2023.102537

**Published:** 2023-12-29

**Authors:** Syeda Tasnim Quayum, Nusrat Jahan Ikbal Esha, Siam Siraji, Sanaa S. Al Abbad, Zainab H.A. Alsunaidi, Mansour H. Almatarneh, Shofiur Rahman, Abdullah N. Alodhayb, Khuloud A. Alibrahim, Sarkar M.A. Kawsar, Kabir M. Uddin

**Affiliations:** a Department of Biochemistry and Microbiology, North South University, Bashundhara, Dhaka 1217, Bangladesh; b Department of Chemistry, Imam Abdulrahman Bin Faisal University, Dammam 31441, Saudi Arabia; c Department of Chemistry, University of Jordan, Amman 11942, Jordan; d Biological and Environmental Sensing Research Unit, King Abdullah Institute for Nanotechnology, King Saud University, Riyadh 11451, Saudi Arabia; e Department of Chemistry, Princess Nora bint Abdulrahman University, College of Science, Riyadh, Al Riyadh, 11671, Saudi Arabia; f Lab of Carbohydrate and Nucleoside Chemistry, Department of Chemistry, University of Chittagong, Chittagong 4331, Bangladesh

**Keywords:** Flavone, Flavonoids DFT, ADMET, PASS, Molecular docking, MD simulation, Exploring the Effectiveness of Flavone Derivatives for Treating Liver Diseases: Utilizing DFT, Molecular Docking, and Molecular Dynamics Techniques

## Abstract

In exploring nature's potential in addressing liver-related conditions, this study investigates the therapeutic capabilities of flavonoids. Utilizing *in silico* methodologies, we focus on flavone and its analogs (**1**–**14**) to assess their therapeutic potential in treating liver diseases. Molecular change calculations using density functional theory (DFT) were conducted on these compounds, accompanied by an evaluation of each analog's physiochemical and biochemical properties. The study further assesses these flavonoids' binding effectiveness and locations through molecular docking studies against six target proteins associated with human cancer. Tropoflavin and taxifolin served as reference drugs. The structurally modified flavone analogs (**1**–**14**) displayed a broad range of binding affinities, ranging from -7.0 to -9.4 kcal mol⁻¹, surpassing the reference drugs. Notably, flavonoid (**7**) exhibited significantly higher binding affinities with proteins Nrf2 (PDB:1 × 2 J) and DCK (PDB:1 × 2 J) (-9.4 and -8.1 kcal mol⁻¹) compared to tropoflavin (-9.3 and -8.0 kcal mol⁻¹) and taxifolin (-9.4 and -7.1 kcal mol⁻¹), respectively. Molecular dynamics (MD) simulations revealed that the docked complexes had a root mean square deviation (RMSD) value ranging from 0.05 to 0.2 nm and a root mean square fluctuation (RMSF) value between 0.35 and 1.3 nm during perturbation. The study concludes that 5,7-dihydroxyflavone (**7**) shows substantial promise as a potential therapeutic agent for liver-related conditions. However, further validation through *in vitro* and *in vivo* studies is necessary.

Key insights from this study include:

• Screening of flavanols and their derivatives to determine pharmacological and bioactive properties using ADMET, molinspiration, and pass prediction analysis.
• Docking of shortlisted flavone derivatives with proteins having essential functions.
• Analysis of the best protein-flavonoid docked complexes using molecular dynamics simulation to determine the flavonoid's efficiency and stability within a system.

## Graphical abstract

**Figure:**
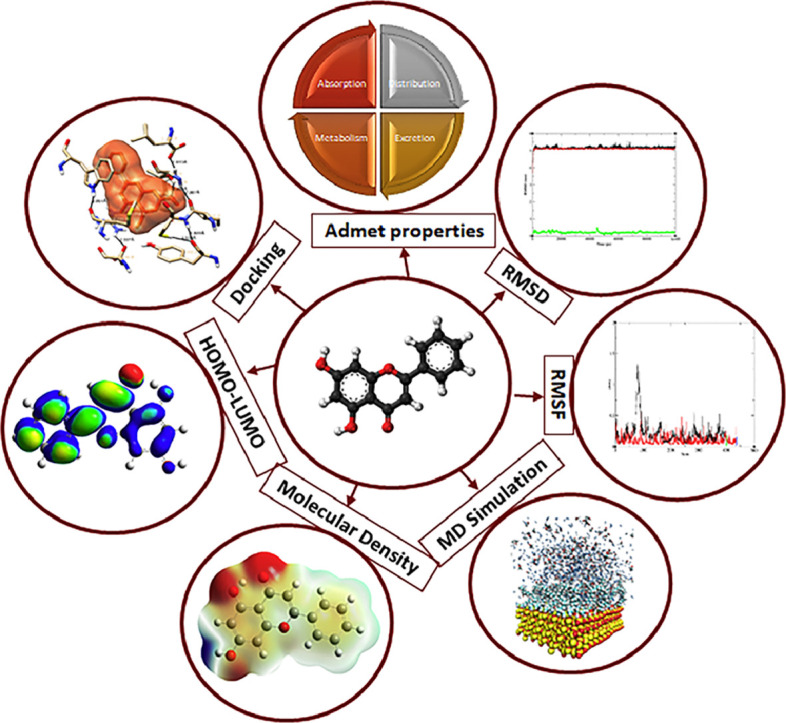

Specification table

**Table:** 

Subject Area:	Bioinformatics
More specific subject area:	Biomolecular simulations, molecular docking, liver disease, flavonoid orbitals, physiochemical properties of flavonoids.
Method name:	Exploring the Effectiveness of Flavone Derivatives for Treating Liver Diseases: Utilizing DFT, Molecular Docking, and Molecular Dynamics Techniques
Name and reference of the original method:	N.A
Resoruce availability:	N.A

## Method details

### Background

Liver disease is a foremost cause of mortality worldwide, affecting approximately 100 million individuals in the USA alone. The American Liver Foundation states that the development of liver fibrosis and cirrhosis can be attributed to the damage, inflammation, and subsequent regeneration of liver parenchymal cells [1], [2], [3]. Despite extensive research on liver drugs, not all medications can effectively treat individual levels and complications of liver disease [4]. Recent studies have shown that flavonoids, which are highly pharmacologically active compounds, possess antioxidant, anti-inflammatory, and antitumor properties and may be particularly beneficial in treating various liver diseases, including nonalcoholic fatty liver disease [5], [6], [7], [8]. Additionally, flavonoids exhibit anticarcinogenic, antiviral, and immune stimulation properties [7,[8], [9], [10]. In a recent *in vivo* study, Xi Le and his team found that the flavonoid quercetin can improve liver inflammation and fibrosis by activating and polarizing hepatic macrophages in BALB/c mice [11].

**Fig. 1:**
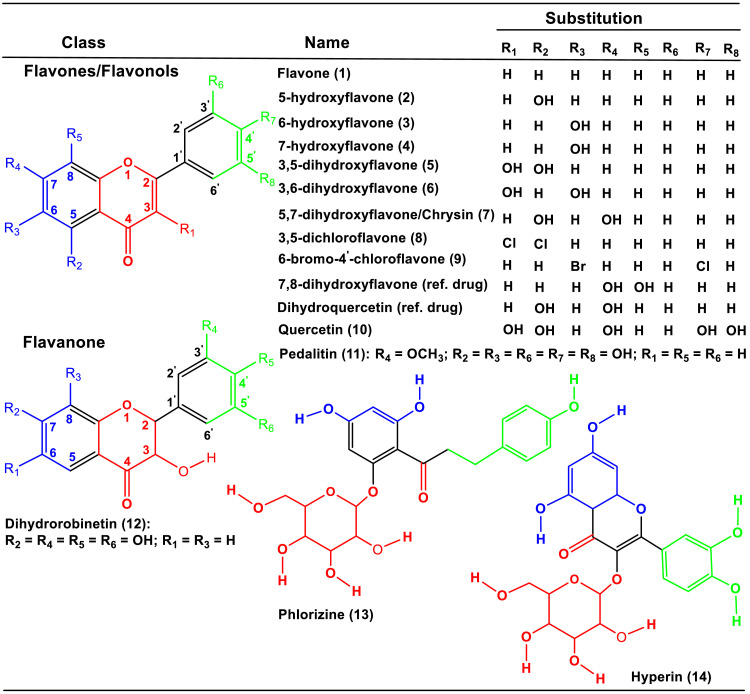
The chemical structures of flavone and its derivatives **(1-14)**.

Flavonoids, a group of secondary metabolites primarily found in plants, fruits, and vegetables, have gained attention in medicine and cancer therapeutics due to their potential benefits, including managing various health complications such as cardiovascular diseases, diabetes, cancer, damaged cell apoptosis, antioxidant activity, and infections caused by viruses and bacteria [12], [13], [14]^.^ These compounds exist in over 10,000 different classes and combinations, and they can be divided into eight groups: flavonols, flavanones, flavones, isoflavones, catechins, anthocyanidins, dihydroflavonols, and chalcones [15,16]. The molecular structure of flavonoids consists of carbon atoms arranged in two aromatic rings connected by a three-carbon molecule to form a diphenyl-propane structure. The flavonoid derivatives (**1**–**14**) are illustrated in Fig. 1.

Flavonoids have various biological activities due to multiple hydroxyl groups, sugars, oxygen, or methyl groups attached to their core structure [14], [15], [16], [17]. Flavonoids such as apigenin, rutin, and quercetin can suppress the inflammatory pathway and have chemopreventive effects against hepatotoxicity 11, 14–15. Preclinical studies have shown that flavonoids can protect the liver and mitigate liver diseases such as cirrhosis and nonalcoholic fatty liver disease [16,17]. Flavones are a fascinating group of compounds due to their chemical structure and numerous variations [18,19]. They are composed of a diphenyl propane structure with two aromatic rings linked to a pyran ring, and both rings can form a third ring as three carbon atoms connect them [20,21]. The presence of methoxy, ethoxy, propoxy, butoxy, or benzoxy groups on their aromatic rings leads to different pharmacological and downstream biochemical effects in the human body [22], [23], [24], [25]. Flavones represent the best class of flavonoids that exhibit various cellular and biological reactions in humans 22. The backbone of flavones can be modified with hundreds of variations, including hydroxylation, O—C-glycosylation, O-methylation, and acylation. Some glycosylated derivatives of flavones have antifungal effects, while the hydroxylation of C_5_, C_7_, C_3′_, and C_4′_ in flavonoids can increase bacterial inhibition by flavonoids [26], [27], [28].

Studies on flavones have revealed interesting findings on their biological effects. *In vivo* research conducted by Zhang demonstrated that flavones with hydroxy substitutions at positions 5, 7, 3′, and 4′ have effective antifibrotic properties as they bind to ALK5, which downregulates TGF-β/Smad signaling pathways [29]. An *in vitro*-*in vivo* evaluation of the flavone apigenin by Alexander Ghitu et al. demonstrated its antiproliferative and antiangiogenic effects against the SK-MEL-24 human melanoma cell line, thus reducing melanoma progression. Furthermore, Carla Sá and her team investigated the effect of Luteolin-7-glucoside (L7G) and found that it promotes liver lipolysis through induction of PPAR-α and CPT-represent-docked decreases HMGCR expression, which contributes to lower total and LDL cholesterol levels. Their study suggested that L7G-rich diets may be useful in controlling both NAFLD and CVD [26,[28], [29], [30]. Jian Lu et al. analyzed the effects of hydroxyl substitutions at positions 4, 5, and 7 in apigenin. They found that the substitutions inhibit the Akt signaling pathway and tumor cell proliferation by forming hydrogen bonds with the ligand molecules [28,29].

With the emergence of SARS-CoV-2, flavones have gained attention as potential therapeutic agents. Several studies have demonstrated that flavones have higher binding affinity and lower binding energy than current drugs such as hydroxychloroquine [29]. Several experimental studies have examined flavonoids and their derivatives, but in silico studies have been relatively limited [30]. However, recent research has focused on utilizing in silico analysis to evaluate the therapeutic potential of flavonoids in cancer, inflammation, and cardiovascular diseases. While most of these studies were obtained from PubMed, few have examined flavone analogs or compared the docking affinity of dihydroxyl or trihydroxy analogs with the same protein [31]. Researchers currently prioritize using in silico analysis to evaluate medications for their potential in treating various diseases, including cancer, inflammation, and cardiovascular disease [31], [32], [33]. For instance, an in silico study has been used to design target-specific docking with Isovitexin best-dockedceptor amino acids, providing insights into flavonoid efficiency as a SARS-CoV-2 drug [34,35]. Recent studies have also explored flavonoids’ anticancer and antimalarial properties using in silico analysis. One study investigated the immune checkpoint programmed cell death 1 pathway and concluded that flavonoids have potent anticancer abilities 36. Furthermore, a study on 25 novel synthetic derivatives of chalcone and flavone hybrids with 1, 2, and 3 triazole linkages for antimalarial activity found that optimization improved flavone binding affinity, resulting in higher antimalarial effects and more potent inhibition [35], [36], [37].

In our research we investigated six major proteins, each selected based on specific criteria and their potential relevance to liver-related conditions. The EGFR protein (PDB: 4UV7) was identified as the first protein of interest, inspired by Berasain and Avila's study, which suggested a potential role of EGFR in liver disease, particularly in conditions involving liver cell death. The link between EGFR and liver disease is supported by findings indicating that EGFR signaling provides protection against hepatocellular apoptosis induced by Fas ligand during acute liver injury and complications [38]. Furthermore, the HER2 protein (PDB: 7XJH) emerged as another focal point due to its potential involvement in liver disease, specifically hepatocellular carcinoma (HCC), the most prevalent form of primary liver cancer. HER2 overexpression has been observed in HCC, suggesting its potential as a therapeutic target for this disease. Shi JH and her team's work on HCC highlighted the correlation between HER2 overexpression and tumor stage, with 82% of samples exhibiting this relationship [39].

In addition to EGFR and HER2, the FPPS protein (PDB: 1YQ7), a vital component of the mevalonate pathway crucial for cell growth, was included in our investigation. Alterations in FPPS could affect liver function or contribute to liver disease. Another enzyme, HPGDS (PDB: 1V40), responsible for producing PGD2, a mediator of inflammation, was considered due to its expression in the liver and potential involvement in inflammatory liver diseases, as indicated in the Human Protein Atlas. DCK (PDB: 1p60), an enzyme pivotal for activating nucleoside analogs in cancer and antiviral therapy, was also among our selected proteins. Its expression and activity may be altered in liver diseases, with cirrhosis, for instance, associated with lower DCK expression and sensitivity [40]. Lastly, the Keap1-Nrf2 signaling protein (PDB: 1 × 2 J) was chosen for its linkage to liver disease. This signaling pathway impacts various liver diseases, including hepatitis, cirrhosis, and liver cancer. Keap1-Nrf2 signaling controls genes related to detoxification, inflammation, and cell survival. Disruptions in this signaling pathway can lead to increased or decreased Nrf2 activity, with diverse impacts on liver disease development and outcome [41].

Our study aimed to investigate flavone pharmacology, toxicity profiles, and biological activities and its 13 derivatives (**1 – 14**). We employed density functional theory (DFT) to assess their stability and molecular properties, including thermodynamic stability, HOMO-LUMO gap, hardness, softness, chemical potential, electrophilicity index, and dipole moment. We also analyzed molecular docking to evaluate their binding affinities against six targeted proteins using 7,8-dihydroxyflavone and dihydroquercetin as the reference drugs. We assessed each flavone derivative to predict their activity spectra for substances (PASS). We investigated how these analogs interact with various key proteins in the body using molecular docking. We performed molecular simulations to validate our findings and assess the entropic ability of the drug candidates. Furthermore, we carried out an MD simulation analysis on the active site of the protein‒ligand interaction to evaluate the stability of the protein‒ligand complex. Our study aimed to develop a novel class of targeted anti-liver disease drugs with significant biological action.

The flowchart of the study is shown below:

**Figure:**
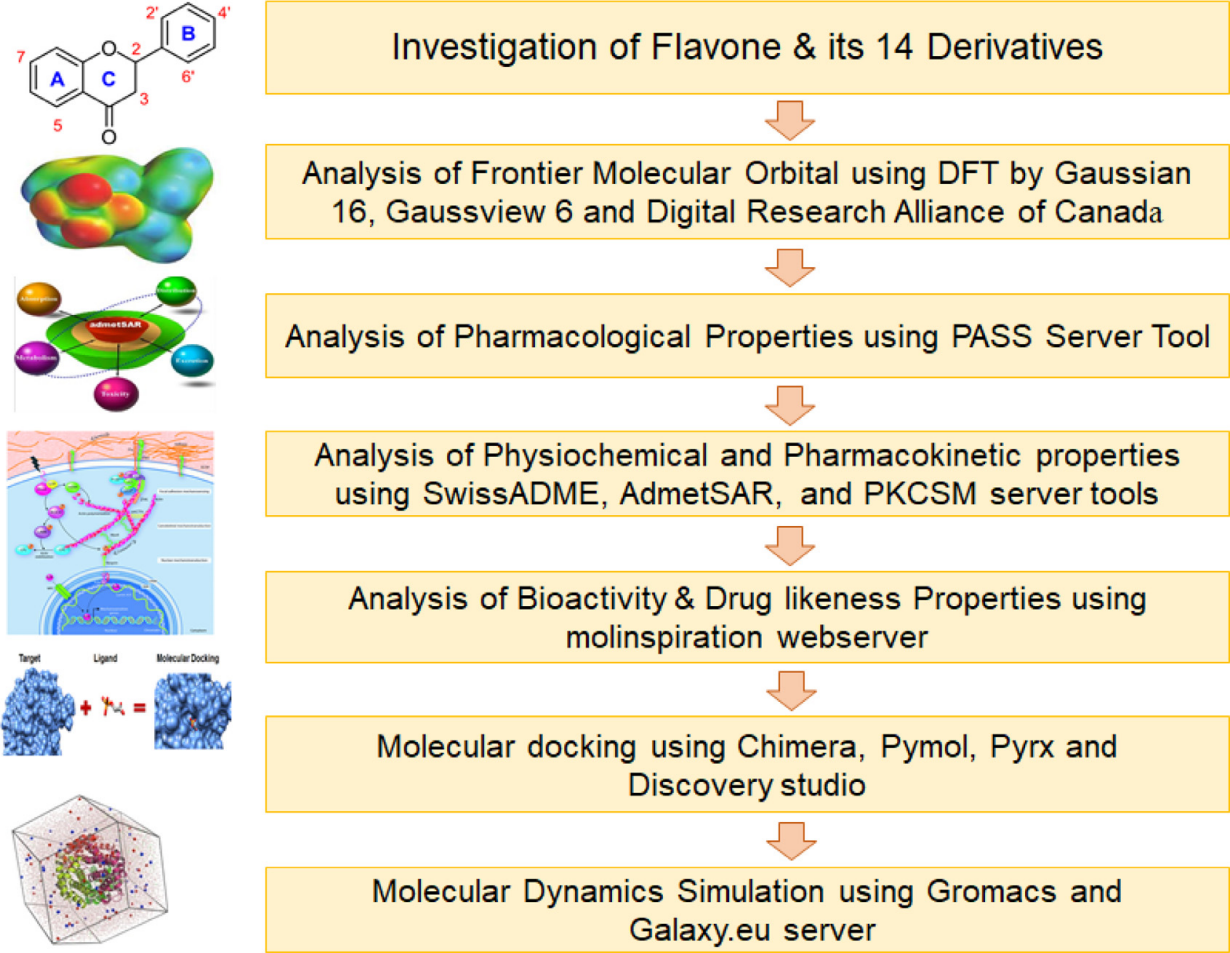

## Methodology

### Computational analysis

To explore potential variations in flavone and its derivatives, we initiated a screening process for compounds labeled (**1 – 14**) within the PubChem molecular database. We then acquired their SDF structures and employed ChemDraw to depict these derivatives, as illustrated in Fig. 1 visually. This method facilitated the identification of isomers and their divergence, ultimately creating innovative and novel compounds. Subsequently, optimization procedures were conducted using Gaussian16 to refine the geometries of the structures (**1 – 14**) [42]. These optimizations are detailed in Tables **S1** to **S14** and were performed at different levels of theory, specifically B3LYP/6–31G(d,p), B3LYP/6–311G(d,p), and B3LYP/6–311++*G*(d,p) [43], [44], [45]. Frequency calculations were also carried out to confirm that all structures were in their energy minima. Previous studies [46], [47], [48], [49], [50], [51], [52] have demonstrated the accuracy and efficiency of using the B3LYP/6–31G(d,p) level of theory. The analysis of frontier molecular orbital (FMO) energy distribution, spanning from the highest occupied molecular orbital (HOMO) to the lowest unoccupied molecular orbital (LUMO), along with the generation of molecular electrostatic potential (MEP) maps, was performed using GaussView 6 software. For a thorough visual presentation, the Supplementary Information (SI) encompasses optimized HOMO, LUMO, MEP maps, Natural Bond Orbital (NBO) data, and all structures pertinent to docking and molecular dynamics (MD), as displayed in Figs. S1 to S18 within the SI.

Additionally, Table 1a, Table 1b shows the dipole moment and polarization values obtained from the Gaussian 16 log files, which were analyzed using Gaussview 6.0. In addition, several chemical descriptors were computed for each flavonoid, such as the energy gap (Egap), ionization potential (IP), electron affinity (EA), electronegativity (χ), chemical potential (µ), hardness (η), softness (σ), and electrophilicity (ω), using the equations provided in references [53], [54], [55], [56], [57], [58].

$$\begin{array}{cc} & E_{\text{Gap}}\left(\text{eV}\right)=\left(E_{\text{LUMO}}-E_{\text{HOMO}}\right);\;\text{IP}\;\left(\text{eV}\right)=-E_{\text{HOMO}};\;\text{EA}\left(\text{eV}\right)=-E_{\text{LUMO}}\\ & \chi \left(\text{eV}\right)=\left(\text{IP}+\text{EA}\right)/2;\;\mu =-\chi ;\;\eta =\left(\text{IP}-\text{EA}\right)/2;\;\sigma =1/\eta ;\;\omega =\mu^{2}/2\eta \end{array}$$

where µ and η were used to derive the electrophilicity indexes. Furthermore, to obtain accurate energy values and atomic charges, the single point energy and natural bond orbital (NBO) are used [57,58].

### Analysis of physicochemical and pharmacokinetic properties

To ensure that a drug achieves an appropriate concentration at its target site, ADMET properties and a drug's adherence to the Lipinski rule must be evaluated. In this study, three web-based tools, SwissADME, AdmetSAR, and PKCSM, were used to predict the ADMET properties of flavonoids and check if they violated any rule. SwissADME utilizes powerful predictive abilities to determine the physicochemical properties of drug candidates, while AdmetSAR employs five quantitative regression models to provide the most accurate drug prediction outcome. PKCSM uses a distance-based approach to compute a drug's potency, safety, and pharmacokinetic properties. The physicochemical and drug-likeness properties of the flavonoids are presented in Table 2, and the canonical SMILES of each flavonoid were entered into both server tools to obtain the ADMET values. Additionally, the Molinspiration Chemoinformatics server tool was utilized to predict the downstream biological activity of flavonoids when interacting with various proteins in different target pathways, such as GPCR, KI, ICM, NRL, PI, and EI [58], [59], [60].

### Pharmacological properties

The Prediction of Activity Spectra of Substances (PASS) server tool can predict various pharmacological activities of compounds, such as toxicity, antibacterial, antifungal, mutagenicity, and more [61] This tool can predict approximately 300 pharmacological effects and can aid in identifying potential organic drug candidates. Pass Prediction was employed to analyze the flavonoids in this study, and the Pass Online Server tool was used for this purpose [62], [63], [64] This web tool provides access to biochemical information on compounds approved for medicinal use in the USA and Russian Federation [63,64]. The results of the analysis are presented in Table S15 in the SI.

### Molecular docking

#### Preparation of target protein

In order to assess the efficacy of a drug in preventing the progression of liver disease, it is crucial to determine its capacity to bind to a cancer-promoting receptor or oncoprotein. This study focused on six critical proteins, including epidermal growth factor protein (PDB: 4UV7), HER2 protein (PDB: 7JXH), farnesyl diphosphate synthase protein (PDB: 1YQ7), hematopoietic prostaglandin D synthase (PDB: 1V40), deoxycytidine kinase protein (PDB: 1P60), and Keap1 on Nrf2 repressor protein (PDB: 1 × 2 J). The 3D crystal structures of these proteins were obtained from the RCSB Protein Data Bank (PDB), the largest archive of thousands of sequenced proteins available for research in various formats [65]. The crystal structures were validated using X-ray diffraction to ensure the quality of the primary protein structure. The structures of all flavonoids were also optimized using UCSF Chimera software version 1.16 [66]. The histidine protonation state was set, and the standard residue was kept at the default AMBER ff14SB for both the polysaccharides and proteins. The Gasteiger method was used as the charged method and applied to other residues of each protein. The protein structure was prepared by removing ligands, water molecules, and metal ions. Finally, Kollman charges were added to the protein molecule before being converted to PDBQT format by the AutoDock Tools (v.1.5.6) package of Pyrx software. The active sites of all the proteins were determined using the CastP server tool. The active sites of the two proteins are shown in Table 5. The rest are given in the supplementary information. The PDB ID: 1p60 was chosen as it is a remarkable liver disease biomarker [67]. Keap1 on the Nrf2 repressor protein (PDB: 1 × 2 J) helps to elevate the accumulation of Nrf2 in the nucleus and protects hepatocytes against acute drug toxicity and inflammatory liver injuries [68]. Several liver diseases are associated with increased hepatic farnesyl diphosphate synthase in mice. Moreover, (PDB: IYQ7) is also suppressed when liver polyunsaturated fatty acids increase [69,70] (PDB: 1V40) is associated with hepatic inflammation and has therapeutic potential [71] (PDB: 4UV7) has shown increased activity and associations with liver disease therapy. Finally, recent studies have found that the Her2 protein (PDB: 7XHJ) has been shown to have a positive correlation with liver diseases [72].

#### Preparation of ligands

The flavonoids and reference drugs (cianidanol and genistein) were obtained in SDF format from the PubChem database (https://pubchem.ncbi.nlm.nih.gov/) [67], [68], [69], [70]. The chemical structures of the synthesized flavonoid ligands were optimized using GaussView 6 software with the Gaussian 16 package at B3LYP/6–31G(d,p). The structures were then subjected to energy minimization and converted to PDBQT format using the OpenBabel plugin of PyRx 0.8 software (https://pyrx.sourceforge.io/) [71].

#### Protein-ligand docking

The docking simulations were carried out using PyRx version 0.8 software with AutoDock 4 and AutoDock Vina tools for ligand‒protein interaction [68], [69], [70], [71]. Prior to docking, the flavonoid structures were energy minimized with the Gasteiger method using UCSF Chimera software and then converted to PDBQT format using PyRx. For complete protein surface coverage during docking, a grid box was set up for each protein. The protein‒ligand complexes were stable throughout the docking process and were verified by redocking. UCSF Chimera software was used to identify amino acid interactions at the docking site, while PyMOL version 2.5 was used to generate molecular docking images [72]. Moreover, BIOVIA Discovery studio was used to recognize 2D protein interactions between the ligand and protein and to examine the hydrogen density surrounding the interacting residues with the protein [73]. The results of the docking studies are presented in Table 4.

#### Molecular dynamics simulation

To investigate the interactions between proteins and ligands and better understand the structure and function of biological macromolecules, molecular dynamics (MD) simulations were employed using the GROMACS version 2021.6 package [74], [75], [76]. The AMBER99SB force field was used to describe atomic interactions in the system. Although computationally intensive, MD simulations provide more precise insight into complex systems than docking and can reveal features inaccessible by experimental methods [77,78]. The complex with the lowest binding and negative binding affinity for flavonoid (**7)** was used to conduct MD simulations for PDB: 1 × 2 J and PDB: 1P60. Protein topology parameters using the Galaxy European Server [79]. The system was solvated in a triclinic box of SPC water molecules and neutralized with Na^+^ ions to achieve default salt concentrations [79], [80], [81]. Using the leap-frog algorithm, we equilibrated the system through a position-restrained dynamic simulation (NVT) at 300 K for 3000 ps. A production run was conducted for an additional 3000 ps at the same temperature and pressure settings. The MD trajectories were visualized and analyzed using VMD, PyMol and GROMACS programs [74], [75], [76], [77], [78], [79], [80], [81]. MD simulation for the docked compounds was performed for 100 ns at 298 K. Moreover, gmx rms, gmx gyrate, and gmx rmsf utilities were used for root-mean-square-deviation (RMSD), radius-of-gyration (Rg), and root-mean-square fluctuation (RMSF) analysis, respectively. We also employed the GROMACS utility ‘gmx hbond’ to perform hydrogen bond analysis, and the temperature and potential energy were determined. Additionally, the Bio3D package through the Galaxy European Server was employed for the MD trajectories' principal component analysis (PCA) [77], [78], [79], [80], [81], [82], [83].

## Method validation

### Analysis of frontier molecular orbitals using DFT

**Fig. 2:**
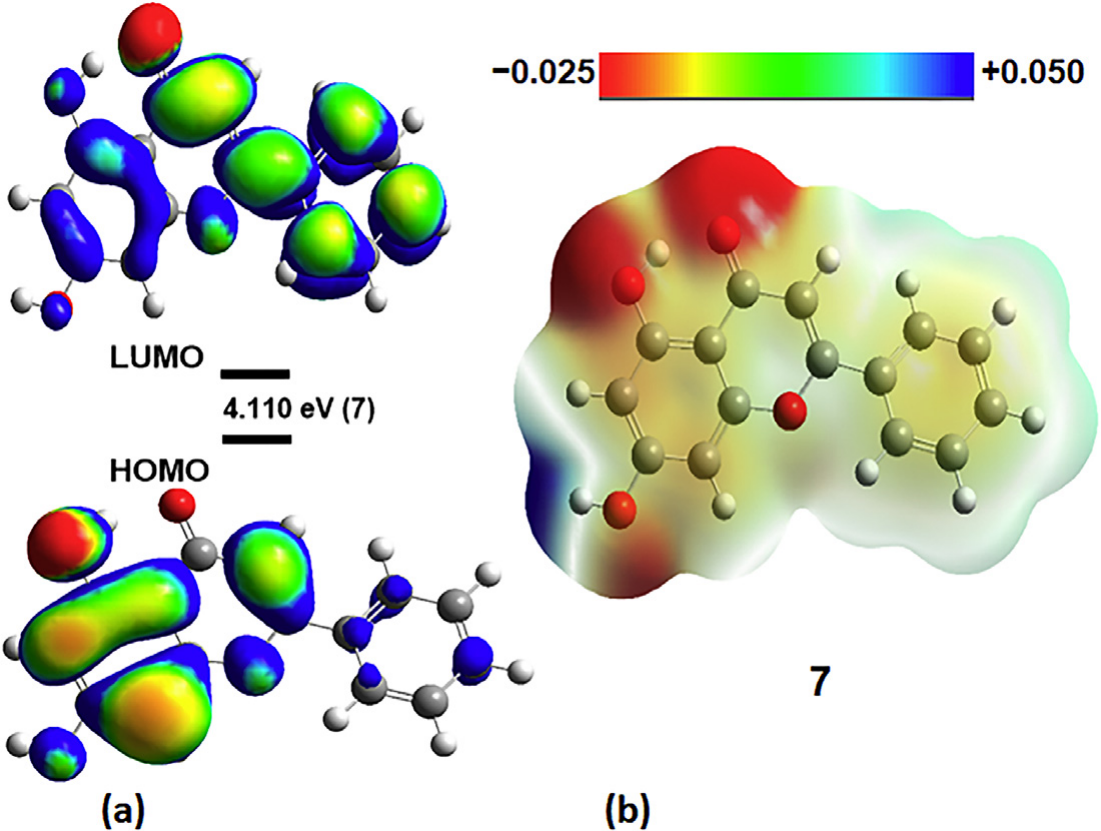
(a) The molecular orbitals of the HOMO and LUMO with Egap. (b) Maps of electrostatic potential (0.02 electrons Bohr^−3^ surface) (red = electron-rich, blue = electron-deficient) for compound **7**. Regions of respective colors indicating electrophilic (blue) and nucleophilic (red) sites and partial nucleophilic sites (yellow regions).

The reactivity and stability properties of flavone and its analogs (**1 – 14**) were assessed using FMO analysis, which provided valuable insights. FMO analysis helped determine the chemical reactivity, softness, hardness, chemical potential, and electrophilic index of the compounds through the HOMO-LUMO energy gap (Egap) parameter. A large Egap indicates high stability and low chemical reactivity, whereas a narrow Egap corresponds to high chemical reactivity and low stability. The energy levels of HOMO and LUMO also play a crucial role in understanding the compounds' electron acceptor and donor properties, respectively, and are critical in chemical reactions. Therefore, FMO analysis is essential for evaluating the reactivity and stability properties of molecules. Furthermore, the ionization potential (IP), electron affinity (EA), electronegativity (χ), chemical potential (µ), global hardness (η), softness (σ), electrophilicity (ω), and dipole moment of all compounds (**1 – 14**) were calculated using the B3LYP/6–31G(d,p) level and are presented in Table 1a (see Figs. S1 to S18 in the SI). The Hartree-Fock model describes the movement of electrons from the ground state to the excited state in atomic orbitals during chemical reactions in a biochemical system [84]. A more significant energy gap indicates a lower electronic transition of the electron, resulting in decreased chemical reactivity of the molecules. Conversely, a smaller gap exhibits a higher atomic system and chemical reactivity, closely related to binding with the target protein [84]^.^ In Table 1, the energy gaps of all compounds fall within the range of 3.57 eV to 4.11 eV. Adding halogens to flavonoids did not result in any significant difference in the energy gap values compared to mono- and di-hydroxyflavones, indicating that group 7 elements do not play an essential role in the binding site of the receptor protein. Fig. 2 displays the HOMO-LUMO orbitals of flavonoids (**7**), while the remaining figures can be found in the Supporting Information (see Figs. S1 to S5). NBO charge structures for compounds (**1 – 14**) are shown in Figs. S6 to S11 in the SI.

**Table 1a:** Computed HOMO and LUMO energies, IP, EA, electronegativity (χ), chemical potential (µ), global hardness (η), softness (σ), electrophilicity (ω), and dipole moment (Debye) of all flavones (**1 – 14**) at 298.15 K using the B3LYP/6–31G(d,p) level.^a^

	E_LUMO_	E_HOMO_	E_gap_	IP	EA	χ	µ	η	σ	ω	dipole
Compound	(eV)	(eV)	(eV)	(eV)	(eV)	(eV)	(eV)	(eV)	(eV)	(eV)	(D)
**1**	−6.355	−1.801	4.554	6.355	1.801	4.078	−4.078	2.277	0.439	3.651	4.183
**2**	−5.991	−1.977	4.014	5.991	1.977	3.984	−3.984	2.007	0.498	3.965	5.014
**3**	−6.041	−1.786	4.25	6.041	1.786	3.913	−3.913	2.127	0.451	3.599	3.111
**4**	−5.823	−1.276	4.547	5.823	1.276	3.549	−3.549	2.273	0.439	2.771	4.273
**5**	−5.605	−1.759	3.682	5.605	1.759	3.682	−3.682	1.923	0.52	3.524	3.811
**6**	−5.709	−1.952	3.83	5.709	1.952	3.831	−3.831	1.878	0.532	3.907	4.522
**7**	−6.001	−1.891	3.946	6.001	1.891	3.946	−3.946	2.055	0.486	3.788	3.688
**8**	−6.641	−2.161	4.401	6.641	2.161	4.401	−4.401	2.241	0.446	4.321	6.042
**9**	−6.563	−2.232	4.397	6.563	2.232	4.397	−4.397	2.165	0.461	4.465	3.755
**10**	−5.631	−1.685	3.658	5.631	1.685	3.658	−3.65	1.973	0.506	3.391	5.685
**11**	−5.541	−1.736	3.638	5.541	1.736	3.638	−3.638	1.902	0.525	3.479	5.122
**12**	−5.546	−1.624	3.585	5.546	1.624	3.585	−3.585	1.961	0.509	3.276	2.511
**13**	−5.554	−0.558	3.056	5.554	0.558	3.056	−3.056	2.498	0.401	2.398	5.031
**14**	−5.983	−1.975	3.979	5.983	1.975	3.979	−3.979	2.004	0.499	3.951	9.748
**Tropoflavin**	−6.034	−1.791	3.913	6.034	1.791	3.913	−3.913	2.121	0.471	3.619	5.624
**Taxifolin**	−5.845	−1.632	4.215	5.845	1.632	4.215	−4.215	2.106	0.474	4.218	4.305

a 7,8-dihydroxyflavone (Tropoflavin) and Dihydrquercetin (Taxifolin).

The investigation of the compounds reveals that they all display a higher hardness (η) than softness (σ). The electrophilicity index, an essential parameter for pinpointing reactive sites, depends on the electron transfer between the acceptor (LUMO) and the donor (HOMO), along with the corresponding energy changes [84], [85], [86]. Take flavonoid (**7**) as an example, its electrophilicity index is 3.946 eV. This measurement acts as a benchmark for evaluating the biological activity of flavonoids (**7**) and gauging their performance in molecular docking studies. Notably, flavonoid (**7**) has a higher eV value than the reference drug, which is 3.913 eV. It also shows a lower Debye value of 3.688, signifying its effectiveness. These findings indicate a stronger interaction between flavonoid (**7**) and the active site amino acids of the target protein, reinforcing its potential as a potent agent.

Structural parameters, encompassing bond lengths and angles for these compounds, were computed using the B3LYP level of theory with varying basis sets (6–31G(d,p), 6–311G(d,p), and 6–311++*G*(d,p)). To assess the precision of the selected method, we conducted a comparative analysis between the computational results and the experimental data. This comparison aimed to identify discrepancies or variations between the calculated and experimental results [87], [88], [89]. Table 1b displays discrepancies in structural parameters resulting from methodological disparities in the analysis of single crystals of commercially available compounds, namely, compounds (**1)** and (**7)**. These crystals were obtained through room temperature X-ray diffraction, and detailed information on crystal data, data collection, and structure refinement can be found in previously published reports [87], [88], [89]^.^ Notable differences were observed when comparing the optimized structures with the crystal structures of these compounds. Specifically, variations were identified in the position of the carbon six (C5-C6 double bond) in the inner ring, as well as differing conformations in the outer ring, particularly in the C1-C2 and C1-C6 regions, along with bond angles, as indicated in Table 1b. These variations resulted in calculated bond lengths within the ring structure that deviated from the crystallographic data.

**Table 1b:** Selected bond distances (Å) and angles (deg) for flavone derivatives.

Bond Type	B3LYP/6–31G(d,p)	B3LYP/6–311G(d,p)	B3LYP/6–311++*G*(d,p)	Expt^a^
**1**	**Bond Distance (Å)**			
C5-C6	1.4077	1.408	1.40311	1.398
C1-C6	1.40527	1.4029	1.40301	1.481
C1-C2	1.47547	1.47514	1.47512	1.455
	**Bond Angle (deg)**			
C4-C5-C6	121.56	121.57	121.81	121.50
C1-C6-C2(phenyl ring)	118.81	118.76	118.80	113.28
C6-C1-C2(main-phenyl)	120.75	120.82	120.75	122.78
**7**	**Bond Distance (Å)**			
C5-C6	1.4565	1.4572	1.4572	1.386
C1-C4	2.744	2.739	2.7422	2.899
C1-C2	1.3733	1.372	1.3750	1.358
	**Bond Angle (deg)**			
C4-C5-C6	121.30	121.43	121.63	120.14
C1-C6-C2(phenyl ring)	123.40	122.80	122.60	122.70
C6-C1-C2(main-phenyl)	122.79	122.80	122.50	120.40

The electrostatic potential maps offer qualitative insights into the net electrostatic impact at a point because of the total charge distribution. These maps help identify electrophilic and nucleophilic sites, where electrophilic sites act as electron acceptors and nucleophilic sites act as electron donors. ESP contour maps demonstrate the electron distribution that favors a low potential for high wavelengths and a high potential for low wavelengths. For all the compounds analyzed, regions of respective colors indicating electrophilic (blue) and nucleophilic (red) sites and partial nucleophilic sites (yellow regions) were identified, indicating total density [85]. The MEP maps of the molecules, including Fig. 2 and Figs. S12-S14 in the SI, can help determine the regions of electrophilicity and nucleophilicity.

#### Analysis of the pharmacological characteristics of flavonoids

In this study, multilevel neighborhoods of atoms (MNA) descriptors in the prediction of activity spectra for substances (PASS) were employed to conduct a comprehensive analysis of the pharmacological properties of 14 flavone derivatives (**1 – 14**). Flavonoids are known for their diverse biological activities and ability to modulate reactions by binding to downstream proteins. The PASS predictions for each compound were based on various parameters, including their potential to affect membrane integrity, act as membrane permeability inhibitors, exhibit anticarcinogenic properties, and display antioxidant activities. The Pa and Pi values for these parameters ranged from 0.001 to 1.000. A Pa value exceeding 0.6 indicated significant biological activity in *in vitro* experiments.

Notably, flavonoids (**1, 7**, and **9)** demonstrated Pa values ranging from 0.618 to 0.989 for all four parameters, as detailed in Table S15 in the Supplementary Information (SI). These findings suggest the potential for these compounds to maintain good cell membrane integrity and block cell permeability effectively. Flavonoid (**9)** exhibited higher values than the control drug and exhibited noncarcinogenic and antioxidant properties. However, the membrane integrity and permeability values for Flavonoid (**11)** could not be determined. Furthermore, flavonoids (**1, 2, 6, 8, 9**, and **11)** scored below the cutoff value for anticarcinogenic activity, indicating that they may be susceptible to carcinogenic substances. Nonetheless, all compounds demonstrated scores above the cutoff value for being membrane integrity agonists and membrane permeability inhibitors, suggesting their effectiveness in these two parameters. In summary, the PASS Prediction analysis provides valuable insights into the potential biological activities of the studied flavonoids. This information can assist in identifying promising candidates for further investigation and drug development.

Cell survival, structural integrity, and polarity heavily rely on the stability and functionality of cell membranes. Compounds that serve as membrane integrity agonists or contribute to membrane stabilization can avert apoptosis, metabolic complications, and nonalcoholic fatty acid disease. Recent research has identified the targeting of mitochondrial membrane permeability as a promising pharmacological approach for liver diseases, including fatty acid liver disease and cardiovascular disorders [90], [91], [92]. Besides these attributes, an effective drug should have anticarcinogenic and antioxidant properties to neutralize radical formation and aid chemotherapy. Flavonoids, especially those containing ortho-dihydroxy groups on the C ring of flavones, have shown significant effectiveness in scavenging radicals, making them crucial for antioxidant and anticancer properties [92,93]. In conclusion, compounds that stabilize cell membranes or function as membrane integrity agonists are essential for cell survival, and those with antioxidant and anticancer properties can be beneficial supplements to chemotherapy [92].

#### Analysis of physiochemical and drug likeness properties of flavonoids

The physicochemical properties must be examined to assess whether flavonoid derivatives (**1 – 14**) adhere to Lipinski, Veber, Egan, Muegge, Ghose and guidelines. Flavonoids (**14**) showed 1 violation in all the druglikeness parameters, making them unsuitable options for drug candidates. Lipinski's guidelines require that a compound satisfy five criteria before it can be administered orally, including a molecular weight (MW) of less than 500 g/mol, an octanol-water partition coefficient (logP) below 5, no more than 5 hydrogen bond donors (HBDs), no more than 10 hydrogen bond acceptors (HBAs), and a topological polar surface area (TPSA) of no more than 140 Å^2^. Veber's guidelines further specify that the number of rotatable bonds (nrotb) must be less than 10, and TPSA must be less than or equal to 140 Å^2^ (consistent with Lipinski's guidelines) for good drug bioavailability. The study utilized SwissADME to assess the compliance of the most biologically active compounds (**1 – 14**) with these guidelines. The results showed that all compounds complied with the specified Lipinski and Veber guidelines (see Table 2). In addition, the drug-like score of 1 indicated that all compounds met the criteria for developing novel medications with good theoretical evidence, as they exhibited high agreement (less than 6) for drug compounds with MW less than 500 g/mol, MLOGP 4.15, and Log S (ESOL) within the established guidelines.

**Table 2:** *In silico* prediction of physicochemical parameters for the flavone and its derivatives (**1**-**14**).^a^

Physiochemical properties							Drug-likeness				
Ligand	MW	MR	HBD	HBA	nroth	TPSA	Lipinksi	Veber	Ghose	Egan	Muegge
**1**	254.25	71.97	2	4	1	70.67	0 violation	Yes	Yes	Yes	Yes
**2**	238.24	69.94	1	3	1	50.44	0 violation	Yes	Yes	Yes	Yes
**3**	238.24	69.94	1	3	1	50.44	0 violation	Yes	Yes	Yes	Yes
**4**	238.24	69.94	1	3	1	50.44	0 violation	Yes	Yes	Yes	Yes
**5**	238.12	69.94	1	3	1	50.44	0 violation	Yes	Yes	Yes	Yes
**6**	304.25	74.76	5	7	1	127.45	0 violation	Yes	Yes	Yes	Yes
**7**	304.25	74.76	5	7	1	127.45	0 violation	Yes	Yes	Yes	Yes
**8**	222.24	67.92	2	2	1	30.21	0 violation	Yes	Yes	Yes	Yes
**9**	464.40	110.16	8	12	4	210.51	0 violation	Yes	Yes	Yes	Yes
**10**	436.41	106.14	7	10	7	177.14	0 violation	Yes	Yes	Yes	Yes
**11**	291.13	77.94	0	2	1	30.21	0 violation	Yes	Yes	Yes	Yes
**12**	335.58	80.63	0	2	1	30.21	0 violation	Yes	Yes	Yes	Yes
**13**	300.28	80.98	4	6	1	111.13	0 violation	Yes	Yes	Yes	Yes
**14**	254.24	71.97	2	4	41	70.67	1 violation	1	1	1	1
**Tropoflavin**	254.24	71.97	2	4	1	70.67	0 violation	Yes	Yes	Yes	Yes
**Taxifolin**	304.25	74.76	5	7	1	127.45	0 violation	Yes	Yes	Yes	Yes

a MW, molecular weight; MR, molar refractivity, lipophilicity (O/W); HBD, number of hydrogen bond donors; HBA, number of hydrogen bond acceptors; nroth, number of rotatable bonds; TPSA, topological polar surface area (Å^2^); standard reference drugs such as tropoflavin and taxifolin.

Table 2 presents the physicochemical and drug-likeness properties of 16 flavonoids. Notably, flavonoid (**9**) contains the highest number of hydrogen bond donors and acceptors, while flavonoid (**14**) has the most rotatable bonds, which can improve oral bioavailability by increasing compound flexibility. Molecular weight is crucial to a compound's ability to reach its target site effectively. All flavonoids meet the criterion of having a molecular weight no greater than 450–500 g/mol. Flavonoids (**1, 6**, and **7**) have acceptable topological polar surface area (TPSA) values, which determine a compound's permeability by passive diffusion, blood‒brain barrier permeability, and peripheral circulation restriction. Flavonoids (**6**) and (**13**) have acceptable numbers of rotatable bonds, while flavonoids (**1, 8, 11**, and **12**) have out-of-range TPSA values. All compounds except flavonoid (**11**) have good molar refractivity.

To efficiently develop a new drug, it is important to assess its pharmacokinetic properties, which include absorption, distribution, metabolism, excretion, and toxicity (ADMET). The study utilized admetSAR to evaluate the ADMET characteristics of the 14 active flavonoids (**1 – 14**). The results are presented in Table 3, which includes the evaluation of seven critical ADMET properties, including human intestinal absorption (HIA), blood‒brain barrier (BBB) penetration, water solubility, cytochrome P450 enzyme (CYP3A4 and CYP2C19) inhibition, and hERG inhibition. It is important to note that crossing the blood‒brain barrier can be beneficial for drugs that target the central nervous system (CNS) but may be harmful to those with little effect on the CNS. BBB penetration is classified as high (>2), medium (2–0.1), or low (<0.1). Table 3 shows that all flavonoids, except flavonoid (**14)**, have high HIA values, with (**1, 8**, and **9)** having complete absorption rates. However, none of the compounds, except flavonoids (**8)** and (**9)**, can penetrate the BBB. Furthermore, all flavonoids have good water solubility. Regarding cytochrome P450 enzymes, all flavonoids except flavonoid (**1)** show positive/negative inhibition of CYP3A4, while all flavonoids show negative/positive inhibition of CYP2C19. In addition, all flavonoids, including the control, show negative inhibition of the hERG gene.

**Table 3:** *In silico* prediction of selected ADMET parameters for all flavones (**1**-**14**).^a^

Ligand	bHIA	bBBB	bWater Solubility	bCYP3A4 inhibition	bCYP2C19 inhibition	bhERG_pIC5
**1**	+(1.000)	-(0.5250)	-(3.425)	+(0.6321)	+(0.8993)	-(0.7082)
**2**	+(0.9964)	-(0.7750)	-(3.171)	-(0.5182)	+(0.7571)	-(0.8393)
**3**	+(0.9964)	-(0.7750)	-(3.171)	-(0.5182)	+(0.7571)	-(0.8205)
4	+(0.9964)	-(0.7750)	-(3.171)	-(0.5182)	+(0.7571)	-(0.8427)
**5**	+(0.9769)	-(0.8250)	-(3.259)	-(0.7012)	+(0.7715)	-(0.8935)
**6**	+(0.9769)	-(0.8250)	-(3.259)	-(0.7012)	+(0.7715)	-(0.8902)
**7**	+(0.9671)	-(0.7650)	-(2.777)	-(0.9580)	+(0.7043)	-(0.8004)
**8**	+(1.000)	+(0.6250)	-(5.089)	-(0.5725)	+(0.8086)	-(0.7440)
**9**	+(1.000)	+(0.5750)	-(4.238)	-(0.7648)	+(0.7373)	-(0.6546)
**10**	+(0.9071)	-(0.7750)	-(2.999)	-(0.6951)	+(0.9025)	-(0.8410)
**11**	+(0.9577)	-(0.8000)	-(3.186)	-(0.7179)	+(0.6827)	-(0.7627)
**12**	+(0.9071)	-(0.7750)	-(2.999)	-(0.6951)	+(0.9025)	-(0.7830)
**13**	+(0.7510)	-(0.5750)	-(1.304)	-(0.8751)	+(0.8258)	-(0.4454)
**14**	-(0.5116)	-(0.7750)	-(2.449)	-(0.9193)	+(0.9289)	-(0.5000)
**Tropoflavin**	+(0.9569)	-(0.7750)	-(3.080)	-(0.7054)	+(0.6965)	-(0.9154)
**Taxifolin**	+(0.9071)	-(0.7750)	-(2.999)	+(0.6951)	-(0.9025)	-(0.7322)

a HIA: human intestinal absorption (%); BBB: blood‒brain barrier penetration; PPB: plasma protein binding; CYP3A4: Cytochrome P4503A4; CYP2C19: Cytochrome P4502C19; hERG: human ether-a-go-go-related gene hERG inhibition potential (pIC_50_), the potential risk for inhibitors ranges from 5.5 − 6.

b The values are determined using admetSAR and SwissADME.

#### Analysis of bioactivity and pharmacological properties of flavonoids

The study also utilized Molinspiration Chemoinformatics to evaluate the interactions between flavonoids and various biological targets, including G protein-coupled receptors (GPCRs), ion channel modulators (ICMs), kinase inhibitors (KIs), nuclear receptor ligands (NRLs), protease inhibitors (PIs), and enzyme inhibitors (EIs). A bioactivity score of 0.00 indicates a molecule's potential for exhibiting good downstream biological activity. On the other hand, a score between −0.50 and 0 suggests good biological activity, and a score less than −0.50 indicates inactivity. Flavonoid (**9)** from Table 4 demonstrated the best activity for all parameters and is the most promising candidate. Except for flavonoids (**11** – **13**), all flavonoids showed good bioactivity in at least three mechanisms, thus narrowing down the list of potential drugs to flavonoids (**7**), which also has an excellent score in Table 2. Overall, the findings indicate that the pharmacological activity of drug compounds may involve multiple mechanisms. Their interactions with GPCR ligands, nuclear receptor ligands, and inhibitors of proteases and other enzymes may play a crucial role in their physiological effects.

**Table 4:** Shows the bioactivity and pharmacological properties of all flavones (**1 – 14**).

Ligand	GPCR	ICM	KI	NRL	PI	EI
**1**	−0.30	−0.21	−0.12	−0.18	−0.52	0.03
**2**	−0.17	−0.06	0.06	0.15	−0.34	0.21
**3**	−0.16	−0.13	0.04	0.10	−0.43	0.15
**4**	−0.20	−0.17	0.00	0.10	−0.45	0.14
**5**	−0.19	−0.20	0.11	0.15	−0.35	0.24
**6**	−0.18	−0.27	0.10	0.11	−0.44	0.18
**7**	−0.11	−0.08	0.15	0.30	−0.30	0.26
**8**	−0.17	−0.20	−0.16	−0.21	−0.37	0.08
**9**	−0.31	−0.29	−0.08	−0.22	−0.55	−0.06
**10**	−0.06	−0.19	0.28	0.36	−0.25	0.28
**11**	−0.10	−0.24	0.24	0.18	−0.32	0.19
**12**	0.10	−0.07	−0.07	0.21	0.02	0.29
**13**	0.17	0.17	−0.09	0.26	0.14	0.44
**14**	0.06	−0.04	0.13	0.20	−0.06	0.42
**Tropoflavin**	−0.18	−0.15	0.11	0.08	−0.41	0.22
**Taxifolin**	0.09	0.03	−0.04	0.29	0.05	0.29

#### Molecular docking and postdocking analysis

The process of molecular docking is critical in determining the binding affinity between a ligand and a protein or receptor [93], [94], [95]. This method provides valuable insights into the behavior of compounds and their interactions with the active site residues of a protein, as well as downstream cellular processes. The docking results for 14 flavonoids with six proteins are presented in Table 5, with HER2 (PDB: 7JXH) showing higher binding affinity with all flavonoids than the other proteins. A recent study carried out by Sajjan et al. showed HER2 (PDB: 7JXH) binding affinity to be −8.5 kcal mol^−1^), which is less than our obtained value [90], [91], [92], [93], [94], [95].

**Table 5:** Docking results for flavone derivatives (**1 – 14**) against six protein targets.*^a^*

	Binding affinity (kcal mol^−1^)					
Ligand	EGFR (4UV7)	HER2 (7JXH)	FPPS (1YQ7)	HPGDS (1V40)	DCK (1P60)	Nrf2 (1 × 2 J)
**1**	−7.2	−9.1	−7.9	−8.8	−8.5	−8.2
**2**	−7.2	−8.9	−8.3	−9.2	−7.9	−8.7
**3**	−7	−9.1	−8.3	−8.4	−8.7	−8.6
**4**	−7.7	−8.4	−8.4	−9.3	−7.0	−9.0
**5**	−7.6	−9.0	−8.1	−9.4	−7.7	−8.9
**6**	−7.4	−9.4	−8.6	−8.2	−8.7	−8.9
**7**	−7.5	−9.3	−8.5	−9.2	−8.1	−9.4
**8**	−7.2	−9.1	−6.5	−8.2	−8.0	−8.1
**9**	−6.8	−9.1	−7.6	−8.6	−6.6	−9.2
**10**	−7.5	−9.2	−7.7	−9.2	−8.9	−9.3
**11**	−7.4	−8.8	−6.6	−8.0	−7.5	−8.8
**12**	−7.7	−9.1	−8.6	−8.5	−7.9	−9.3
**13**	−7.7	−8.7	−7.7	−8.3	−7.1	−8.7
**14**	−7.4	−8.8	−7.0	−8.2	−7.7	−9.3
**Tropoflavin**	−7.5	−8.9	−8.4	−9.2	−8.0	−9.3
**Taxifolin**	−7.4	−8.9	−8.7	−8.9	−7.1	−9.4

Flavonoid (**1)** displayed a high binding affinity with EGFR, HER2, FPPS, HGDS, and Keap1 on the Nrf2 protein. At the same time, flavonoid **(6)** demonstrated higher binding affinity than the control with EGFR, HER2, FPPS, and the highest affinity of −9.4 kcal mol^−1^ with (PDB:1 × 2 J) Keap1 protein on Nrf2. The results of (PDB:1 × 2 J) Keap1 protein on Nrf2 were better than the experimental study carried out by Cai et al., where they obtained a value of (−9.1 kcal mol^−1^) [93], [94], [95]. Flavonoids (**8**) and (**12**) had a lower affinity with four out of six proteins, possibly because they have halogens attached to the phenyl ring. The molecular docking study revealed that flavonoid **(7)** had a strong binding affinity for HER2 (−9.5 kcal mol^−1^) and FPPS (−8.5 kcal mol^−1^) compared to the standard reference drug. As shown in Table 5, most of the docking values obtained for flavonoids were more effective than the reference drug, 7,8-dihydroxyflavone (HER2: −8.9 kcal mol^−1^, FPPS: −8.4 kcal mol^−1^, EGFR: −7.5 kcal mol^−1^, HPGDS: −9.2 kcal mol^−1^, DCK: −8.6 kcal mol^−1^ and Nrf2: −9.4 kcal mol^−1^), in terms of their potential for liver treatment and cirrhosis reduction. The molecular docking study revealed that some of the compounds (**1 – 14**) showed a strong binding affinity for multiple targeted proteins, specifically HER2 and Nrf2 proteins. Flavonoids **(3)** and **(5)** showed a binding affinity of −8.7 kcal mol^−1^ for DCK. Nrf2 (PDB:1 × 2 J) showed a range of values within −8.1 to −9.9 kcal mol^−1^. The values obtained for all flavonoids for Nrf2 (PDB:1 × 2 J) were similar to those obtained for the complex compound with DHPA by Cai and his team [90], [91], [92] and the results are given in Table 5. Researchers can optimize compounds and develop safe and effective therapeutic agents by combining computational and experimental methods.

**Fig. 3:**
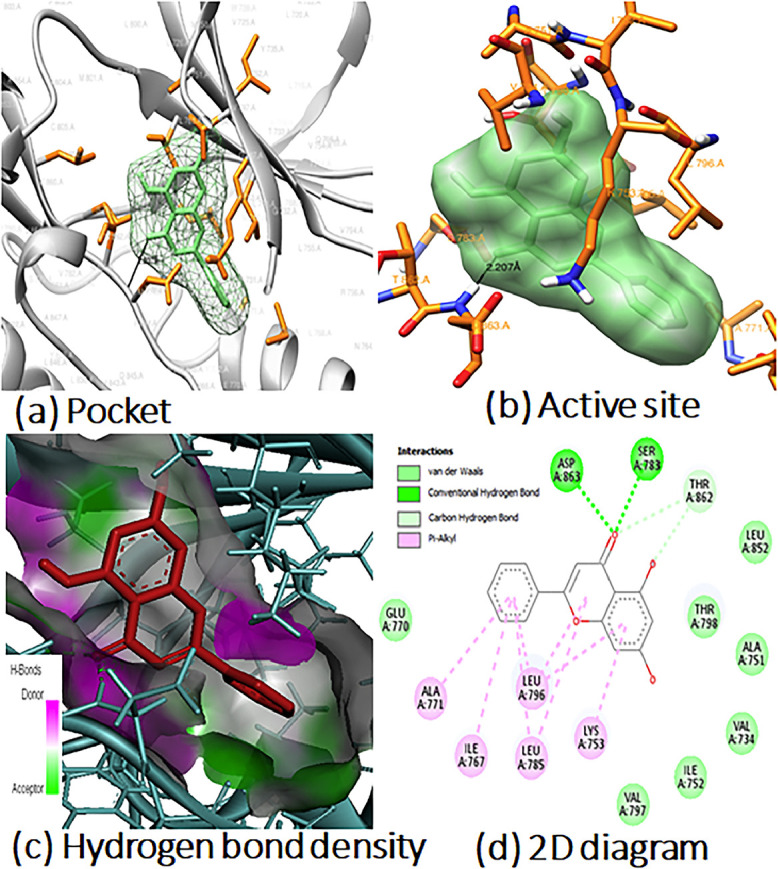
The Molecular docking poses: (a) Ligand in protein pocket; (b) Active site; (c) Hydrogen bonding in solid; (d) Ligand‒protein interaction for 2D diagram of compound 7 with PDB (1 × 2 J).

**Fig. 4:**
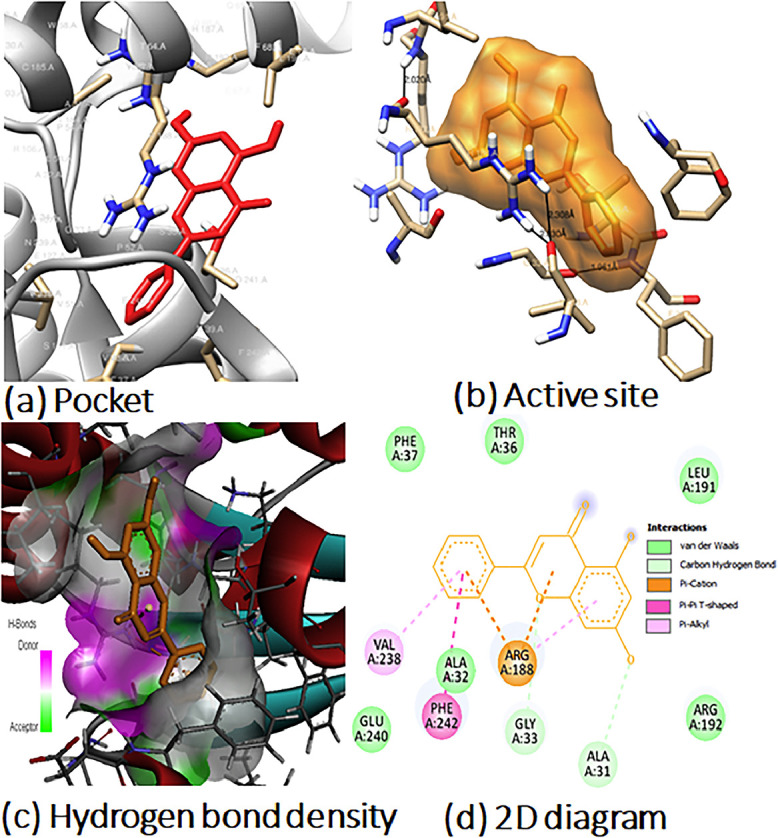
The Molecular docking poses: (a) Ligand in protein pocket; (b) Active site; (c) Hydrogen bonding in solid; (d) Ligand‒protein interaction for 2D diagram of compound **7** with PDB (1P60).

In addition, analyzing the chemical bonds and interactions between the ligand and protein can help explain how the ligand affects the active parts of disease-causing pathogens. The study provides information on bond lengths for each type of bond and residue number. Figs. 3 and 4 show 2D diagrams for flavonoid (**7)** interaction with HPGDS (PDB: 1V40), DCK (PDB: 1P60) and Nrf2 (PDB: 1 × 2 J), including (a) the ligand in the protein pocket, (b) active site residues, (c) hydrogen bond density, and (d) ligand‒protein interaction. The remaining docking pictures can be found in the supplementary information (see Figs. S15-S16 in the SI). The postdocking analysis of all 14 flavonoids and their respective proteins was conducted using various software tools, including UCSF Chimera, PyMOL version 2.5, and BIOVIA Discovery Studio. In Fig. 3, the interacting active site residues are highlighted in orange, while heteroatoms are color-coded, with nitrogen in blue, hydrogen in white, and oxygen in red. Fig. 3a illustrates the binding pocket of Nrf2 (PDB: 1 × 2 J) enclosing flavonoid (**7**), while Fig. 3b, generated using UCSF Chimera, provides a visual representation of the active site residues and their interactions with the ligand. The active site amino acids that interact with the ligand include Asp, Ser, Thr, Leu, Glu, Lys, Ile, Val, and Ala (for details, refer to Table 6 and S16 in the Supplementary Information). In this binding, a total of one hydrogen bond was identified between the ligand and the (PDB:1 × 2 J) active site, with one of them being particularly strong, with a distance of 2.207 Å. Fig. 3c displays various intramolecular bonds in different colors, indicating interactions with the ligand. Two conventional hydrogen bonds occur between Ser and Asp with the oxygen of the phenyl ring, and eight Pi-alkyl bonds form between Ala, Ile, Leu, and Lys on the phenyl ring. Additionally, Thr is involved in two van der Waals forces with two oxygen atoms of the phenyl ring. Fig. 3d highlights the hydrogen bond density within the ligand‒protein pocket, with magenta representing donor hydrogen bonds and parrot green representing acceptor hydrogen bonds, as shown in Fig. 4. In Fig. 4, depicts the binding pocket of DCK (PDB: 1P60) containing flavonoid (**7**), while Fig. 4b showcases the active site residues interacting with (**7**). The active site amino acids interacting with the ligand comprise Trp, Leu, Gly, Tyr, Cys, Met, Arg, and Asp. In this binding, seven hydrogen bonds were detected between the ligand and the (PDB:1P60) active site. Fig. 4c uses various colors to represent different intramolecular bonds, including van der Waals, hydrogen carbon bonds, Pi-alkyl, and conventional hydrogen bonds, which interact with the ligand. Fig. 4d displays the hydrogen bond density within the ligand‒protein pocket.

**Table 6:** Shows the active site residues of proteins (PDB: 1 × 2 J) and (PDB: 1P60).

Active Site of PDB: 1 × 2J	
**Pocket Surface area** Å**^2^**	402.149
**Pocket Volume (SA)** Å**^3^**	419.304
**Active Site Residues**	324 VAL, 364 GLY, 365 LEU, 366 ALA, 367 GLY, 368 CYS, 369 VAL, 415 ARG, 416 ILE, 17 GLY, 418 VAL, 419 GLY, 420 VAL, 462 GLY, 463 VAL, 464 GLY, 465 VAL, 466 ALA, 467 VAL, 509 GLY, 510 ALA, 511 GLY, 512 VAL, 513 CYS, 514 VAL, 556 ALA, 557 LEU, 558 GLY, 559 ILE, 560 THR, 561 VAL, 603 GLY, 604 VAL, 606 GLY, 607 ALA, 608 VAL

**Active Site of PDB:****1P60**	

**Pocket Surface area** Å**^2^**	320.855
**Pocket Volume (SA)** Å**^3^**	356.089
**Active Site Residues**	61 VAL, 62 GLY, 63 SER, 64 THR, 65 GLY, 66 ASP, 69 GLU, 73 MET, 7 SER, 75 GLN, 76 LYS, 78 GLY, 79 GLY, 134 ARG, 135 TYR, 149 GLU, 150 THR, 152 TRP, 153 THR, 156 GLN, 160 THR, 222 ASP. 223 LYS, 223 THR, 224 ASP.

#### Molecular dynamics (MD) simulation

**Fig. 5:**
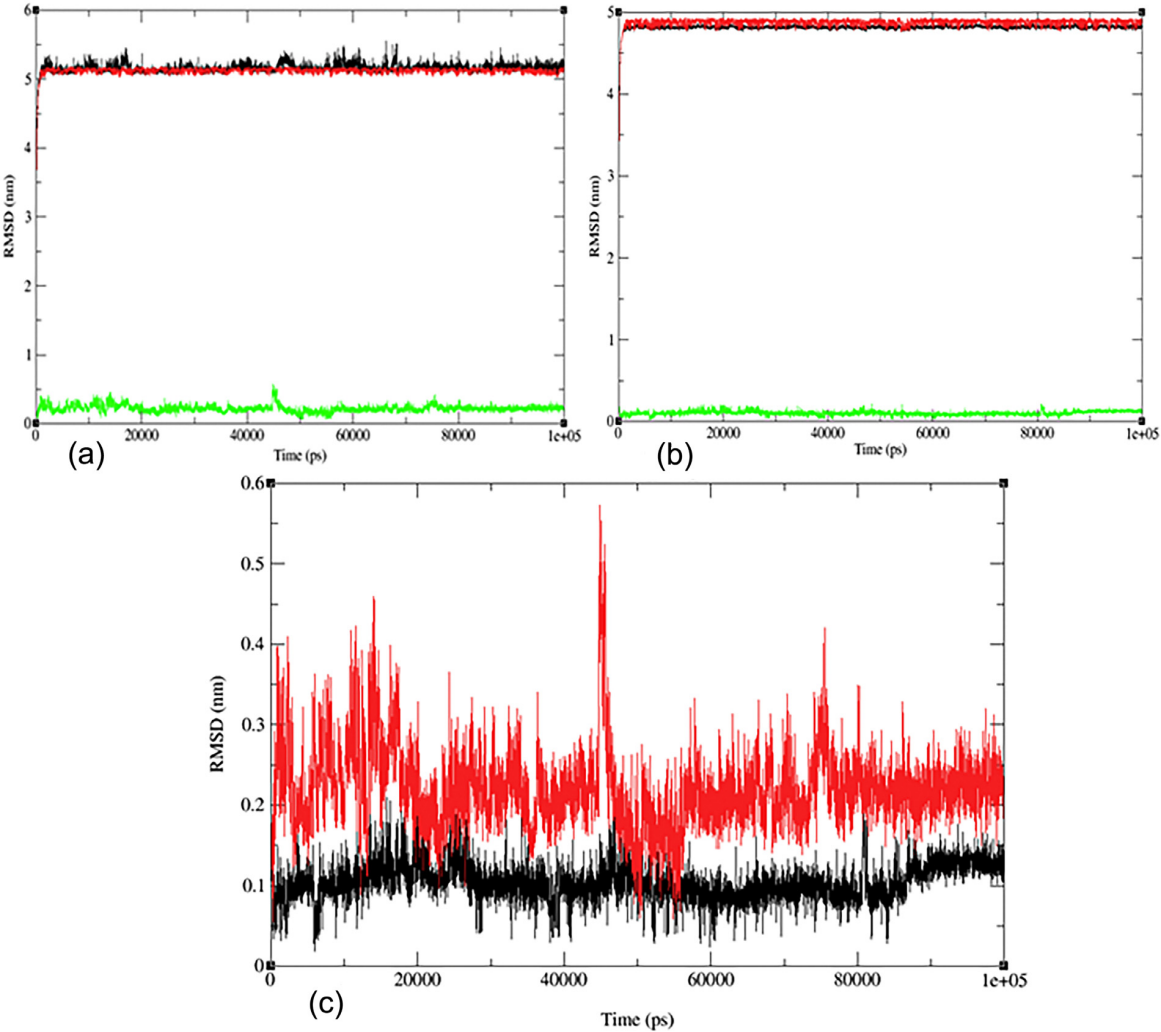
The RMSD evolution (a) for the docked complex, green line (flavonoid **7**, red line with PDB: 1P60, black line); (b) for the docked complex, green line (flavonoid **7**, red line with PDB: 1 × 2 J, black line); and (c) for the merged docked complex between two proteins (PDB: 1P60, red line and PDB: 1 × 2 J, black line) during the 100 ns MD simulation.

To understand the stability and interactions of the protein‒ligand complex of flavonoid (**7**) with Nrf2 (PDB:1 × 2 J) and DCK (PDB:1P60), molecular dynamics simulations were performed for 100 ns, as shown in Fig. 5. The MD simulation results were analyzed using several metrics, such as RMSD, RMSF, Rg values, potential energies, temperature, and hydrogen bonding. The RMSD values of the protein‒ligand complex, ligand, and protein backbone were calculated during the molecular dynamics simulation, as shown in Fig. 5. The results indicated that the RMSD values for (PDB: 1P60), including the protein‒ligand complex, ligand, and water ions, ranged between 0.05 and 0.4 nm (Fig. 5a-c). Meanwhile, the RMSD value for (PDB:1 × 2 J) was within 0.04 to 0.21 nm (Fig. 5a-c), suggesting that the ligand‒protein complex underwent significant structural changes or movement, possibly due to the ligand binding event or conformational change induced by ligand‒protein interactions. More detailed information can be found in the supplementary document (see Figs. S17-S18 in the SI). Fig. 5c displays the RMSD graph of the combined proteins, with the red curve representing the fluctuations of (PDB:1P60) and the black curve depicting the fluctuations of (PDB:1 × 2 J). Undoubtedly, (PDB:1P60) has a higher fluctuation and a steep increase at ∼45,000 ps, while (PDB:1 × 2 J) has no steep increase in fluctuation. This means that both proteins have different coordinates and different structural conformations, indicating differences in stability [92], [93], [94].

**Fig. 6:**
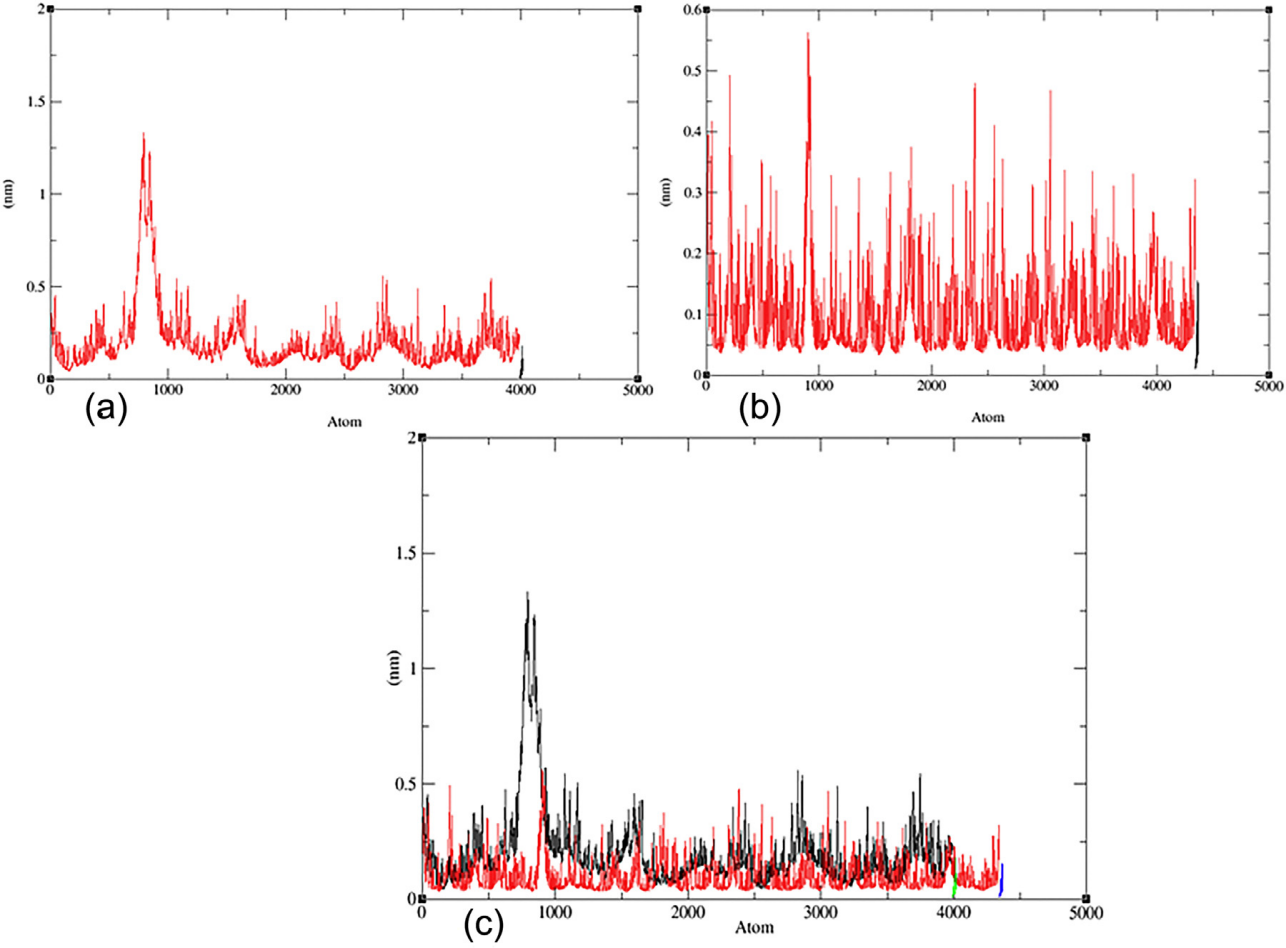
The RMSF evolution (a) for the docked complex (flavonoid **7** with PDB: 1P60); (b) for the docked complex (flavonoid **7** with PDB: 1 × 2 J) and (c) for the merged docked complex between two proteins (PDB: 1P60, red line and PDB: 1 × 2 J, black line).

The RMSF measures the average deviation of individual atoms in a protein from their average positions. In this simulation, separate RMSF values were calculated for the protein and the ligand. Fig. 6 displays the RMSF values of the protein‒ligand complex of Nrf2 (PDB:X2J) and DCK (PDB:1P60) with flavonoids (**7**). The RMSF value for the complex (PDB: 1P60) and flavonoid (**7**) is approximately 0.04 nm with a slight fluctuation in the 700–800 range amino acid atom having a value of approximately 0.14 nm. These findings suggest that the protein and the ligand are relatively stable during the simulation, with only minor fluctuations in atom position. However, fluctuations in certain ligand atoms could be significant and potentially linked to a specific function or interaction of the protein. For the complex (PDB: 1 × 2 J) and flavonoid (**7**), the RMSF value ranges from 0.004 to 0.055 nm, indicating massive fluctuations in certain regions of the protein or the ligand, while others remain relatively rigid. High RMSF values could suggest conformational changes, flexibility, or disorder in the protein or the ligand. These alterations may be due to various factors, such as ligand binding, solvent exposure, or changes in temperature or pH [94], [95], [96].

**Fig. 7:**
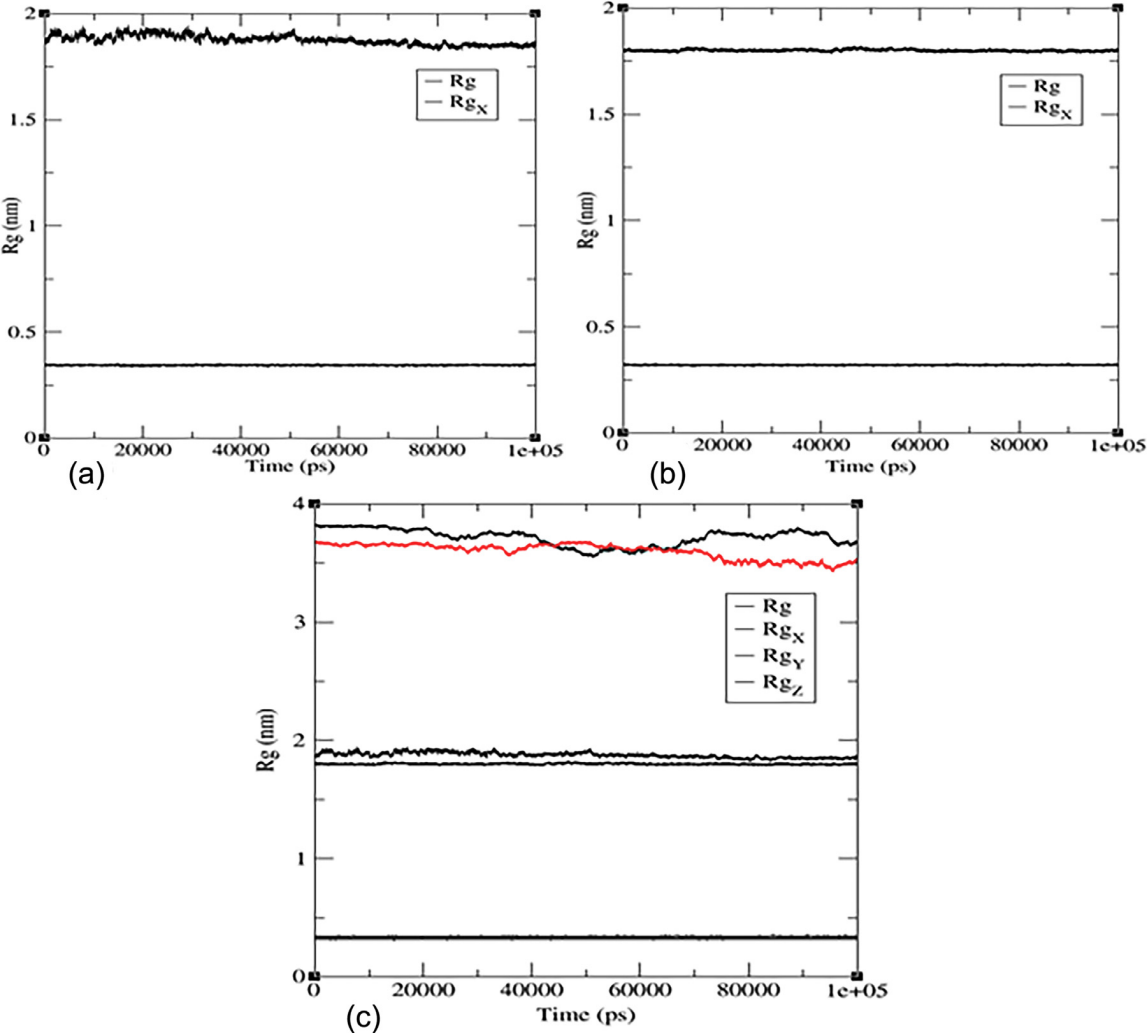
Plots for Radius of gyration, Rg (nm) versus time (ps) of the ligand (Rg), target protein (RgX), and protein‒ligand complex (RgY), for (a) flavonoid **7** with PDB:1P60, (b) flavonoid **7** with PDB: 1 × 2 J, (c) two proteins merged (1 × 2 J and 1P60) with ligand, water ion and ligand‒protein complex.

The radius of gyration (Rg) in molecular dynamics is used to monitor changes in the shape and compactness of a molecule over time or to compare the shapes of different molecules. The Rg values for the ligand‒protein complex between flavonoid (**7)** and (PDB:1P60) suggest an extended conformation, while the Rg values for the ligand alone suggest a compact conformation (see Fig. 7a). The Rg values for the ligand‒protein complex between flavonoid (**7)** and (PDB: 1 × 2 J) indicate a large and extended complex (see Fig. 7). Fig. 7a shows the radius of gyration for protein complex (PDB:1P60) with flavonoid (**7)**, and Fig. 7b shows the radius of gyration values of flavonoid 7 with (PDB:1 × 2 J) protein. Fig. 7c shows Rg values of two proteins merged (PDB:1 × 2 J and PDB:1P60) with ligand, water ion and ligand‒protein complex; in both lines, the straight line at 0.3 nm indicates water ions.

**Fig. 8:**
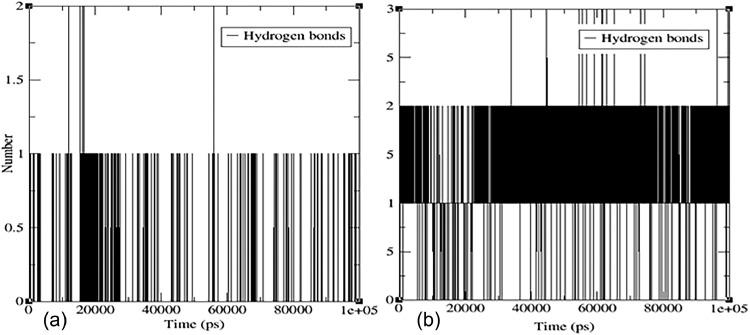
The number of hydrogen bonds versus time (ps) plots for hydrogen bond stabilization hydrogen bonding for (a) the protein complex of PDB: 1P60 and flavonoid **7** and (b) the protein complex of PDB: 1 × 2 J and flavonoid **7** during a 100 ns MD simulation.

According to the MD simulation results presented in (Fig. 8**)**, the number of hydrogen bonds formed between compound (**7)** and the active sites of (PDB: 1P60) and (PDB: 1 × 2 J) ranged from 0 to 2 during the 100 ns simulation period. This variability suggests that the interaction between the ligand and protein is dynamic and that the ligand can adopt different conformations and binding modes within the protein-binding pocket. The formation of stable hydrogen bonds indicates that flavonoid (**7)** has the potential to interact with both proteins' active sites.

**Fig. 9:**
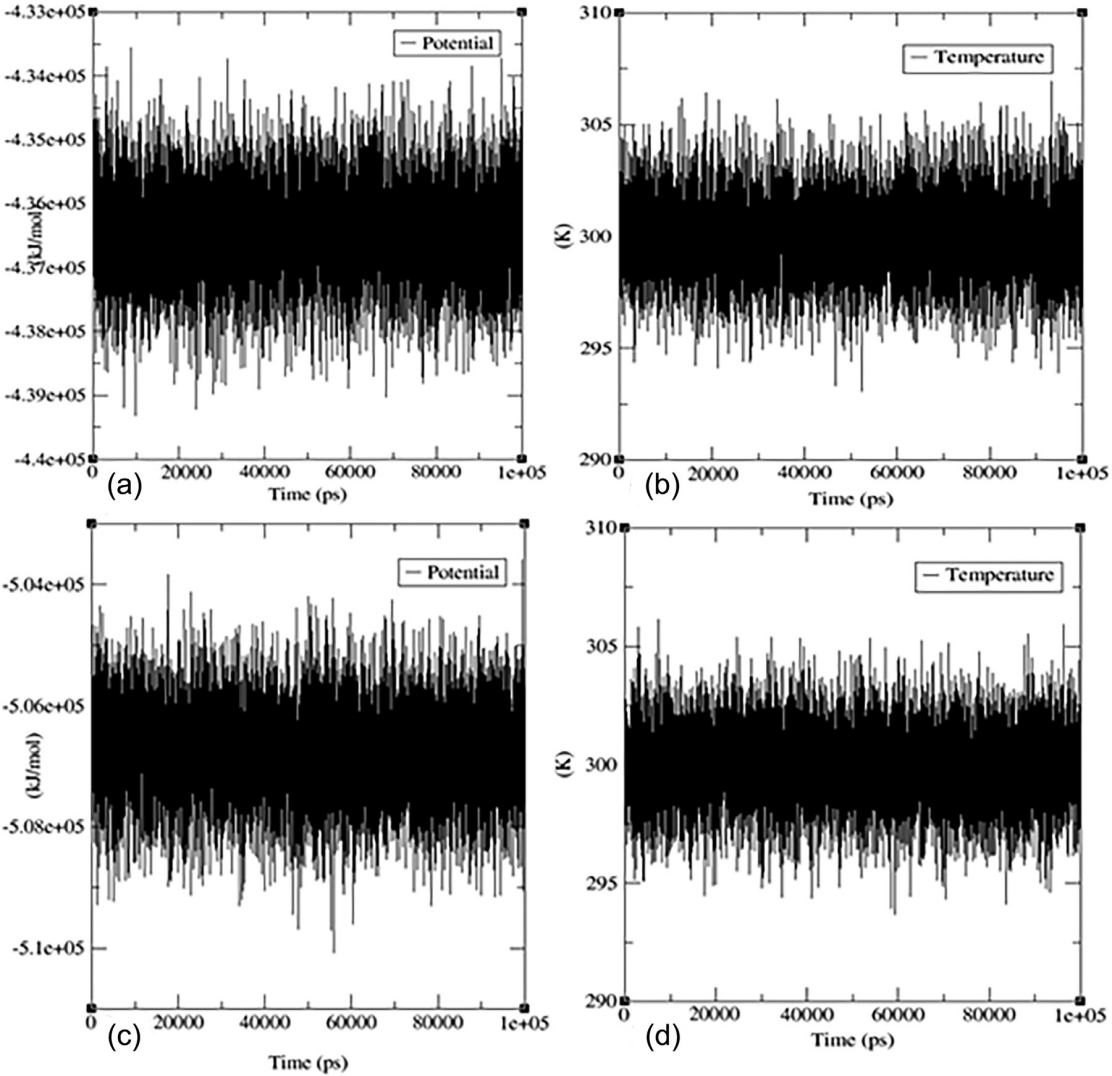
The temperature and potential energy curves over the course of the 100 ns MD simulation for (a) a potential energy graph for the protein‒ligand complex of PDB: 1 × 2 J and flavonoid **7** and (b) a temperature graph of the protein‒ligand complex of PDB: 1 × 2 J and flavonoid **7**. (c) Potential energy graph for the protein‒ligand complex of PDB: 1P60 and flavonoid **7**. (d) Temperature graph of the protein‒ligand complex of PDB: 1P60 and flavonoid **7.**

The temperature curves plotted in (Fig. 9**)** demonstrated stability, ranging from 298.5 to 301.5 Kelvin throughout the 3000 PS simulation. This suggests that the system maintained consistent thermal energy, despite the presence of the protein‒ligand complex. The potential energy of the system showed fluctuations ranging from −5.1e+05 to −5.04e+05 kJ mol ^– 1^, indicating changes in electrostatic interactions between atoms within the system.

The simulation results suggest that compound (**7)** has the potential to act as an inhibitor of both protein DCK and Nrf2, as it showed a stronger interaction with (PDB:1P60) and (PDB:1 × 2 J). This finding is promising, given that mRNA expression of DCK has been associated with poor prognosis in liver cancer patients. Therefore, flavonoid (**7)** could potentially be a therapeutic agent for liver diseases. However, further experimental studies are necessary to verify its efficacy and safety as a potential liver therapeutic.

**Fig. 10:**
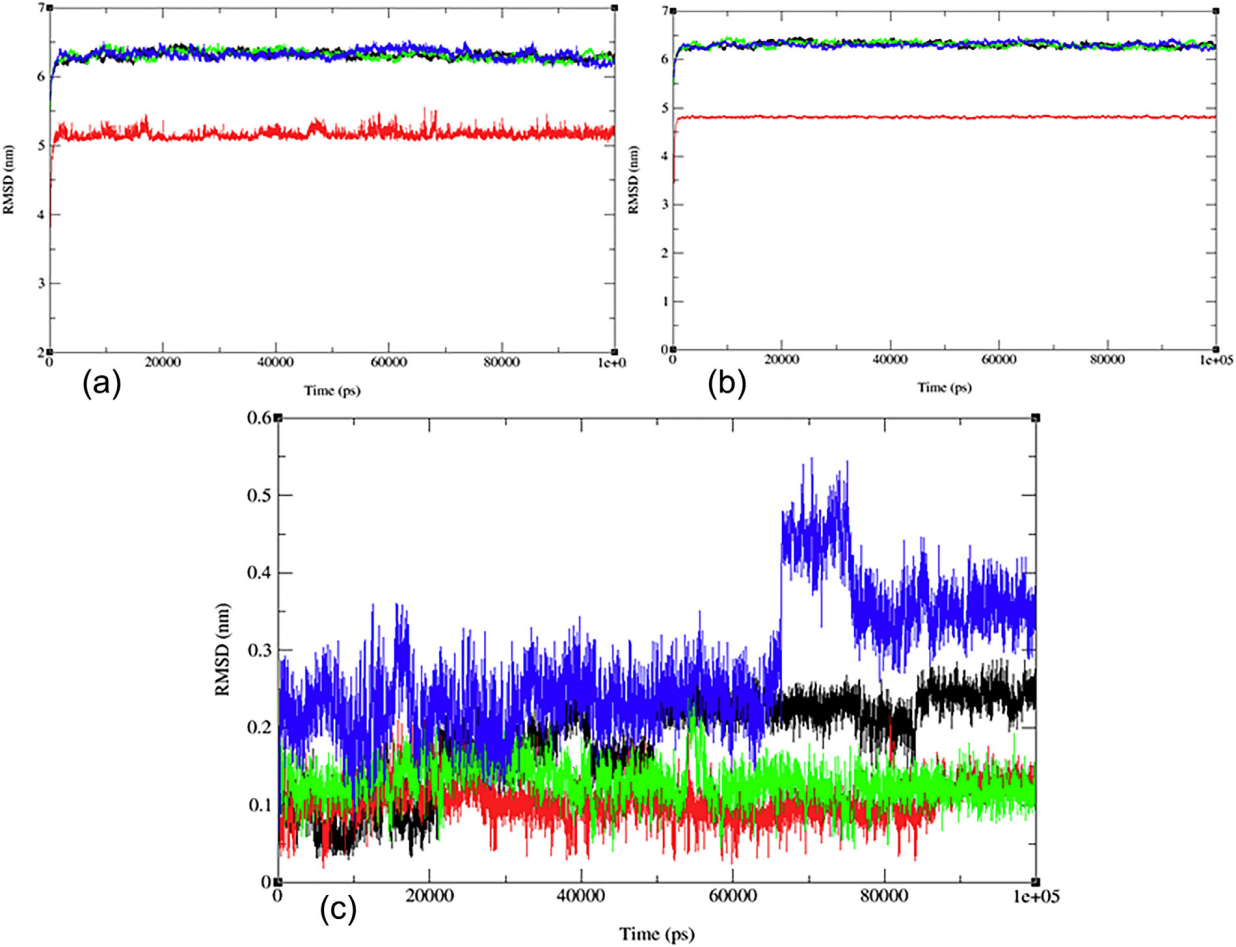
MD simulation evolution of RMSD for merged graphs a) ligand, b) protein, and c) merged docked protein‒ligand complexes between PDB protein: 1 × 2 J and modeled ligands **2** (blue line), **10** (red line), **14** (black line) and reference drug (Taxifolin) (green line) during 100 ns MD simulation.

To validate our findings and determine whether flavonoid (**7)** is optimal, we extended our investigation to include two additional flavonoids (**10** and **14**) and the standard drug, Taxifolin, which exhibited the most favorable docking results. Subsequently, we conducted molecular dynamics simulations, and the outcomes are presented in Fig. 10, comprising three panels: a) ligand comparison, b) protein comparisons, and c) protein-complex comparisons. Fig. 10 shows the root mean square deviation (RMSD) analysis for the three flavonoids and the standard drug. In Fig. 10c the red line corresponds to flavonoid **(7)**, while the blue, green, and black lines represent flavonoids (**10, 14)**, and the standard drug, respectively. Flavonoid **(7)** is the only one exhibiting an RMSD value close to 5, whereas the others collectively yield a value of 6.4. This notably higher combined value indicates that these ligands possess dissimilar shape orientations and exhibit less structural similarity. Notably, flavonoid (**7)** displays a lower RMSD value than the other compounds, as evident in Figs. 10a and 10b. This underscores its unique structural characteristics and suggests its potential as the most promising candidate. For more detailed information, including individual graphs for each flavonoid with respect to the reference molecule (PDB:1 × 2 J), please refer to the supplementary information (Figs. S17-S21).

**Fig. 11:**
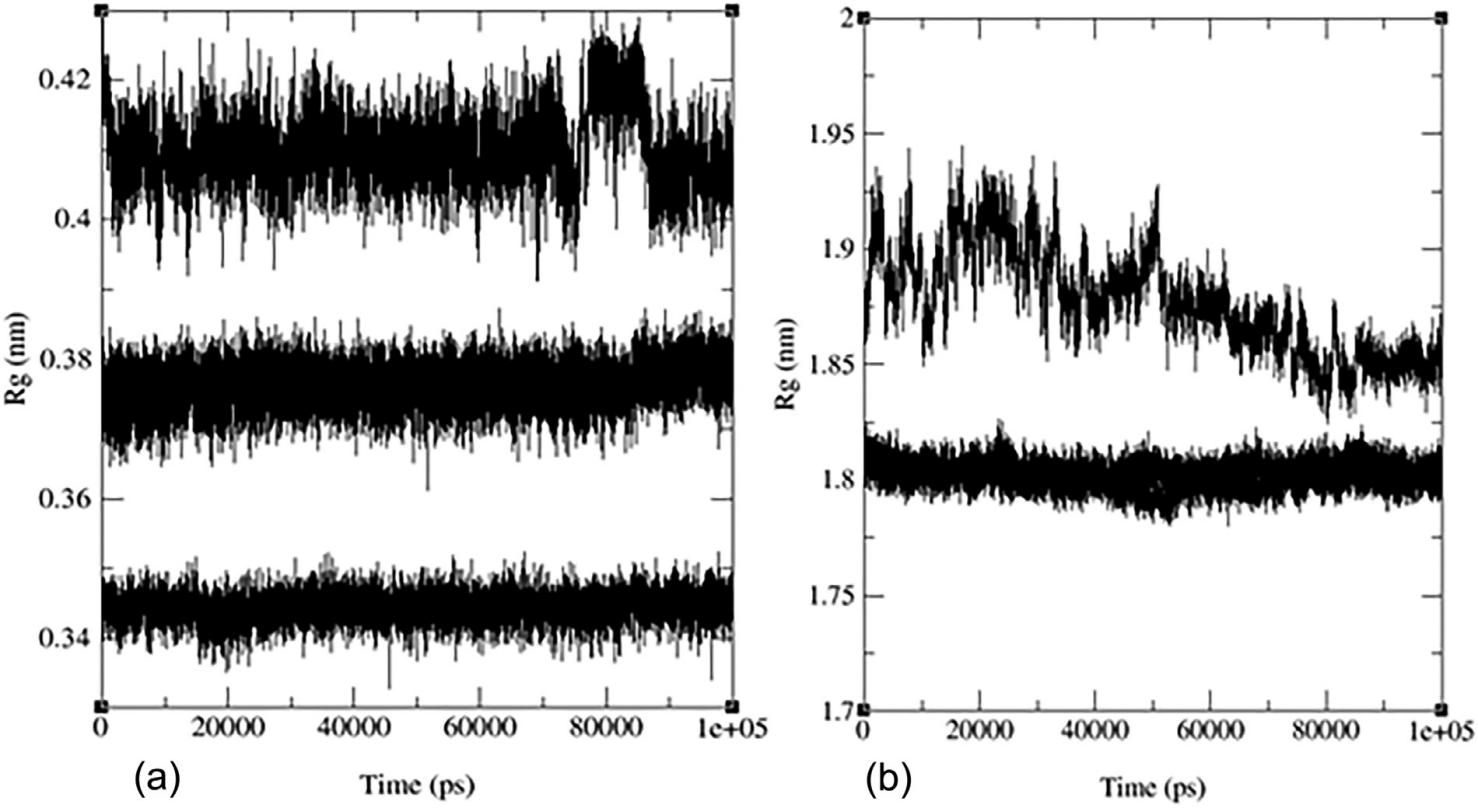
MD simulation evolution of Rg for merged graphs a) ligand and b) merged docked protein‒ligand complex between the PDB protein: 1 × 2 J and modeled ligands **2** (blue line), **10** (red line), **14** (black line) and reference drug (taxifolin) (green line) during 100 ns MD simulation.

Fig. 11 illustrates the merged radius of gyration for three flavonoids and the standard drug. In Fig. 11(a) we observe a radius of gyration range of 0.34–0.35 nm for flavonoid **7**, 0.37–0.38 nm for the standard drug, and 0.40–0.42 nm for flavonoids (**10** and **14)**. It is important to note that higher values in the radius of gyration indicate lower structural similarity and fewer interactions among molecules. Remarkably, flavonoid (**7)** exhibits the highest structural similarity and interactions among its molecules due to its lower radius of gyration. Detailed radius of gyration values and graphs for each flavonoid can be found in the supplementary information (see Figs. S22-S24).

**Fig. 12:**
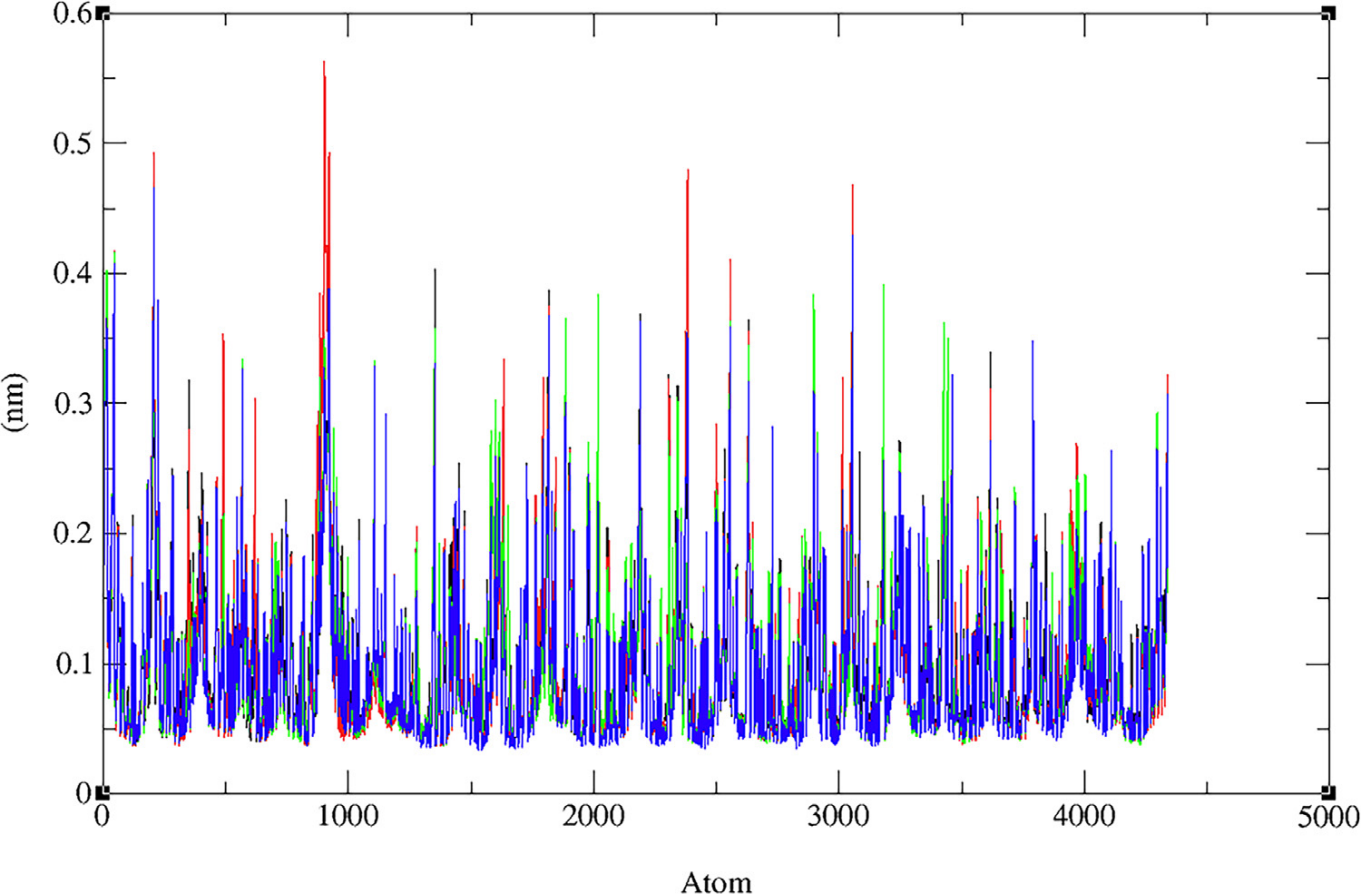
MD simulation evolution of RMSD for merged graphs of docked protein‒ligand complexes between PDB proteins: 1 × 2 J and modeled ligands **2** (blue line), **10** (red line), and **14** (black line) and the reference drug (taxifolin) (green line) during 100 ns of MD simulation.

Fig. 12 depicts the root mean square fluctuation (RMSF) values for four flavonoids, including the standard drug. RMSF serves as a metric for assessing the flexibility of a structure by evaluating the squared deviations from a reference structure. It is important to note that RMSF values are context-dependent and influenced by factors such as temperature, pressure, solvent conditions, and the choice of reference structure, which can vary between an average, crystallographic, or initial structure. These contextual and reference-related factors can lead to variations in RMSF values for the same structure, underscoring the need to consider these factors carefully. In general, an RMSF value within the range of 0.05 to 0.57 nm signifies varying degrees of flexibility, spanning from low to high, compared to other structures exposed to similar conditions. In our specific analysis, all RMSF values fall within this designated range. Notably, flavonoids (**7)** and (**14)** exhibit a peak at 0.57 nm, whereas flavonoid 10 demonstrates a peak at 0.54 nm, and the standard drug exhibits the lowest peak at 0.45 nm. They acknowledge that the interpretation of the RMSF values for flavonoids (**7)** and (**14)** hinges on their comparison with other structures within the same context, and the reference framework is crucial. For instance, the standard drug's RMSF value of 0.05 nm sets a benchmark for low flexibility. Consequently, structures with RMSF values ranging from 0.5 to 0.6 nm are highly flexible. For a more comprehensive understanding of the RMSF values for each protein complex, please refer to the supplementary information provided in Fig. S25**.**

**Fig. 13:**
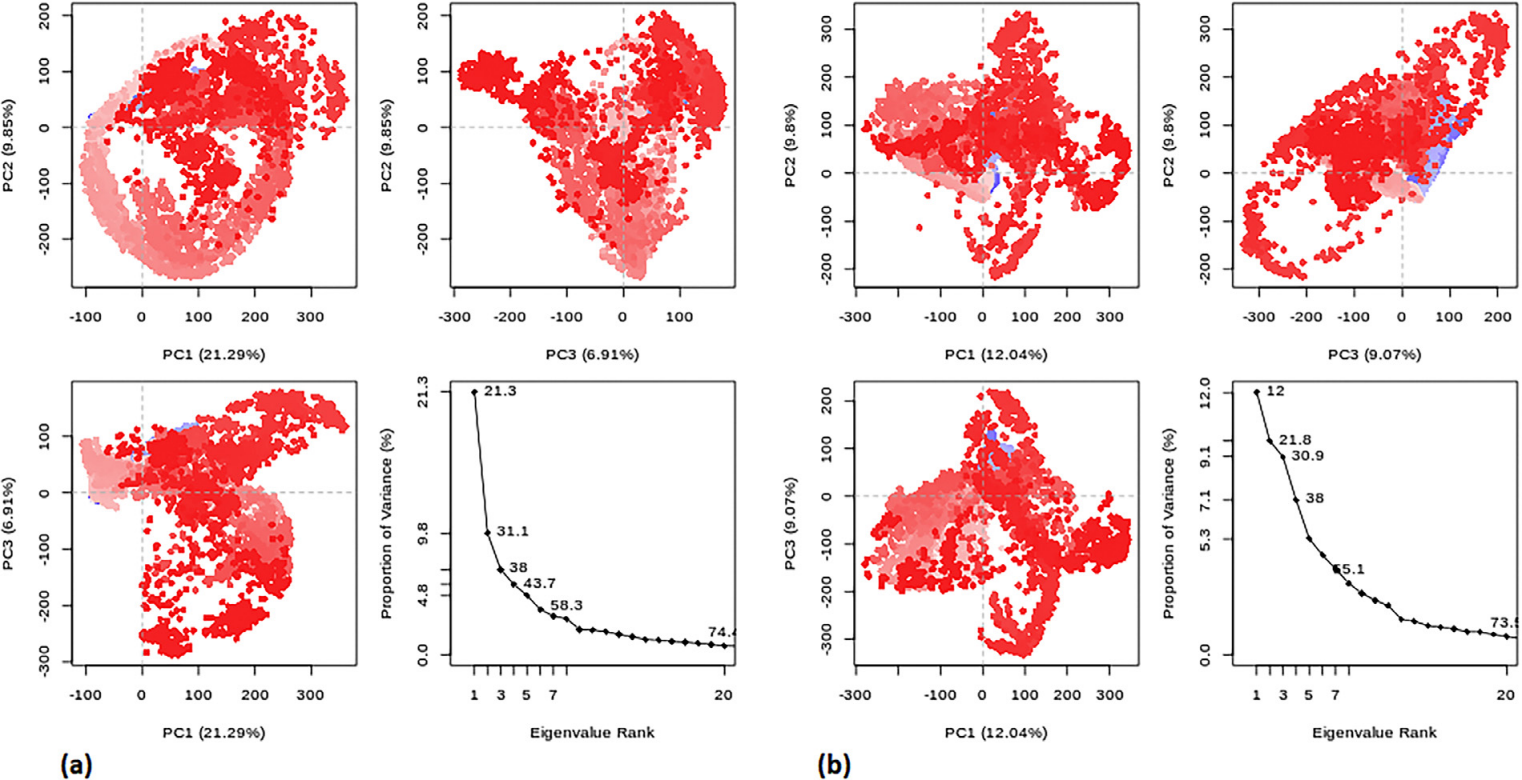
PCA conducted on the MD trajectory of the (a) 1P60 protein and (b) PDB:1 × 2 J protein at 300 K. In the plot, the blue dots correspond to energetically unstable conformational states, while the red dots indicate stable conformational states.

PCA was conducted on the MD trajectories of flavonoid (**7**) with protein (PDB:1P60) and the (PDB:1 × 2 J) protein‒ligand complex at 300 K using the Bio3D package, as shown in Fig. 13. The results in Fig. 13 show that PC1 captured the highest variability for (PDB:1P60) (21.29%) and (PDB:1 × 2 J) (12.04%), indicating significant internal motions within the MD trajectories. PC2 had lower variance percentages (6.91%) for 1P60 and (9.07%) for the (PDB:1 × 2 J) protein complex, while PC3 also explained 21.29% and 6.91% of the variance, respectively. Together, these three components explained a cumulative variance of 49.49% for the 1P60 complex and 33.15% for the (PDB:1 × 2 J) complex. These findings offer valuable insights into the collective motions and conformational changes occurring in protein‒ligand complexes during MD simulations. Additionally, the cosine content values of the eigenvectors calculated from the MD trajectory for the (PDB:1 × 2 J) and (PDB:1P60) complexes were determined to be 0.59 and 0.66, respectively. These values indicate that the simulations have reached convergence, demonstrating the stability and reliable sampling of the protein‒ligand complexes throughout the MD simulations.

**Fig. 14:**
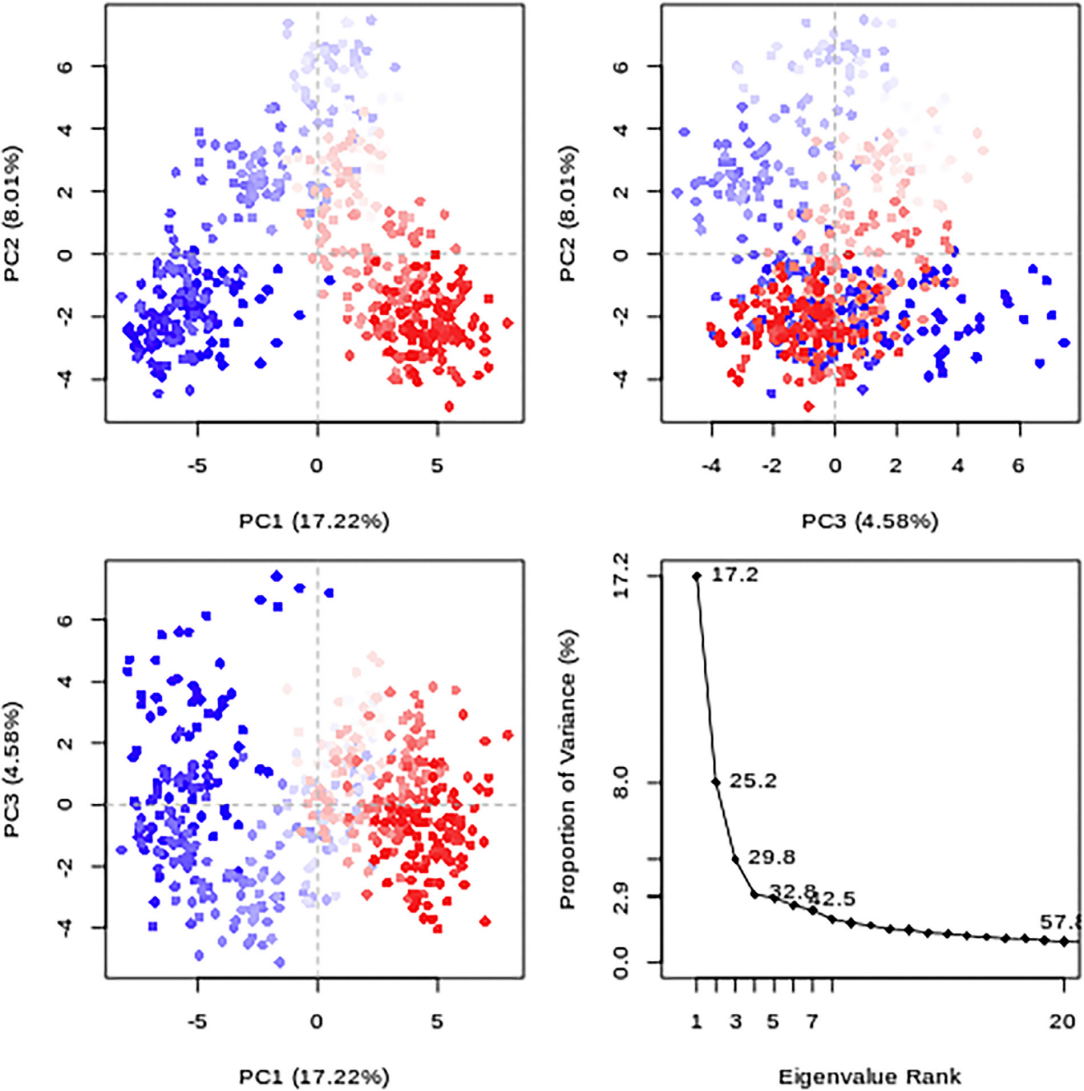
PCA value plot for flavonoids **10** and **14** conducted with the protein (PDB: 1 × 2 J).

Furthermore, PCA was conducted on flavonoids (**10** and **14)**, and the standard drug (taxifolin) in conjunction with the protein (PDB: 1 × 2 J) is shown in Fig. 14. This additional analysis was performed to validate our study's results and ascertain whether flavonoid (**7)** is a suitable candidate for liver drug development. It is worth noting that relying solely on docking results may not conclusively establish flavonoid (**7)** as the optimal choice. Consequently, flavonoids (**10** and **14)** and the standard drug were selected to examine their molecular dynamics trajectories. The cosine content for flavonoids (**10** and **14)** was calculated to be 0.01, while for the standard drug (taxifolin), it was 0.70. Cosine content values approaching 0 suggest that the molecular simulation explores diverse regions within the conformational space, indicating complex or irregular motion. However, it is crucial to recognize that these interpretations are not absolute and should be considered alongside other factors, including the simulation duration, system size, and choice of coordinates. Moreover, the cumulative variance of the three principal component analysis (PCA) components for flavonoids (**10)** and (**14)** in conjunction with protein (PDB:1 × 2 J) amounts to 39.02%. This finding implies that flavonoid (**7)** exhibits a lower cumulative variance than the others, indicating a more simplified or coarse-grained trajectory representation, as shown in Fig. 14.

## Conclusion

This study investigated analogs of flavones (**1**-**14**) to determine their efficacy as liver drugs. These derivatives (**1**-**14**) have been investigated computationally for their potential use against three cancer-related proteins and one parasite target. This work includes DFT calculations, molecular docking calculations, binding energy calculations, thermodynamic properties, HOMO and LUMO investigation, drug-likeness analyses, ADMET properties, MD simulation, and their biological activity spectra. Among all fifteen compounds, flavonoid (**7**) showed good agreement binding affinity as a liver therapeutic, as it has shown excellent efficiency through computational analysis. These results indicated that 5,7-dihydroxyflavone might be a promising drug molecule, as it showed good binding affinity compared to the standard drug 7,8-dihydroxyflavone with EGFR, HER2, FPPS, HGDS, and Keap1 on the Nrf2 protein. Moreover, flavonoids (**7**) obeyed the Lipinksi, Ghose, Veber, Egan, and Muegge rules. Flavonoid (**7)** showed an excellent intestinal absorption rate. However, validating all the results in the wet laboratory would be the best way to characterize the properties of these compounds as therapeutic liver drugs.

## CRediT authorship contribution statement

**Syeda Tasnim Quayum:** Formal analysis, Investigation, Methodology, Writing – original draft. **Nusrat Jahan Ikbal Esha:** Writing – review & editing. **Siam Siraji:** Writing – review & editing. **Sanaa S. Al Abbad:** Funding acquisition, Writing – review & editing. **Zainab H.A. Alsunaidi:** Writing – review & editing. **Mansour H. Almatarneh:** Writing – review & editing. **Shofiur Rahman:** Funding acquisition, Writing – review & editing. **Abdullah N. Alodhayb:** Funding acquisition, Writing – review & editing. **Khuloud A. Alibrahim:** Funding acquisition, Writing – review & editing. **Sarkar M.A. Kawsar:** Writing – review & editing. **Kabir M. Uddin:** Conceptualization, Data curation, Funding acquisition, Resources, Supervision, Validation, Writing – original draft, Writing – review & editing.

## Declaration of Competing Interest

The author declares that they have no known competing financial interests or personal relationships that could have appeared to influence the work reported in this paper.

## Data Availability

Data will be made available on request.

## Appendix Supplementary materials

Image, application 1
