# Supplementary material for: Exploring the effectiveness of flavone derivatives for treating liver diseases: Utilizing DFT, molecular docking, and molecular dynamics techniques
Source: MethodsX. 2023 Dec 29;12:102537. doi: 10.1016/j.mex.2023.102537 (PMC10828815; doi:10.1016/j.mex.2023.102537)
Supplement: Supplementary file 1 [file mmc1.docx]

**Exploring the Effectiveness of Flavone Derivatives for Treating Liver Diseases: Utilizing DFT, Molecular Docking, and**

**Molecular Dynamics Techniques**

**Syeda Tasnim Quayum,^1^ Nusrat Jahan Ikbal Esha,^1^ Siam Siraji,^1^ Sanaa S. Al Abbad,^2^ Zainab H. A. Alsunaidi,^2^ Mansour H. Almatarneh,^3^ Shofiur Rahman,^4^ Khuloud A. Alibrahim,^5^ Sarkar M. A. Kawsar^6^ and Kabir M. Uddin^1,^***

^1^Department of Biochemistry and Microbiology, North South University, Bashundhara, Dhaka-1217, Bangladesh

^2^Department of Chemistry, Imam Abdulrahman Bin Faisal University

Dammam 31441, Saudi Arabia

^3^Department of Chemistry, University of Jordan

Amman 11942, Jordan

^4^Biological and Environmental Sensing Research Unit, King Abdullah Institute for Nanotechnology, King Saud University,

Riyadh 11451, Saudi Arabia

^5^Department of Chemistry, Princess Nora bint Abdulrahman University, College of Science, Riyadh, Al Riyadh, 11671, Saudi Arabia

^6^Lab of Carbohydrate and Nucleoside Chemistry, Department of Chemistry, University of Chittagong, Chittagong-4331, Bangladesh

**Orcid ID:**

- Syeda Tasnim Quayum: <https://orcid.org/0000-0003-3615-8101>
- Nusrat Jahan Ikbal Esha: <https://orcid.org/0009-0007-7566-6543>
- Dr. Shofiur Rahman: <https://orcid.org/0000-0003-4219-4758>
- Dr. Sarkar M. A. Kawsar: <https://orcid.org/0000-0001-7964-9117>
- Dr. Kabir M. Uddin: <https://orcid.org/0000-0002-5518-2345>

Tel.: +8801796585904

Fax: +8802-55668202

E-mail: [mohammed.uddin11@northsouth.edu](mailto:mohammed.uddin11@northsouth.edu)

[kabirmuddin@gmail.com](mailto:kabirmuddin@gmail.com)

**Table S1**

Optimized structure for Flavone (**1**) and cartesian Z-matrix.

| **Flavone (1)** | | | **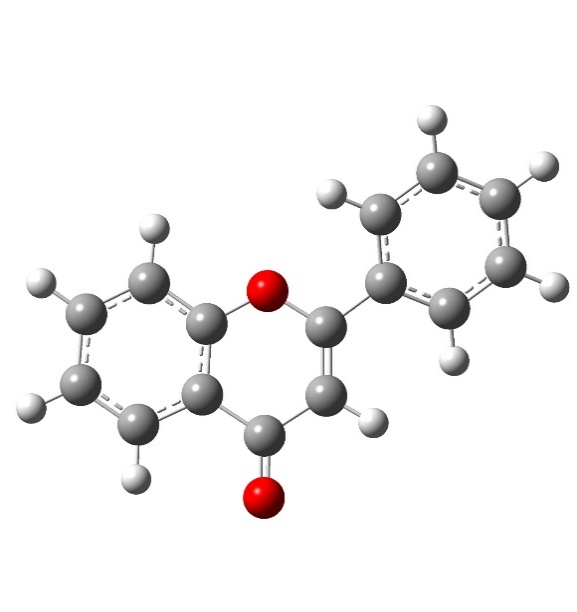** | | |
| --- | --- | --- | --- | --- | --- |
| Center  Number | Atom | Standard orientation: Coordinates (Angstroms) | | | |
|  |  | X | | Y | Z |
| 1 | O | 4.322750 | | -2.368276 | 0.145817 |
| 2 | O | -0.035428 | | -0.619757 | 0.050774 |
| 3 | O | 1.327805 | | 3.234587 | -0.178228 |
| 4 | C | 1.838161 | | 0.911901 | -0.039327 |
| 5 | C | 1.313486 | | -0.385316 | 0.032280 |
| 6 | C | -0.913669 | | 0.426834 | -0.015938 |
| 7 | C | -2.316649 | | -0.027476 | 0.003629 |
| 8 | C | 0.924572 | | 2.073796 | -0.110927 |
| 9 | C | -0.488677 | | 1.712894 | -0.104766 |
| 10 | C | 3.233203 | | 1.072021 | -0.048635 |
| 11 | C | 2.139141 | | -1.511171 | 0.096698 |
| 12 | C | -3.352168 | | 0.856040 | 0.353108 |
| 13 | C | -2.640852 | | -1.352959 | -0.331068 |
| 14 | C | 4.077997 | | -0.024216 | 0.013264 |
| 15 | C | 3.507289 | | -1.299709 | 0.085043 |
| 16 | C | -4.676432 | | 0.428285 | 0.350832 |
| 17 | C | -3.967961 | | -1.776187 | -0.331542 |
| 18 | C | -4.990168 | | -0.888440 | 0.006459 |
| 19 | H | -1.206316 | | 2.518142 | -0.192832 |
| 20 | H | 3.618508 | | 2.084430 | -0.106287 |
| 21 | H | 1.724272 | | -2.509849 | 0.154999 |
| 22 | H | -3.119797 | | 1.873419 | 0.648897 |
| 23 | H | -1.849819 | | -2.044015 | -0.597008 |
| 24 | H | 5.157236 | | 0.076673 | 0.007753 |
| 25 | H | -5.464287 | | 1.121910 | 0.628132 |
| 26 | H | -4.203316 | | -2.802003 | -0.598517 |
| 27 | H | -6.023901 | | -1.220642 | 0.007472 |

**Table S2**

Optimized structure for 5-hydroxyflavone (**2**) and cartesian Z-matrix.

| **5-hydroxyflavone (2)** | | | 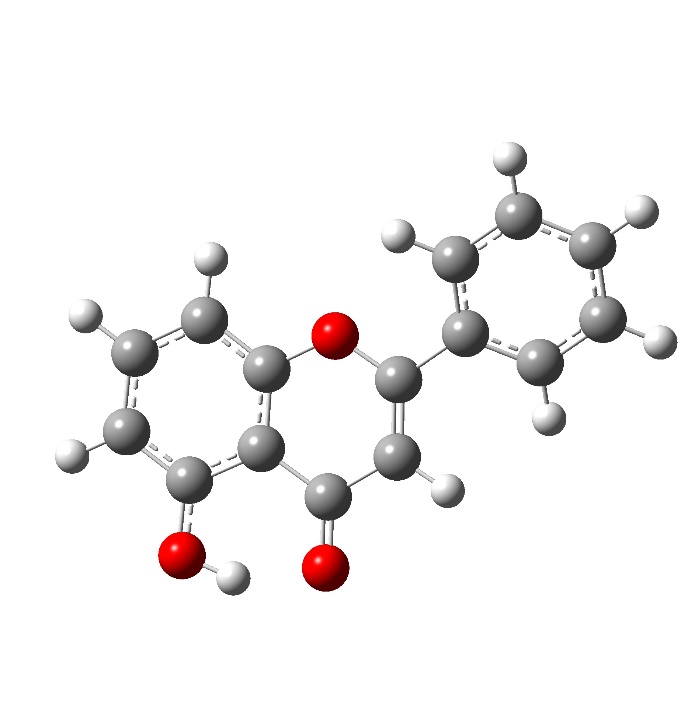 | | |
| --- | --- | --- | --- | --- | --- |
| Center  Number | Atom | Standard orientation: Coordinates (Angstroms) | | | |
|  |  | X | | Y | Z |
| 1 | O | 0.163958 | | -0.912721 | 0.075576 |
| 2 | O | -4.105479 | | 1.293679 | -0.083237 |
| 3 | O | -1.878187 | | 2.618958 | -0.170821 |
| 4 | C | -1.948439 | | 0.252709 | -0.008708 |
| 5 | C | -1.210235 | | -0.939184 | 0.068223 |
| 6 | C | 0.837526 | | 0.266152 | -0.006101 |
| 7 | C | 2.299906 | | 0.076594 | 0.00316 |
| 8 | C | -1.258381 | | 1.534935 | -0.096008 |
| 9 | C | 0.187276 | | 1.457581 | -0.100199 |
| 10 | C | -3.368806 | | 0.179411 | -0.008528 |
| 11 | C | -1.821192 | | -2.186689 | 0.147534 |
| 12 | C | -3.991821 | | -1.070639 | 0.070438 |
| 13 | C | -3.216385 | | -2.227714 | 0.146802 |
| 14 | C | 3.160224 | | 1.13997 | 0.325625 |
| 15 | C | 2.855932 | | -1.17413 | -0.314038 |
| 16 | C | 4.540059 | | 0.959481 | 0.314986 |
| 17 | C | 4.237628 | | -1.34932 | -0.323448 |
| 18 | C | 5.084317 | | -0.284612 | -0.011782 |
| 19 | H | 0.740437 | | 2.381707 | -0.201025 |
| 20 | H | -1.216901 | | -3.08377 | 0.2089 |
| 21 | H | -5.074986 | | -1.11429 | 0.070685 |
| 22 | H | -3.713313 | | -3.191356 | 0.208259 |
| 23 | H | 2.750258 | | 2.103787 | 0.607563 |
| 24 | H | 2.200534 | | -2.001408 | -0.559037 |
| 25 | H | 5.191368 | | 1.789303 | 0.571531 |
| 26 | H | 4.653211 | | -2.319913 | -0.576593 |
| 27 | H | 6.161159 | | -0.423607 | -0.017745 |
| 28 | H | -3.459757 | | 2.052945 | -0.13242 |

**Table S3**

Optimized structure for 6-hydroxyflavone (**3**) and cartesian Z-matrix.

| **6-hydroxyflavone** (**3**) | | | 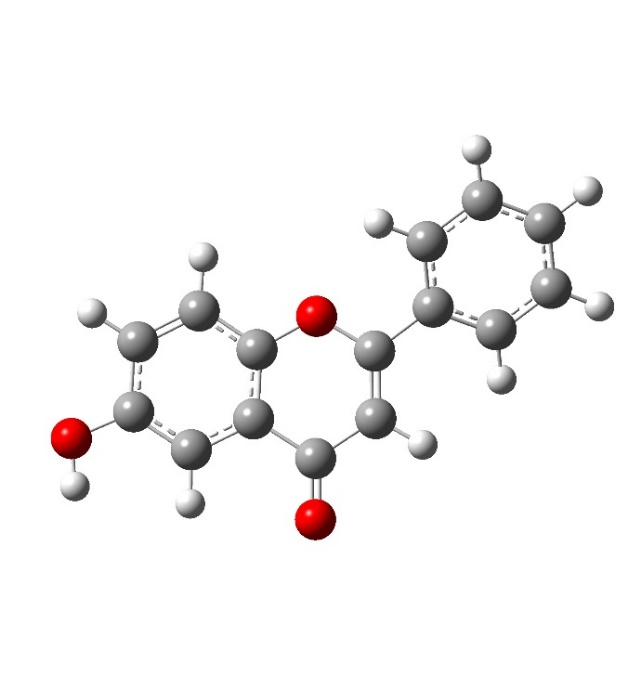 | | |
| --- | --- | --- | --- | --- | --- |
| Center  Number | Atom | Standard orientation: Coordinates (Angstroms) | | | |
|  |  | X | | Y | Z |
| 1 | O | 0.185017 | | -0.775437 | 0.071728 |
| 2 | O | -1.524438 | | 2.937087 | -0.206784 |
| 3 | O | -5.329094 | | -0.567781 | 0.044682 |
| 4 | C | -1.822569 | | 0.580535 | -0.031533 |
| 5 | C | -1.184506 | | -0.66003 | 0.055973 |
| 6 | C | 0.961208 | | 0.340129 | -0.01357 |
| 7 | C | 2.401326 | | 0.018812 | 0.004099 |
| 8 | C | -1.014442 | | 1.818809 | -0.123971 |
| 9 | C | 0.42214 | | 1.584347 | -0.118302 |
| 10 | C | -3.223652 | | 0.630048 | -0.037556 |
| 11 | C | -1.922532 | | -1.845868 | 0.140412 |
| 12 | C | -3.964132 | | -0.541747 | 0.04472 |
| 13 | C | -3.306259 | | -1.784497 | 0.13411 |
| 14 | C | 3.352948 | | 0.998388 | 0.334947 |
| 15 | C | 2.844913 | | -1.275956 | -0.313397 |
| 16 | C | 4.711067 | | 0.694025 | 0.331545 |
| 17 | C | 4.205202 | | -1.575772 | -0.315038 |
| 18 | C | 5.143189 | | -0.593161 | 0.004501 |
| 19 | H | 1.065516 | | 2.448449 | -0.220976 |
| 20 | H | -3.690226 | | 1.609078 | -0.108486 |
| 21 | H | -1.401274 | | -2.794414 | 0.210392 |
| 22 | H | -3.904594 | | -2.68687 | 0.198097 |
| 23 | H | 3.029454 | | 1.994516 | 0.617096 |
| 24 | H | 2.118959 | | -2.040047 | -0.564885 |
| 25 | H | 5.432998 | | 1.46123 | 0.594323 |
| 26 | H | 4.532622 | | -2.579553 | -0.568657 |
| 27 | H | 6.203105 | | -0.829005 | 0.004419 |

**Table S4**

Optimized structure for 7-hydroxyflavone (**4**) and cartesian Z-matrix.

| **7-hydroxyflavone (4)** | | | 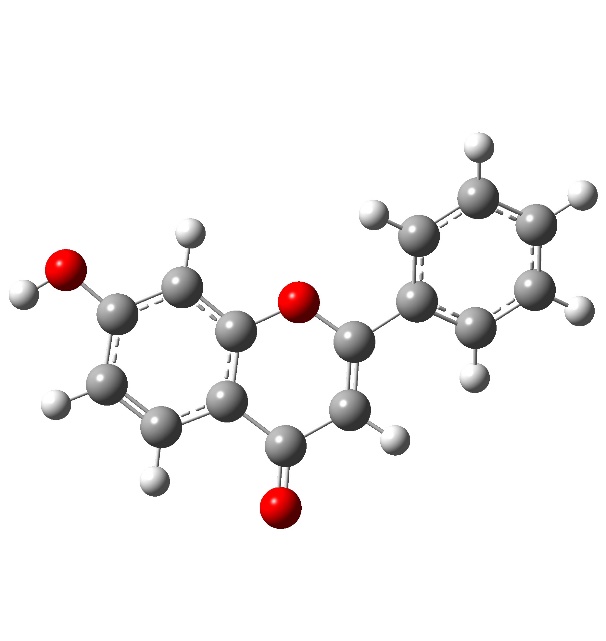 | | |
| --- | --- | --- | --- | --- | --- |
| Center  Number | Atom | Standard orientation: Coordinates (Angstroms) | | | |
|  |  | X | | Y | Z |
| 1 | O | 0.037663 | | -0.614386 | 0.050783 |
| 2 | O | -1.305774 | | 3.24857 | -0.17543 |
| 3 | O | -4.299815 | | -2.41862 | 0.145241 |
| 4 | C | -1.826135 | | 0.927895 | -0.039013 |
| 5 | C | -1.312329 | | -0.37498 | 0.031801 |
| 6 | C | 0.91972 | | 0.428091 | -0.01473 |
| 7 | C | 2.321158 | | -0.032923 | 0.004199 |
| 8 | C | -0.910608 | | 2.083781 | -0.109037 |
| 9 | C | 0.502054 | | 1.71635 | -0.102499 |
| 10 | C | -3.22017 | | 1.08712 | -0.048495 |
| 11 | C | -2.138507 | | -1.495946 | 0.094467 |
| 12 | C | -3.518592 | | -1.305741 | 0.083659 |
| 13 | C | -4.063725 | | -0.008176 | 0.011852 |
| 14 | C | 3.362137 | | 0.845681 | 0.349652 |
| 15 | C | 2.638276 | | -1.360961 | -0.326985 |
| 16 | C | 4.68423 | | 0.411061 | 0.346563 |
| 17 | C | 3.963194 | | -1.791305 | -0.328145 |
| 18 | C | 4.990792 | | -0.908239 | 0.0056 |
| 19 | H | 1.223613 | | 2.51827 | -0.189342 |
| 20 | H | -3.607084 | | 2.099141 | -0.105195 |
| 21 | H | -1.718812 | | -2.49292 | 0.151851 |
| 22 | H | -5.143086 | | 0.124289 | 0.004245 |
| 23 | H | 3.135528 | | 1.865165 | 0.642675 |
| 24 | H | 1.842791 | | -2.048349 | -0.589053 |
| 25 | H | 5.4761 | | 1.101492 | 0.62064 |
| 26 | H | 4.192543 | | -2.819289 | -0.592265 |
| 27 | H | 6.022806 | | -1.245896 | 0.006072 |
| 28 | H | -5.229959 | | -2.156665 | 0.132283 |

**Table S5**

Optimized structure for 3,5-dihydroxyflavone (**5**) and cartesian Z-matrix.

| **3,5-dihydroxyflavone (5)** | | | **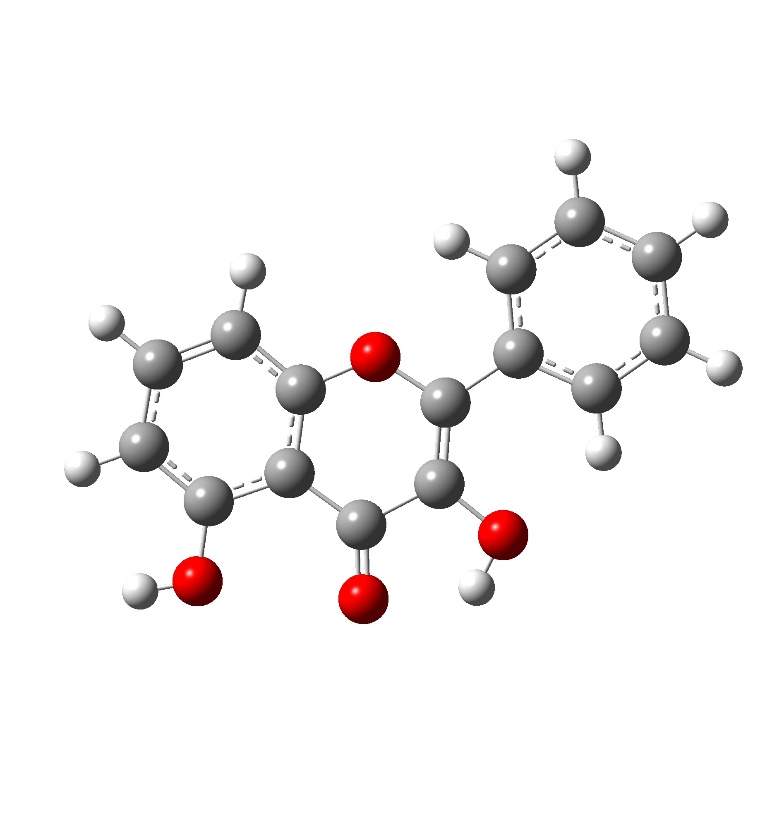** | | |
| --- | --- | --- | --- | --- | --- |
| Center  Number | Atom | Standard orientation: Coordinates (Angstroms) | | | |
|  |  | X | | Y | Z |
| 1 | O | 1.019275 | | -0.081883 | 0 |
| 2 | O | -2.375016 | | 1.195251 | 0 |
| 3 | O | -1.877145 | | -3.903005 | 0 |
| 4 | O | -2.838068 | | -1.321296 | 0 |
| 5 | C | -0.505138 | | -1.960891 | 0 |
| 6 | C | 0.797356 | | -1.420387 | 0 |
| 7 | C | 0 | | 0.844014 | 0 |
| 8 | C | -1.630649 | | -1.035477 | 0 |
| 9 | C | -1.288329 | | 0.39382 | 0 |
| 10 | C | 0.494938 | | 2.225819 | 0 |
| 11 | C | -0.62995 | | -3.378233 | 0 |
| 12 | C | 1.943303 | | -2.224339 | 0 |
| 13 | C | 0.508635 | | -4.180559 | 0 |
| 14 | C | 1.782639 | | -3.600114 | 0 |
| 15 | C | -0.385533 | | 3.326573 | 0 |
| 16 | C | 1.881915 | | 2.474912 | 0 |
| 17 | C | 0.114281 | | 4.626364 | 0 |
| 18 | C | 2.369796 | | 3.777935 | 0 |
| 19 | C | 1.489802 | | 4.861596 | 0 |
| 20 | H | 2.919846 | | -1.755191 | 0 |
| 21 | H | 0.401143 | | -5.262292 | 0 |
| 22 | H | 2.657781 | | -4.242461 | 0 |
| 23 | H | -1.453502 | | 3.158684 | 0 |
| 24 | H | 2.573481 | | 1.641731 | 0 |
| 25 | H | -0.580059 | | 5.461499 | 0 |
| 26 | H | 3.442748 | | 3.946646 | 0 |
| 27 | H | 1.872297 | | 5.877998 | 0 |
| 28 | H | -3.115966 | | 0.542402 | 0 |
| 29 | H | -1.808533 | | -4.867761 | 0 |

**Table S6**

Optimized structure for 3,6-dihydroxyflavone (**6**) and cartesian Z-matrix.

| **3,6-dihydroxyflavone (6)** | | | **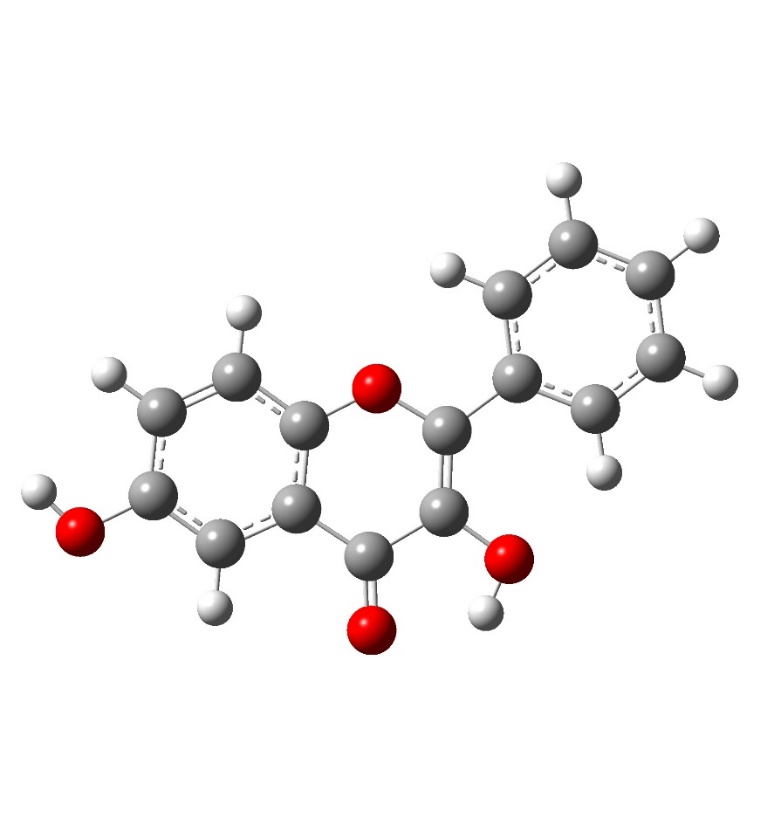** | | |
| --- | --- | --- | --- | --- | --- |
| Center  Number | Atom | Standard orientation: Coordinates (Angstroms) | | | |
|  |  | X | | Y | Z |
| 1 | O | 0 | | -0.90744 | 0 |
| 2 | O | 1.362366 | | 2.461251 | 0 |
| 3 | O | -1.17949 | | 2.978412 | 0 |
| 4 | O | -5.43453 | | 0.049227 | 0 |
| 5 | C | -1.83914 | | 0.668638 | 0 |
| 6 | C | -1.33525 | | -0.64105 | 0 |
| 7 | C | 0.94174 | | 0.092771 | 0 |
| 8 | C | 0.524969 | | 1.398317 | 0 |
| 9 | C | -0.88673 | | 1.770894 | 0 |
| 10 | C | 2.314218 | | -0.42973 | 0 |
| 11 | C | -3.22654 | | 0.882625 | 0 |
| 12 | C | -2.20583 | | -1.73647 | 0 |
| 13 | C | -4.09227 | | -0.20133 | 0 |
| 14 | C | -3.57354 | | -1.51343 | 0 |
| 15 | C | 2.533486 | | -1.82206 | 0 |
| 16 | C | 3.434186 | | 0.42628 | 0 |
| 17 | C | 3.825365 | | -2.33832 | 0 |
| 18 | C | 4.722845 | | -0.10182 | 0 |
| 19 | C | 4.927985 | | -1.48205 | 0 |
| 20 | H | -3.60507 | | 1.898076 | 0 |
| 21 | H | -1.79874 | | -2.74153 | 0 |
| 22 | H | -4.2542 | | -2.36178 | 0 |
| 23 | H | 1.685616 | | -2.49545 | 0 |
| 24 | H | 3.290838 | | 1.497625 | 0 |
| 25 | H | 3.970773 | | -3.41465 | 0 |
| 26 | H | 5.572691 | | 0.574376 | 0 |
| 27 | H | 5.935733 | | -1.88678 | 0 |
| 28 | H | 0.741477 | | 3.225379 | 0 |
| 29 | H | -5.91888 | | -0.78645 | 0 |

**Table S7**

Optimized structure for 5,7-dihydroxyflavone (**7**) and cartesian Z-matrix.

| **5,7-dihydroxyflavone (7)** | | | **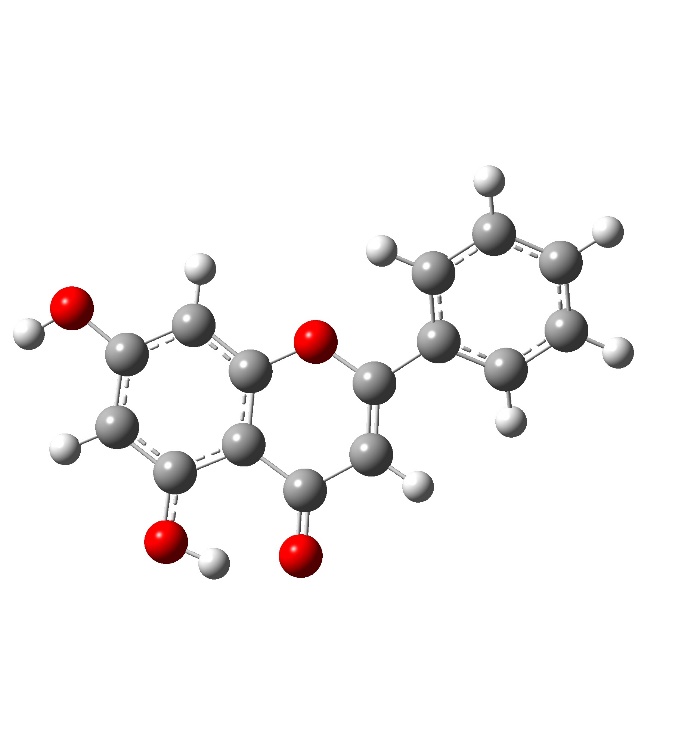** | | |
| --- | --- | --- | --- | --- | --- |
| Center  Number | Atom | Standard orientation: Coordinates (Angstroms) | | | |
|  |  | X | | Y | Z |
| 1 | O | -0.317989 | | -0.741977 | -0.052687 |
| 2 | O | 3.672047 | | 1.923557 | 0.088164 |
| 3 | O | 1.319951 | | 2.9993 | 0.145231 |
| 4 | O | 3.884051 | | -2.840862 | -0.144135 |
| 5 | C | 1.644649 | | 0.655191 | 0.021654 |
| 6 | C | 1.048887 | | -0.615982 | -0.039141 |
| 7 | C | -1.119863 | | 0.358491 | 0.007516 |
| 8 | C | 0.823114 | | 1.850779 | 0.085243 |
| 9 | C | -2.551384 | | 0.005258 | -0.004494 |
| 10 | C | 3.065565 | | 0.734249 | 0.028342 |
| 11 | C | -0.607073 | | 1.61514 | 0.084945 |
| 12 | C | 1.787008 | | -1.789326 | -0.095529 |
| 13 | C | 3.182648 | | -1.677663 | -0.08843 |
| 14 | C | 3.82516 | | -0.434241 | -0.027385 |
| 15 | C | -3.523909 | | 0.9601 | -0.347376 |
| 16 | C | -2.965454 | | -1.294898 | 0.330565 |
| 17 | C | -4.874842 | | 0.626244 | -0.338514 |
| 18 | C | -4.318898 | | -1.6238 | 0.337623 |
| 19 | C | -5.278006 | | -0.665845 | 0.006167 |
| 20 | H | -1.259808 | | 2.47371 | 0.168488 |
| 21 | H | 1.307041 | | -2.757872 | -0.145707 |
| 22 | H | 4.908236 | | -0.357938 | -0.021942 |
| 23 | H | -3.222746 | | 1.959181 | -0.643492 |
| 24 | H | -2.222753 | | -2.039651 | 0.590683 |
| 25 | H | -5.613724 | | 1.373739 | -0.610523 |
| 26 | H | -4.62428 | | -2.63098 | 0.604492 |
| 27 | H | -6.332503 | | -0.924667 | 0.010624 |
| 28 | H | 2.940261 | | 2.605407 | 0.122552 |
| 29 | H | 4.830177 | | -2.643261 | -0.134891 |

**Table S8**

Optimized structure for 3,5-dichloroflavone (**8**)and cartesian Z-matrix.

| **3,5-dichloroflavone (8)** | | | **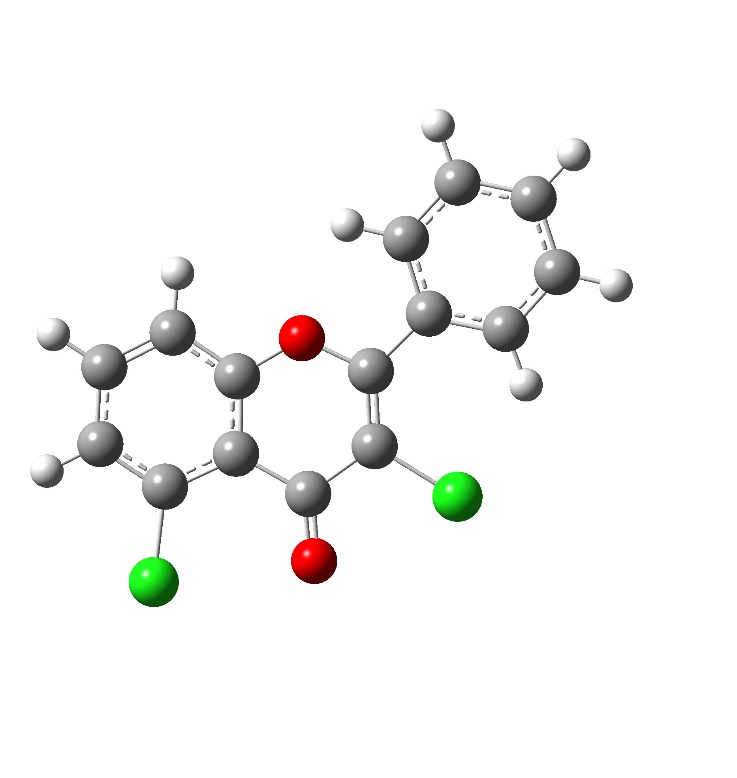** | | |
| --- | --- | --- | --- | --- | --- |
| Center  Number | Atom | Standard orientation: Coordinates (Angstroms) | | | |
|  |  | X | | Y | Z |
| 1 | Cl | 2.750966 | | 1.037046 | 0 |
| 2 | Cl | 0.980945 | | -4.376784 | 0 |
| 3 | O | -1.181465 | | 0.345843 | 0 |
| 4 | O | 2.273613 | | -1.765171 | 0 |
| 5 | C | -0.116482 | | -1.826621 | 0 |
| 6 | C | -1.262229 | | -1.013966 | 0 |
| 7 | C | 0 | | 1.039201 | 0 |
| 8 | C | -0.277005 | | 2.496295 | 0 |
| 9 | C | 1.210041 | | -1.163504 | 0 |
| 10 | C | 1.16348 | | 0.320594 | 0 |
| 11 | C | -0.334009 | | -3.22676 | 0 |
| 12 | C | -2.562042 | | -1.527345 | 0 |
| 13 | C | -1.620442 | | -3.753567 | 0 |
| 14 | C | -1.623314 | | 2.922985 | 0 |
| 15 | C | 0.72335 | | 3.488328 | 0 |
| 16 | C | -2.730206 | | -2.901809 | 0 |
| 17 | C | -1.950091 | | 4.274538 | 0 |
| 18 | C | 0.38711 | | 4.840165 | 0 |
| 19 | C | -0.946671 | | 5.243867 | 0 |
| 20 | H | -3.40142 | | -0.841687 | 0 |
| 21 | H | -1.749885 | | -4.829265 | 0 |
| 22 | H | -2.417541 | | 2.189482 | 0 |
| 23 | H | 1.76537 | | 3.213481 | 0 |
| 24 | H | -3.729512 | | -3.325106 | 0 |
| 25 | H | -2.995118 | | 4.569303 | 0 |
| 26 | H | 1.181426 | | 5.5803 | 0 |
| 27 | H | -1.201929 | | 6.299264 | 0 |

**Table S9**

Optimized structure for 6-bromo-4^’^-chloroflavone (**9**) and cartesian Z-matrix.

| **6-bromo-4^’^-chloroflavone (9)** | | | **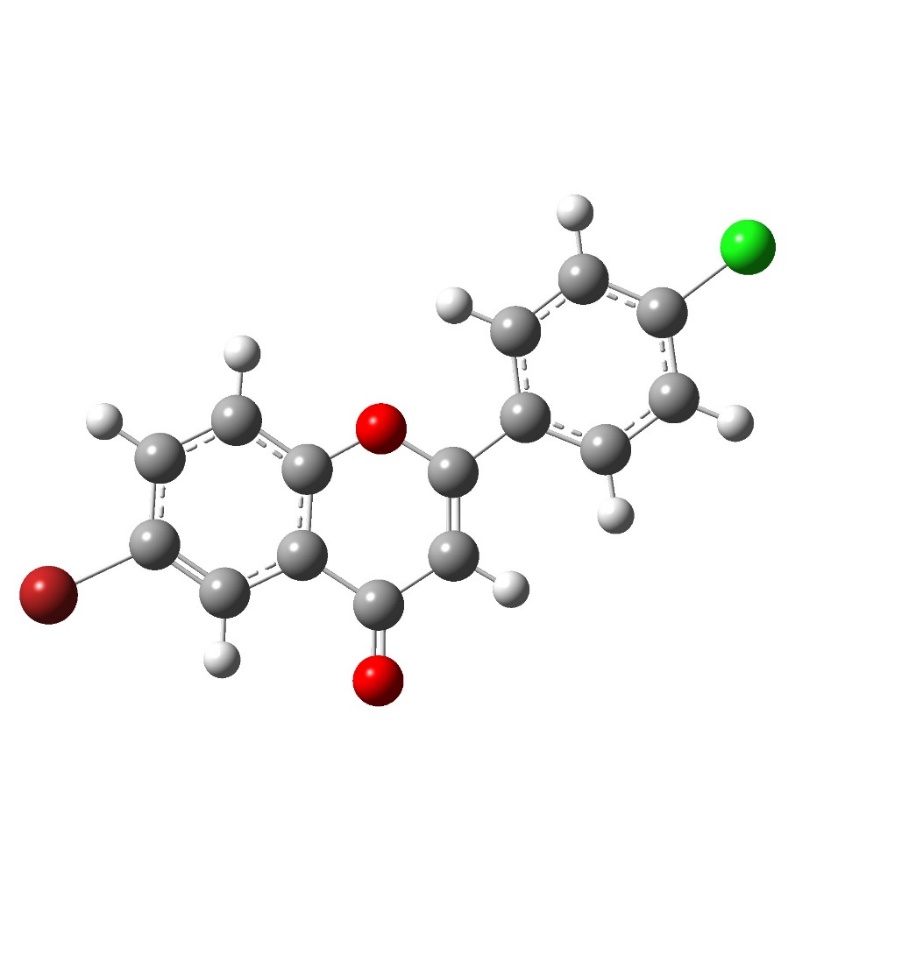** | | |
| --- | --- | --- | --- | --- | --- |
| Center  Number | Atom | Standard orientation: Coordinates (Angstroms) | | | |
|  |  | X | | Y | Z |
| 1 | Br | 4.56675 | | -3.213993 | 0 |
| 2 | Cl | -5.045752 | | 5.083736 | 0 |
| 3 | O | 0 | | 0.745442 | 0 |
| 4 | O | -0.973067 | | -3.228345 | 0 |
| 5 | C | 0.730821 | | -1.562657 | 0 |
| 6 | C | 1.0015 | | -0.19002 | 0 |
| 7 | C | -1.308346 | | 0.353493 | 0 |
| 8 | C | -2.227885 | | 1.507025 | 0 |
| 9 | C | -0.674413 | | -2.035595 | 0 |
| 10 | C | -1.657144 | | -0.959595 | 0 |
| 11 | C | 1.801036 | | -2.46926 | 0 |
| 12 | C | 2.313741 | | 0.292462 | 0 |
| 13 | C | -3.622343 | | 1.327047 | 0 |
| 14 | C | -1.725203 | | 2.819296 | 0 |
| 15 | C | 3.09961 | | -1.990796 | 0 |
| 16 | C | 3.365231 | | -0.613025 | 0 |
| 17 | C | -4.486444 | | 2.415203 | 0 |
| 18 | C | -2.581456 | | 3.916596 | 0 |
| 19 | C | -3.959292 | | 3.70761 | 0 |
| 20 | H | -2.69803 | | -1.253945 | 0 |
| 21 | H | 1.573554 | | -3.528848 | 0 |
| 22 | H | 2.491563 | | 1.362214 | 0 |
| 23 | H | -4.04849 | | 0.330501 | 0 |
| 24 | H | -0.654896 | | 2.982178 | 0 |
| 25 | H | 4.389888 | | -0.260005 | 0 |
| 26 | H | -5.55993 | | 2.265547 | 0 |
| 27 | H | -2.184074 | | 4.925112 | 0 |

**Table S10**

Optimized structure for Quercetin (**10**) and cartesian Z-matrix.

| **Quercetin (10)** | | | **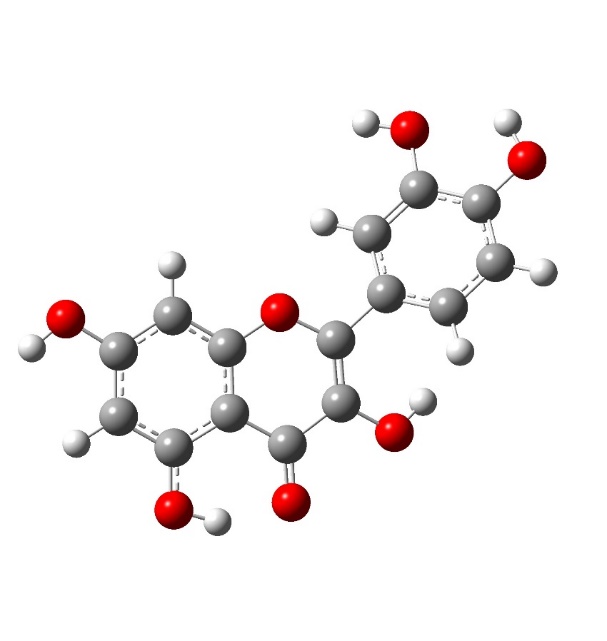** | | |
| --- | --- | --- | --- | --- | --- |
| Center  Number | Atom | Standard orientation: Coordinates (Angstroms) | | | |
|  |  | X | | Y | Z |
| 1 | O | -0.276398 | | -0.695516 | -0.148345 |
| 2 | O | 0.411989 | | 2.869263 | 0.323475 |
| 3 | O | -4.517327 | | 1.517558 | 0.194147 |
| 4 | O | -2.294118 | | 2.830896 | 0.307832 |
| 5 | O | -4.232884 | | -3.220244 | -0.307702 |
| 6 | O | 4.439196 | | -2.221369 | 0.849189 |
| 7 | O | 5.994598 | | -0.357393 | -0.262256 |
| 8 | C | -2.364719 | | 0.478525 | 0.02713 |
| 9 | C | -1.640346 | | -0.718104 | -0.110773 |
| 10 | C | 0.417005 | | 0.486192 | -0.051388 |
| 11 | C | 1.872001 | | 0.281732 | -0.115227 |
| 12 | C | -0.223406 | | 1.679076 | 0.139422 |
| 13 | C | -1.684929 | | 1.749639 | 0.164823 |
| 14 | C | -3.788656 | | 0.405749 | 0.056656 |
| 15 | C | -2.256024 | | -1.958787 | -0.223629 |
| 16 | C | -3.653409 | | -1.995039 | -0.194483 |
| 17 | C | 2.432082 | | -0.899873 | 0.41145 |
| 18 | C | -4.421627 | | -0.830323 | -0.055919 |
| 19 | C | 2.728226 | | 1.227012 | -0.706916 |
| 20 | C | 3.799965 | | -1.109131 | 0.359285 |
| 21 | C | 4.106139 | | 1.012707 | -0.753338 |
| 22 | C | 4.654934 | | -0.148564 | -0.218329 |
| 23 | H | -1.678092 | | -2.866553 | -0.337371 |
| 24 | H | 1.783934 | | -1.647968 | 0.85778 |
| 25 | H | -5.506431 | | -0.86856 | -0.032628 |
| 26 | H | 2.316985 | | 2.110534 | -1.1834 |
| 27 | H | 4.769167 | | 1.733776 | -1.218791 |
| 28 | H | 1.33863 | | 2.7 | 0.54962 |
| 29 | H | -3.863972 | | 2.271156 | 0.2659 |
| 30 | H | -5.193944 | | -3.120872 | -0.281286 |
| 31 | H | 3.795472 | | -2.843871 | 1.210175 |
| 32 | H | 6.17438 | | -1.218075 | 0.1467 |

**Table S11**

Optimized structure for Pedalitin (**11**) and cartesian Z-matrix.

| **Pedalitin (11)** | | | 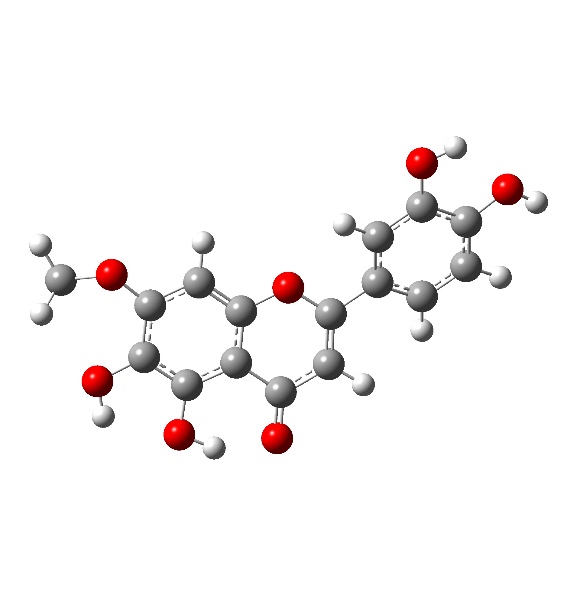 | | |
| --- | --- | --- | --- | --- | --- |
| Center  Number | Atom | Standard orientation: Coordinates (Angstroms) | | | |
|  |  | X | | Y | Z |
| 1 | F | -6.80599 | | -0.97955 | -0.80090 |
| 2 | O | 2.94851 | | 0.57280 | -0.05546 |
| 3 | O | -0.61783 | | -0.07157 | -0.30455 |
| 4 | O | 0.39656 | | -2.61984 | -0.17226 |
| 5 | O | -1.06658 | | -0.56265 | 1.86704 |
| 6 | C | 1.59723 | | 0.77245 | -0.05537 |
| 7 | C | 2.64889 | | -1.82948 | -0.08536 |
| 8 | C | 3.46877 | | -0.69461 | -0.06863 |
| 9 | C | 0.73718 | | -0.28029 | -0.11517 |
| 10 | C | 1.18280 | | -1.67677 | -0.12184 |
| 11 | C | 1.24846 | | 2.20480 | -0.02568 |
| 12 | C | 3.25248 | | -3.09842 | -0.09155 |
| 13 | C | 4.86346 | | -0.80028 | -0.05473 |
| 14 | C | 0.17514 | | 2.67111 | 0.74959 |
| 15 | C | 2.01920 | | 3.12316 | -0.75720 |
| 16 | C | 4.63288 | | -3.21795 | -0.07717 |
| 17 | C | 5.43625 | | -2.06457 | -0.05895 |
| 18 | C | -0.13099 | | 4.02994 | 0.77230 |
| 19 | C | 1.70413 | | 4.47933 | -0.73409 |
| 20 | C | -1.45531 | | -0.43720 | 0.72810 |
| 21 | C | -2.86040 | | -0.57207 | 0.27821 |
| 22 | C | 0.62621 | | 4.93575 | 0.02720 |
| 23 | C | -3.83759 | | -0.80960 | 1.25639 |
| 24 | C | -3.23252 | | -0.47973 | -1.07100 |
| 25 | C | -5.17368 | | -0.94809 | 0.89924 |
| 26 | C | -4.56717 | | -0.61627 | -1.44039 |
| 27 | C | -5.51398 | | -0.84726 | -0.44704 |
| 28 | H | 2.59899 | | -3.96418 | -0.10803 |
| 29 | H | 5.46374 | | 0.10274 | -0.03769 |
| 30 | H | -0.38890 | | 1.97518 | 1.35955 |
| 31 | H | -0.38889800 | | 1.97518100 | 1.35954600 |
| 32 | H | 2.85823200 | | 2.76693800 | -1.34511800 |
| 33 | H | 5.09626600 | | -4.19930000 | -0.07931800 |
| 34 | H | 6.51782200 | | -2.15889300 | -0.04662300 |
| 35 | H | -0.95753600 | | 4.38247100 | 1.38169600 |
| 36 | H | 2.30038100 | | 5.18026900 | -1.31044900 |
| 37 | H | 0.38286600 | | 5.99392100 | 0.04621900 |
| 38 | H | -3.52795000 | | -0.88535900 | 2.29280800 |
| 39 | H | -2.47366000 | | -0.30705900 | -1.82428900 |
| 40 | H | -5.94773700 | | -1.13253700 | 1.63564300 |
| 41 | H | -4.88256200 | | -0.55176000 | -2.47567300 |
| 42 | H | -1.72569800 | | -3.52967200 | 0.10591400 |

**Table S12**

Optimized structure Dihydrorobinetin (**12**) and cartesian Z-matrix.

| **Dihydrorobinetin (12)** | | | 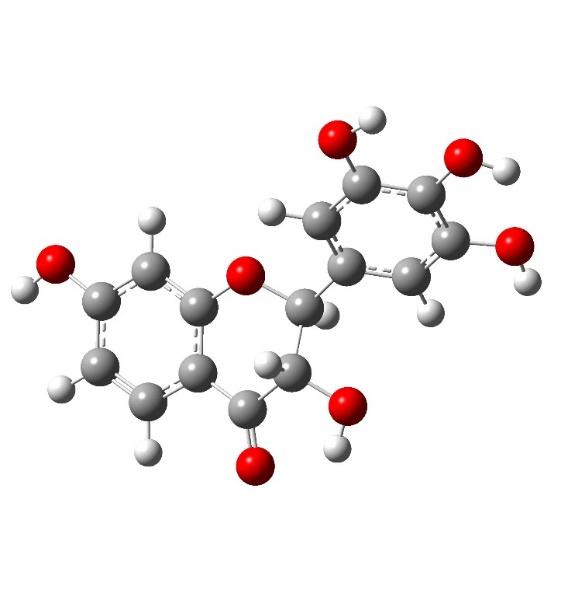 | | |
| --- | --- | --- | --- | --- | --- |
| Center  Number | Atom | Standard orientation: Coordinates (Angstroms) | | | |
|  |  | X | | Y | Z |
| 1 | O | 0.852398 | | -0.699804 | -0.189837 |
| 2 | O | -0.315459 | | 2.718168 | 0.48469 |
| 3 | O | 2.309848 | | 3.026522 | 0.601821 |
| 4 | O | -3.480316 | | -2.585015 | 1.241333 |
| 5 | O | -4.802011 | | 1.246887 | -1.189139 |
| 6 | O | 5.109718 | | -2.632918 | -0.575343 |
| 7 | O | -5.425637 | | -1.019077 | 0.162853 |
| 8 | C | 0.024887 | | 0.471048 | -0.370727 |
| 9 | C | 0.441811 | | 1.548466 | 0.64922 |
| 10 | C | -1.422171 | | 0.04973 | -0.251941 |
| 11 | C | 1.92918 | | 1.865615 | 0.470535 |
| 12 | C | 2.786589 | | 0.728367 | 0.160845 |
| 13 | C | 2.197206 | | -0.522545 | -0.132864 |
| 14 | C | -2.408428 | | 0.866276 | -0.821013 |
| 15 | C | -1.781704 | | -1.106916 | 0.443976 |
| 16 | C | 4.188496 | | 0.831863 | 0.175235 |
| 17 | C | 2.991619 | | -1.64215 | -0.375918 |
| 18 | C | -3.128619 | | -1.452168 | 0.569784 |
| 19 | C | -3.749069 | | 0.520752 | -0.678917 |
| 20 | C | 4.380132 | | -1.513864 | -0.333513 |
| 21 | C | 4.98754 | | -0.269934 | -0.064437 |
| 22 | C | -4.115489 | | -0.636919 | 0.009497 |
| 23 | H | 0.214423 | | 0.872419 | -1.377331 |
| 24 | H | 0.311045 | | 1.119191 | 1.659396 |
| 25 | H | -2.131666 | | 1.778863 | -1.34007 |
| 26 | H | -1.03156 | | -1.755769 | 0.87721 |
| 27 | H | 4.622446 | | 1.801683 | 0.395876 |
| 28 | H | 2.53535 | | -2.596872 | -0.607413 |
| 29 | H | 0.336732 | | 3.439042 | 0.573538 |
| 30 | H | 6.071101 | | -0.186983 | -0.0418 |
| 31 | H | -4.447452 | | -2.646139 | 1.222892 |
| 32 | H | -4.471508 | | 2.071472 | -1.566626 |
| 33 | H | 6.051399 | | -2.418535 | -0.533787 |
| 34 | H | -5.980527 | | -0.342213 | -0.251479 |

**Table S13**

Optimized structure for Phlorizine (**13**) and cartesian Z-matrix.

| **Phlorizine (13)** | | | **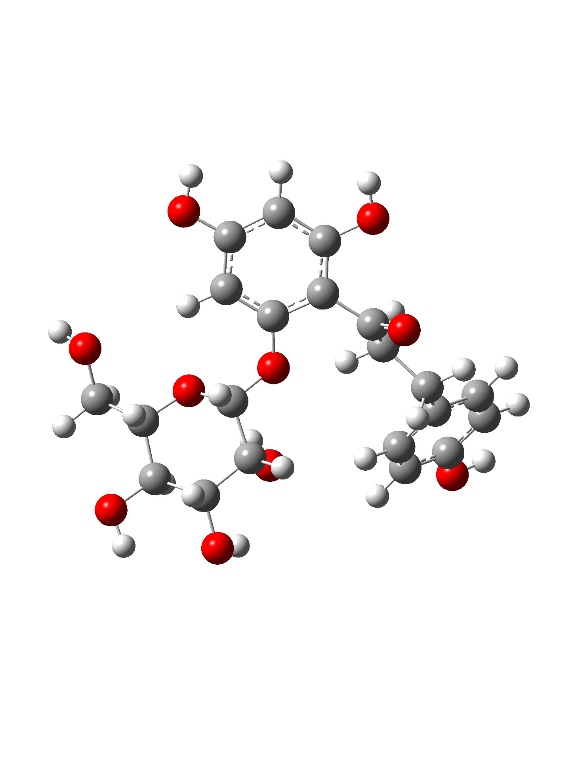** | | |
| --- | --- | --- | --- | --- | --- |
| Center  Number | Atom | Standard orientation: Coordinates (Angstroms) | | | |
|  |  | X | | Y | Z |
| 1 | O | -2.279537 | | 1.067336 | 0.590067 |
| 2 | O | -0.790726 | | -0.410412 | -0.327831 |
| 3 | O | -1.204779 | | 4.077332 | -1.987244 |
| 4 | O | -3.650995 | | 4.271564 | -0.481637 |
| 5 | O | 0.154531 | | 2.184321 | -0.471695 |
| 6 | O | -4.785584 | | 1.082958 | 1.953603 |
| 7 | O | 0.147521 | | -5.028242 | -0.401534 |
| 8 | O | 1.280076 | | -2.90757 | -2.100634 |
| 9 | O | -4.111877 | | -3.477429 | 1.237684 |
| 10 | O | 6.600401 | | 2.39311 | 1.869486 |
| 11 | C | -1.89861 | | 2.937881 | -1.502716 |
| 12 | C | -2.661076 | | 3.295739 | -0.216032 |
| 13 | C | -0.940283 | | 1.75544 | -1.282793 |
| 14 | C | -3.316245 | | 2.038295 | 0.361991 |
| 15 | C | -1.705597 | | 0.601273 | -0.624102 |
| 16 | C | -4.040858 | | 2.260297 | 1.674461 |
| 17 | C | -1.241682 | | -1.688609 | -0.052006 |
| 18 | C | -0.331798 | | -2.714359 | -0.365948 |
| 19 | C | -2.503058 | | -1.935693 | 0.485214 |
| 20 | C | 1.004585 | | -2.429264 | -1.015474 |
| 21 | C | -0.741699 | | -4.035282 | -0.123151 |
| 22 | C | 1.992862 | | -1.574737 | -0.237688 |
| 23 | C | -2.874113 | | -3.265108 | 0.707568 |
| 24 | C | 3.165514 | | -1.065979 | -1.090784 |
| 25 | C | -2.002977 | | -4.315688 | 0.412249 |
| 26 | C | 4.09936 | | -0.161303 | -0.316204 |
| 27 | C | 3.726047 | | 1.155677 | -0.000327 |
| 28 | C | 5.348854 | | -0.607364 | 0.124956 |
| 29 | C | 4.565056 | | 1.993979 | 0.726973 |
| 30 | C | 6.202638 | | 0.221265 | 0.855789 |
| 31 | C | 5.811266 | | 1.527065 | 1.159071 |
| 32 | H | -2.640632 | | 2.675604 | -2.267942 |
| 33 | H | -1.931982 | | 3.670866 | 0.523251 |
| 34 | H | -0.516804 | | 1.43297 | -2.238949 |
| 35 | H | -4.043891 | | 1.641166 | -0.363076 |
| 36 | H | -2.49953 | | 0.22366 | -1.289229 |
| 37 | H | -3.295961 | | 2.4713 | 2.456785 |
| 38 | H | -4.687442 | | 3.14149 | 1.563972 |
| 39 | H | -0.377821 | | 4.10868 | -1.477935 |
| 40 | H | -3.215859 | | 4.945047 | -1.026678 |
| 41 | H | -0.089963 | | 2.014302 | 0.451065 |
| 42 | H | -5.132754 | | 1.147542 | 2.851451 |
| 43 | H | -3.171119 | | -1.13483 | 0.774156 |
| 44 | H | 1.460245 | | -0.742185 | 0.229127 |
| 45 | H | 2.361159 | | -2.196245 | 0.592657 |
| 46 | H | 2.755348 | | -0.529728 | -1.95451 |
| 47 | H | 3.711622 | | -1.921074 | -1.499352 |
| 48 | H | -2.296161 | | -5.346072 | 0.605512 |
| 49 | H | 2.759154 | | 1.530036 | -0.330005 |
| 50 | H | 5.668091 | | -1.620237 | -0.108213 |
| 51 | H | -0.260463 | | -5.883373 | -0.214803 |
| 52 | H | -4.252992 | | -4.426553 | 1.349859 |
| 53 | H | 4.276715 | | 3.0129 | 0.964607 |
| 54 | H | 7.17243 | | -0.147551 | 1.184734 |
| 55 | H | 7.427239 | | 1.94739 | 2.095112 |

**Table S14**

Optimized structure for Hyperin (**14**) and cartesian Z-matrix.

| **Hyperin (14)** | | | **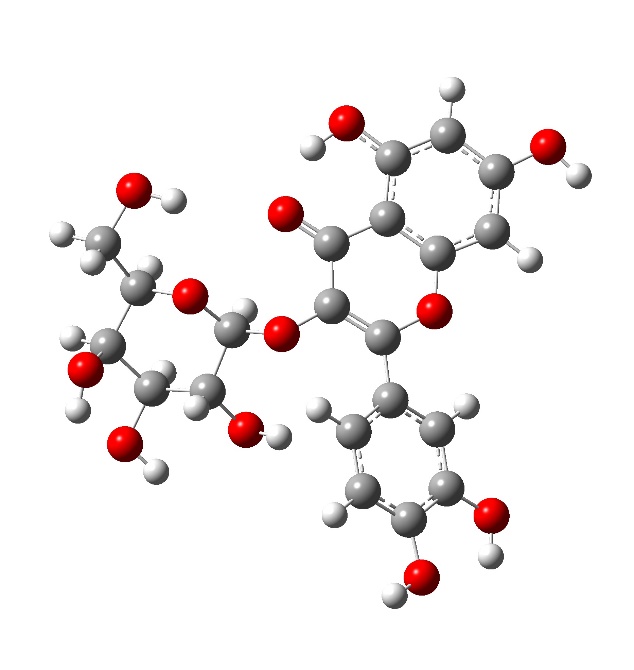** | | |
| --- | --- | --- | --- | --- | --- |
| Center  Number | Atom | Standard orientation: Coordinates (Angstroms) | | | |
|  |  | X | | Y | Z |
| 1 | O | -2.692129 | | -1.267268 | -0.585667 |
| 2 | O | -0.836066 | | 0.045304 | -0.714834 |
| 3 | O | -4.807016 | | 1.513995 | 1.572432 |
| 4 | O | -5.407088 | | -0.136205 | -0.50435 |
| 5 | O | -2.03165 | | 1.820346 | 1.257211 |
| 6 | O | -3.438408 | | -3.889505 | -0.961332 |
| 7 | O | 2.730612 | | 0.223388 | -0.127361 |
| 8 | O | -0.131813 | | -2.67485 | -0.393246 |
| 9 | O | 1.707718 | | -4.463549 | 0.013122 |
| 10 | O | 6.138406 | | -3.005968 | 0.633112 |
| 11 | O | 3.027794 | | 5.01468 | 0.838119 |
| 12 | O | 1.092406 | | 6.21315 | -0.570049 |
| 13 | C | -3.934099 | | 0.415355 | 1.339749 |
| 14 | C | -4.740453 | | -0.677724 | 0.622166 |
| 15 | C | -2.735587 | | 0.849621 | 0.495353 |
| 16 | C | -3.787015 | | -1.792535 | 0.180707 |
| 17 | C | -1.89183 | | -0.377737 | 0.140258 |
| 18 | C | -4.390681 | | -2.87382 | -0.707763 |
| 19 | C | 0.443718 | | -0.363189 | -0.436099 |
| 20 | C | 1.430564 | | 0.583423 | -0.34567 |
| 21 | C | 0.750041 | | -1.789375 | -0.31205 |
| 22 | C | 2.141241 | | -2.104467 | -0.064477 |
| 23 | C | 3.098502 | | -1.082982 | 0.023544 |
| 24 | C | 1.284916 | | 2.046755 | -0.436491 |
| 25 | C | 2.581995 | | -3.45558 | 0.089116 |
| 26 | C | 4.445745 | | -1.33398 | 0.256113 |
| 27 | C | 2.216365 | | 2.862661 | 0.235876 |
| 28 | C | 0.266689 | | 2.655554 | -1.192698 |
| 29 | C | 3.926746 | | -3.725291 | 0.32297 |
| 30 | C | 4.842746 | | -2.668847 | 0.403683 |
| 31 | C | 2.125259 | | 4.24581 | 0.173304 |
| 32 | C | 0.176682 | | 4.048132 | -1.255336 |
| 33 | C | 1.093827 | | 4.8429 | -0.576762 |
| 34 | H | -3.555525 | | 0.017412 | 2.295302 |
| 35 | H | -5.464589 | | -1.10393 | 1.336741 |
| 36 | H | -3.107849 | | 1.265465 | -0.450775 |
| 37 | H | -3.38736 | | -2.277364 | 1.087238 |
| 38 | H | -1.475407 | | -0.848054 | 1.043777 |
| 39 | H | -4.757881 | | -2.404307 | -1.630624 |
| 40 | H | -5.241949 | | -3.339396 | -0.19991 |
| 41 | H | -4.236026 | | 2.264763 | 1.794139 |
| 42 | H | -5.769174 | | 0.711786 | -0.203361 |
| 43 | H | -1.350847 | | 2.208915 | 0.687458 |
| 44 | H | -2.613281 | | -3.434792 | -1.193899 |
| 45 | H | 5.150153 | | -0.511159 | 0.313404 |
| 46 | H | 3.017919 | | 2.426072 | 0.818109 |
| 47 | H | -0.430587 | | 2.048225 | -1.752884 |
| 48 | H | 4.263677 | | -4.747405 | 0.442105 |
| 49 | H | -0.604496 | | 4.513823 | -1.851487 |
| 50 | H | 0.816445 | | -4.054502 | -0.156134 |
| 51 | H | 6.679434 | | -2.205865 | 0.66768 |
| 52 | H | 2.802783 | | 5.943784 | 0.678201 |
| 53 | H | 0.3702 | | 6.550283 | -1.115277 |

**Table S15**

Predicted biological activity of the flavones (**1**−**14**) using PASS.

| **Flavonoids** | **Biological Activity** | | | | | | | | | | |
| --- | --- | --- | --- | --- | --- | --- | --- | --- | --- | --- | --- |
|  | **Membrane Integrity Agonist** | |  | **Membrane Permeability Inhibitor** | |  | **Anticarcinogenic** | |  | **Antioxidant** | |
|  | **Pa** | **Pi** |  | **Pa** | **Pi** |  | **Pa** | **Pi** |  | **Pa** | **Pi** |
| 1 | 0.947 | 0.004 |  | 0.914 | 0.003 |  | 0.408 | 0.029 |  | 0.469 | 0.008 |
| 2 | 0.963 | 0.003 |  | 0.937 | 0.003 |  | 0.539 | 0.016 |  | 0.631 | 0.004 |
| 3 | 0.957 | 0.003 |  | 0.935 | 0.003 |  | 0.532 | 0.017 |  | 0.521 | 0.006 |
| 4 | 0.958 | 0.003 |  | 0.931 | 0.003 |  | 0.542 | 0.016 |  | 0.546 | 0.005 |
| 5 | 0.968 | 0.002 |  | 0.945 | 0.002 |  | 0.578 | 0.014 |  | 0.752 | 0.004 |
| 6 | 0.962 | 0.003 |  | 0.943 | 0.002 |  | 0.572 | 0.012 |  | 0.631 | 0.004 |
| 7 | 0.965 | 0.003 |  | 0.946 | 0.002 |  | 0.618 | 0.012 |  | 0.708 | 0.004 |
| 8 | 0.890 | 0.013 |  | 0.792 | 0.011 |  | 0.180 | 0.140 |  | 0.752 | 0.004 |
| 9 | 0.890 | 0.014 |  | 0.821 | 0.007 |  | 0.219 | 0.102 |  | 0.231 | 0.042 |
| 10 | 0.965 | 0.003 |  | 0.946 | 0.002 |  | 0.618 | 0.012 |  | 0.708 | 0.004 |
| 11. | 0.936 | 0.004 |  | 0.922 | 0.003 |  | 0.192 | 0.129 |  | 0.191 | 0.061 |
| 12 | - | - |  | - | - |  | 0.686 | 0.009 |  | 0.719 | 0.004 |
| 13 | 0.962 | 0.003 |  | 0.785 | 0.012 |  | 0.791 | 0.005 |  | 0.936 | 0.002 |
| 14 | 0.934 | 0.005 |  | 0.813 | 0.008 |  | 0.870 | 0.003 |  | 0.655 | 0.004 |
| 15 | 0.989 | 0.001 |  | 0.981 | 0.001 |  | 0.965 | 0.001 |  | 0.913 | 0.003 |
| Tropoflavin | 0.958 | 0.003 |  | 0.948 | 0.002 |  | 0.625 | 0.012 |  | 0.741 | 0.004 |
| Taxifolin | 0.973 | 0.002 |  | 0.850 | 0.005 |  | 0.821 | 0.005 |  | 0.938 | 0.002 |

**Table S16**

Shows the active site residues of proteins (PDB: 1P60) and (PDB: 1X2J).

| **Active Site of PDB: 1X2J** | |
| --- | --- |
| **Pocket**  **Surface area Å^2^** | 402.149 |
| **Pocket**  **Volume (SA) Å^3^** | 419.304 |
| **Active Site residues** | 324 VAL, 364 GLY, 365 LEU, 366 ALA, 367 GLY, 368 CYS, 369 VAL, 415 ARG,  416 ILE, 17 GLY, 418 VAL, 419 GLY, 420 VAL, 462 GLY, 463 VAL, 464 GLY, 465 VAL, 466 ALA, 467 VAL, 509 GLY, 510 ALA, 511 GLY, 512 VAL, 513 CYS, 514 VAL, 556 ALA, 557 LEU, 558 GLY, 559 ILE, 560 THR, 561 VAL, 603 GLY, 604 VAL, 606 GLY, 607 ALA, 608 VAL |
| **Active Site of PDB: 1p60** | |
| **Pocket**  **Surface area Å^2^** | 320.855 |
| **Pocket**  **Volume (SA) Å^3^** | 356.089 |
| **Active Site residues** | 61 VAL, 62 GLY, 63 SER, 64 THR, 65 GLY, 66 ASP, 69 GLU, 73 MET, 7 SER, 75 GLN, 76 LYS, 78 GLY, 79 GLY, 134 ARG, 135 TYR, 149 GLU, 150 THR, 152 TRP, 153 THR, 156 GLN, 160 THR, 222 ASP. 223 LYS, 223 THR, 224 ASP. |
| **Active Site of PDB: 4UV7** | |
| **Pocket**  **Surface area Å^2^** | 3220.564 |
| **Pocket**  **Volume (SA) Å^3^** | 6125.590 |
| **Active Site residues** | 1 LEU, 2 GLU, 3 GLU, 4 LYS, 6 VAL, 6 GLN, 9 GLY, 10 THR, 11 SER, 13 LYS, 36 VAL, 38 LEU, 39 GLY, 40 ASN, 60 GLU, 62 ALA, 63 GLY, 64 TYR, 8 ARG, 86 ASN, 87 MET, 88 TYR, 89 TYR, 90 GLU, 91 ASN, 118 GLU, 120 LEU, 121 HIS. 134 ILE, 144 VAl, 145 SER, 188 LYS, 197 GLY, 198 ARG, 209 HIS, 210 ASN, 211 GLY, 212 CYS, 221 GLU, 225 LEU, 226 VAL, 227 CYS. 228 ARG, 229 LYS, 230 PHE, 231 ARG, 237 LYS, 241 PRO 242 PRO 243 LEU 244 MET 245 LYS, 509 ARG, 511 CYS, 524 GLU, 532 ILE, 533 GLU, 580 ASN, 582 LEU, 584 TRP, 601 TRP, 602 TYR |
| **Active Site of PDB: 1V40** | |
| **Pocket**  **Surface area Å^2^** | 4123.958 |
| **Pocket**  **Volume (SA) Å^3^** | 5317.225 |
| **Active Site residues** | 208 TYR, 209 PHE, 211 MET, 212 RG, 213 GLY, 214 ARG, 217 ILE, 236 GLN, 239 TRP, 243 LYS, 244 SER, 246 LEU, 247 PRO, 248 PHE, 249 GLY, 250 LYS, 251 ILE, 252 HIS, 263 GLU, 264 SER, 265 LEU, 293 ASP, 294 THR, 296 ASP, 297 ASP, 230 PHE, 299 MET, 300 SER, 301 CYS, 302 PHE, 303 PRO, 304 TRP, 305 ALA, 306 GLU, 318 GLU 319 LEU, 322 TYR, 323 ASN, 326 HIS, 327 LEU, 330 ASP, 352 TYR, 355 ILE, 356 CYS, 399 LEU, 408 TYR, 630 GLU, 631 ASP, 632 HIS, 632 ARG, 635 ILE, 636 GLU, 790 TRP, 793 ARG, 794 ARG, 795 PRO, 796 GLN, 797 THR |
| **Active Site of PDB: 1YQ7** | |
| **Pocket**  **Surface area Å^2^** | 851.723 |
| **Pocket**  **Volume (SA) Å^3^** | 437.561 |
| **Active Site residues** | 27 GLU, 67 ALA, 69 GLY, 70 GLY, 71 LYS, 72 TYR, 73 ASN, 74 ARG, 76 LEU, 77 THR, 79 VAL 80 ALA 54 GLU, 107 GLU, 110 GLN, 113 PHE, 114 LEU, 117 ASP, 118 ASP, 120 MET, 121 ASP, 125 THR, 129 ARG, 257 ASP, 258 ASP, 260 LEU, 261 ASP, 264 GLY, 266 PRO, 268 VAL, 270 GLY, 271 LYS. 280 LYS, 281 CYS, 282 SER, 361 LYS, 362 ILE |

**
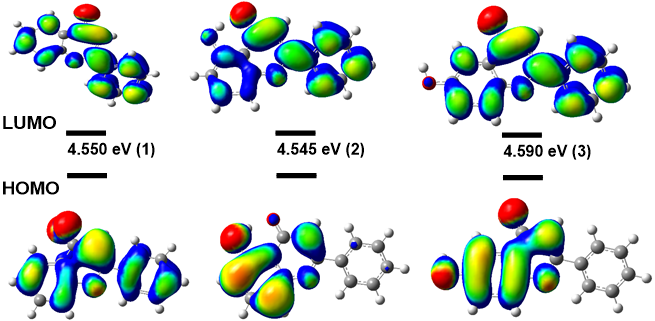
**

**Fig. S1.** Molecular orbitals (MO) of HOMO and LUMO gap for compounds (**1−3**).

**Fig. S2.** Molecular orbitals (MO) of HOMO and LUMO gap for compounds (**4−6**).

**Fig. S3.** Molecular orbitals (MO) of HOMO and LUMO gap for compounds (**7−9**).

**Fig. S4.** Molecular orbitals (MO) of HOMO and LUMO gap for compounds (**10−12**).

**Fig. S5.** Molecular orbitals (MO) of HOMO and LUMO gap for compounds (**13−14**).


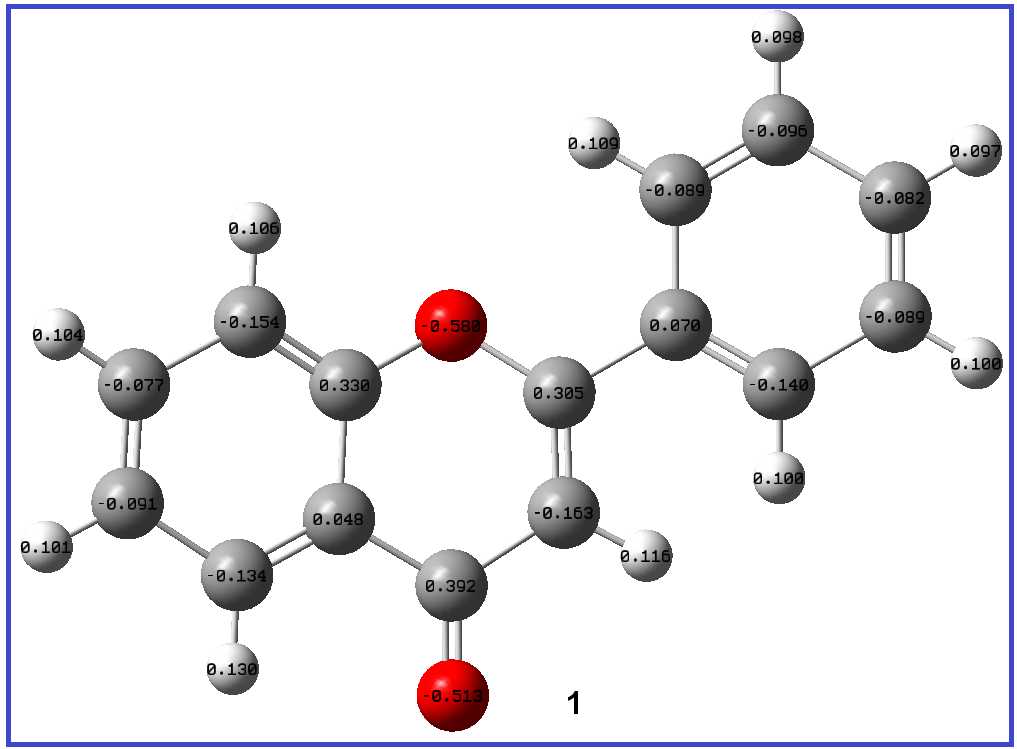


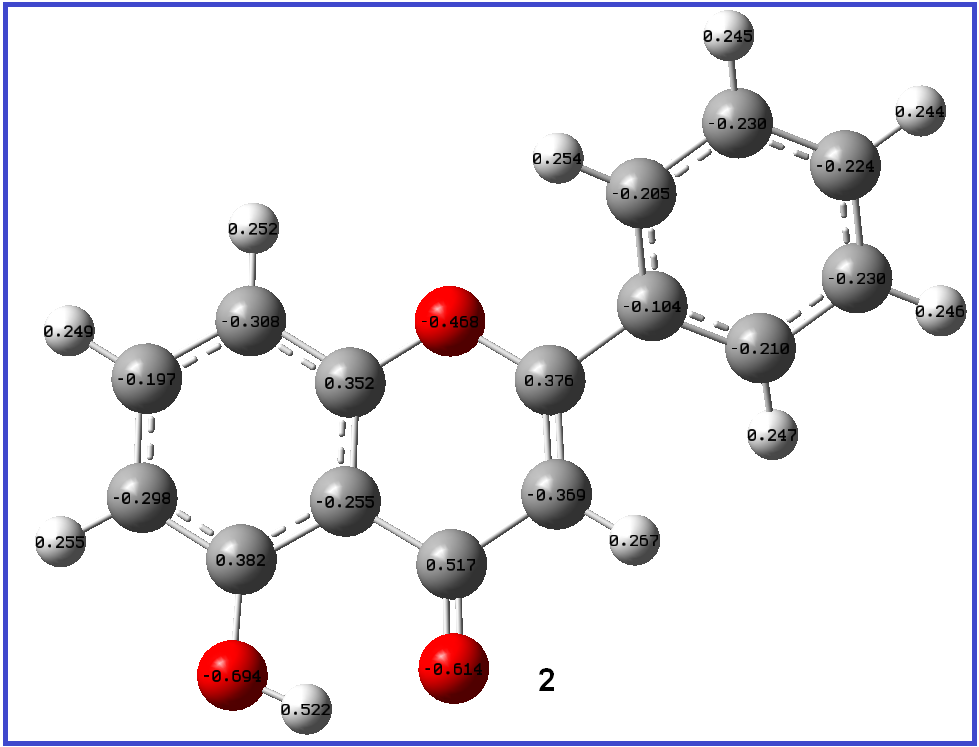


**Fig. S6.** NBO charges structures for compounds **1** and **2**.

**Fig. S7.** NBO charges structures for compounds **3** and **5**.

**Fig. S8.** NBO charges structures for compounds **6** and **7.**


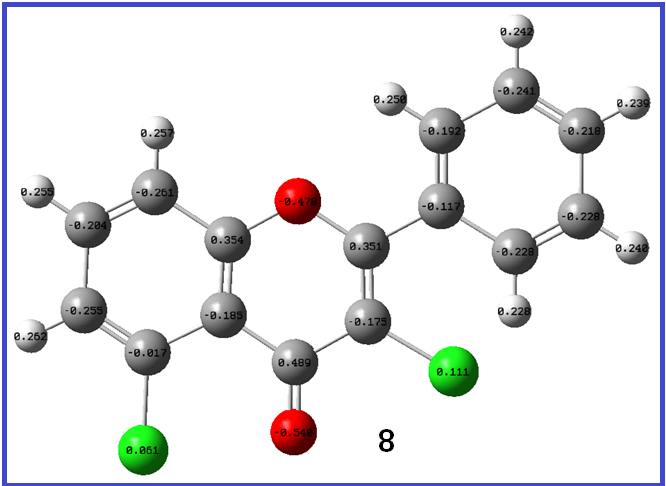

**Fig. S9.** NBO charges structures for compounds **8** and **9.**

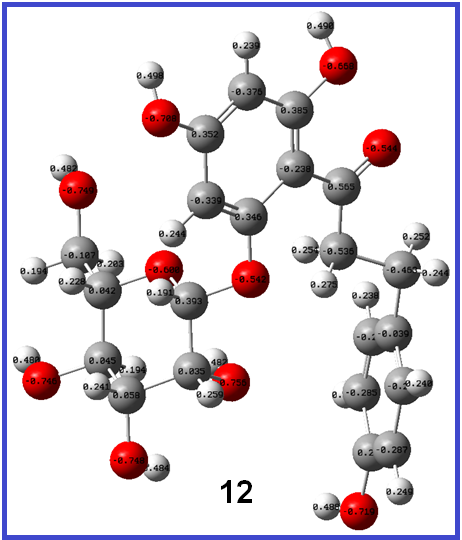


**Fig. S10.** NBO charges structures for compounds **11** and **12.**


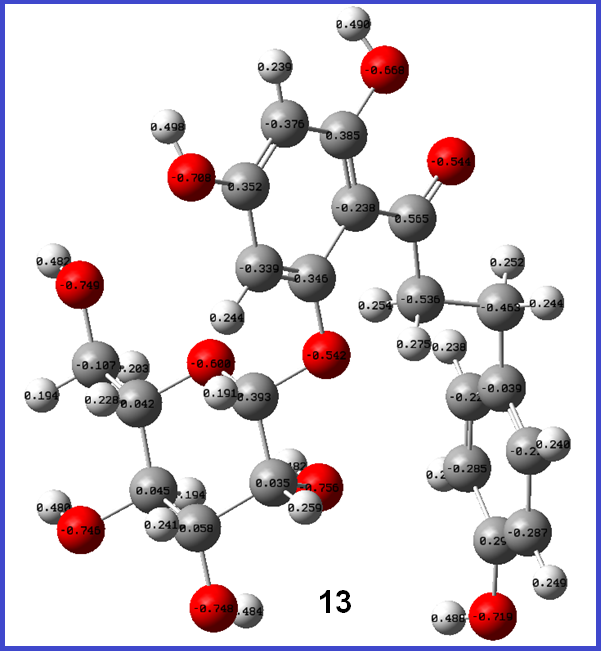

**Fig. S11.** NBO charges structures for compounds **13** and **14.**

**
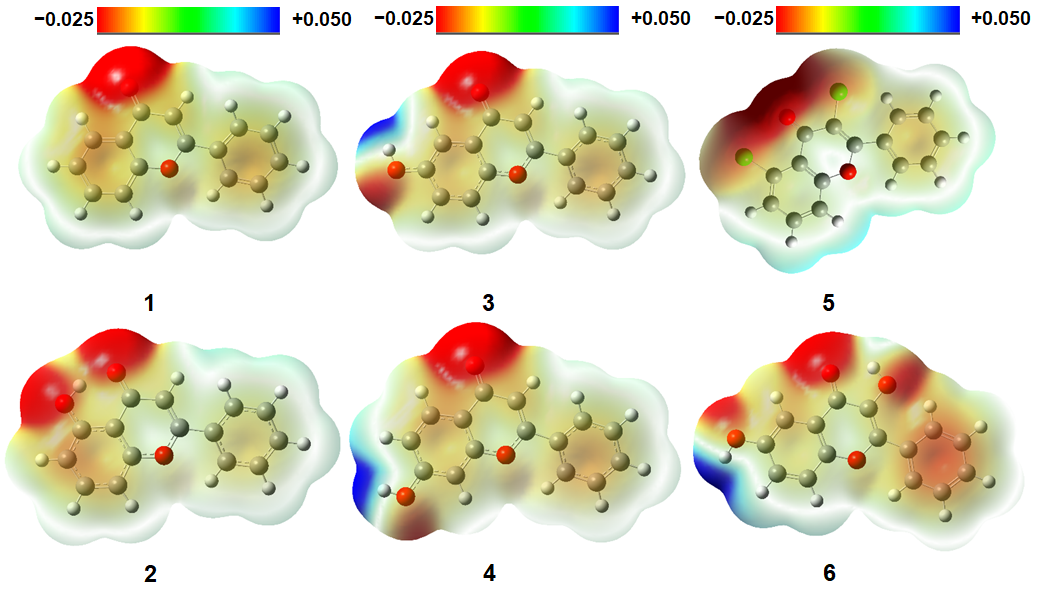
**

**Fig. S12.** Maps of electrostatic potential (0.02 electrons Bohr^−3^ surface) (red = electron-rich, blue = electron-deficient) for compounds **1** to **6**.

**
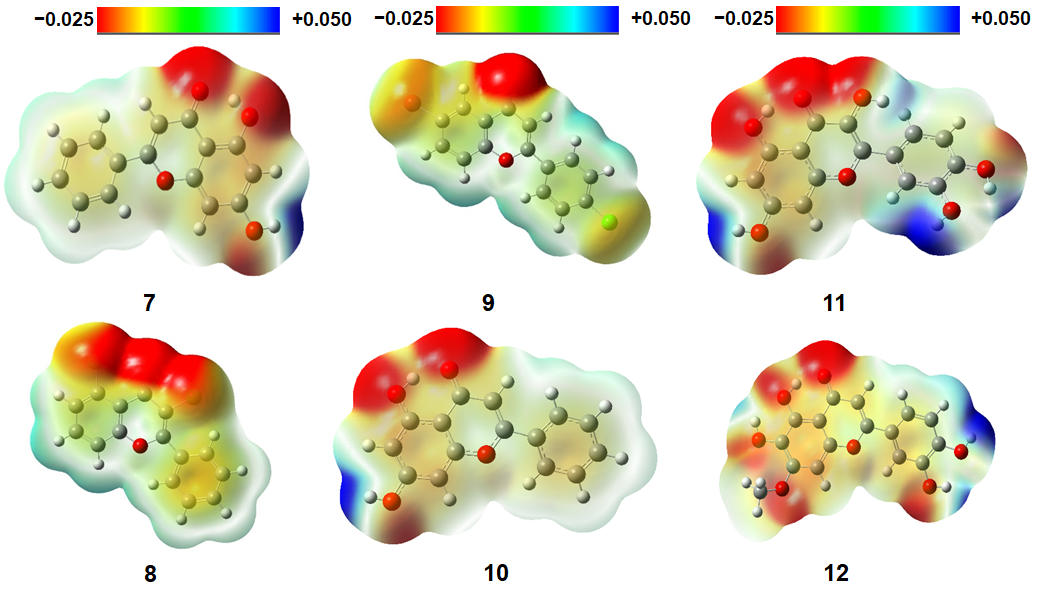
**

**Fig. S13.** Maps of electrostatic potential (0.02 electrons Bohr^−3^ surface) (red = electron-rich, blue = electron-deficient) for compounds **7** to **12**.

**
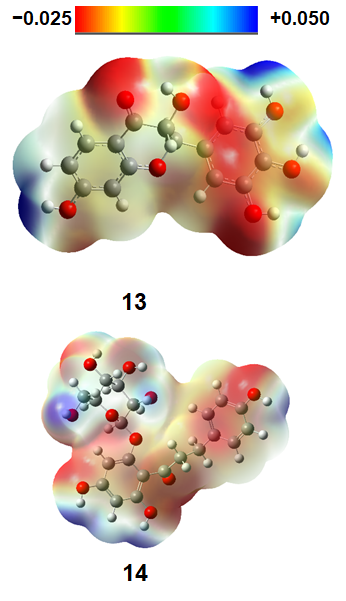
**

**Fig. S14.** Maps of electrostatic potential (0.02 electrons Bohr^−3^ surface) (red = electron-rich, blue = electron-deficient) for compounds **13** to **14**.

**
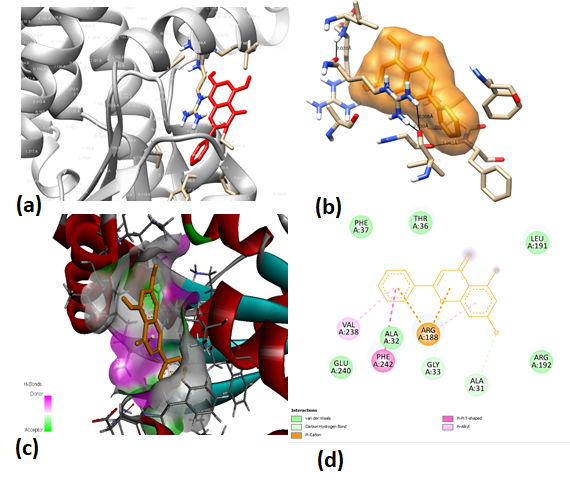
**

**Fig. S15.** Molecular docking poses: (a) Ligand in protein pocket; (b) Active site; (c) Hydrogen bonding in solid; (d) Ligand-protein interaction for 2D diagram of compound **7** with PDB (1P60).


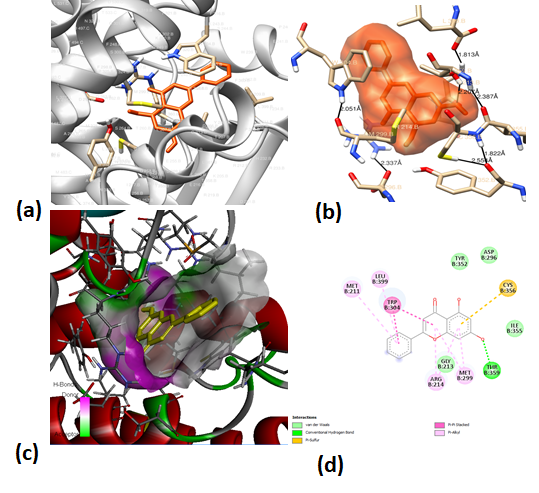


**Fig. S16.** Molecular docking poses: (a) Ligand in protein pocket; (b) Active site; (c) Hydrogen bonding in solid; (d) Ligand-protein interaction for 2D diagram of compound **7** with PDB (1V40).


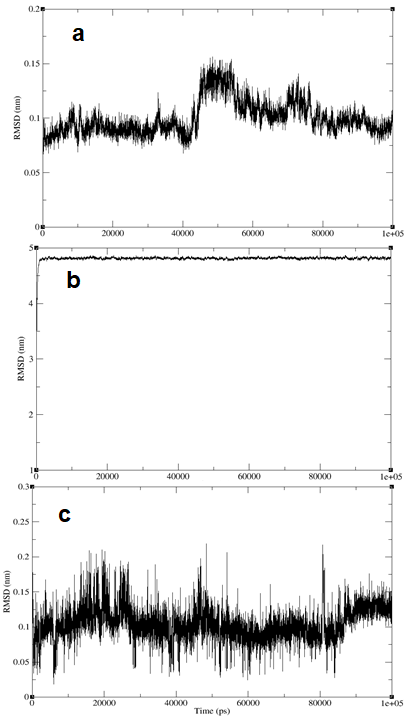


**Fig. S17.** RMSD evolution for (a) the protein (1X2J), (b) ligand (**7**), and protein-ligand complex (black) during 100 ns MD simulation.


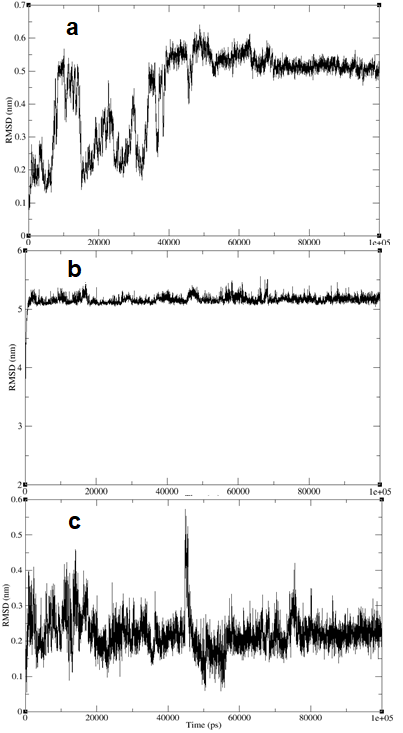


**Fig. S18.** RMSD evolution for (a) the protein (1P60), (b) ligand (**7**), and protein-ligand complex (black) during 100 ns MD simulation.


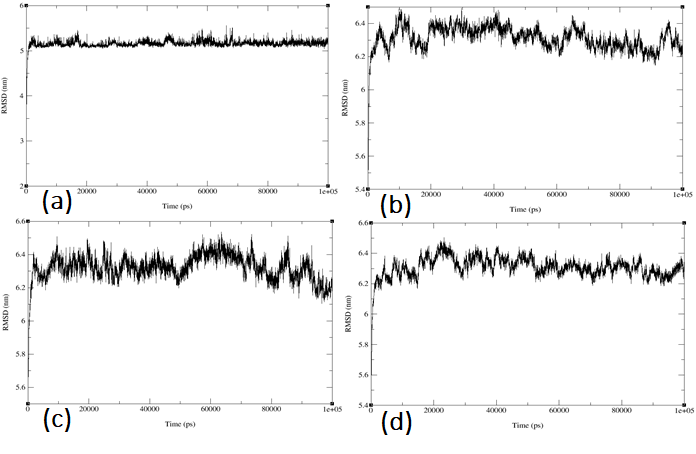


**Fig. S19.** RMSD evolution for (a) ligand (**7**), (b) ligand (**10**), (c) ligand (**14**), and (d) Reference drug (Taxifolin) during 100 ns MD simulation.


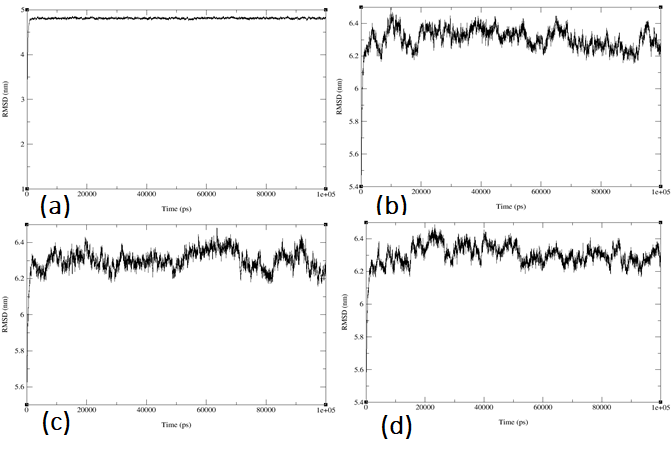


**Fig. S20.** RMSD evolution for the protein (1X2J), (a) ligand (**7**), (b) ligand (**10**), (c) ligand (**14**), and (d) Reference drug (Taxifolin) during 100 ns MD simulation.

**
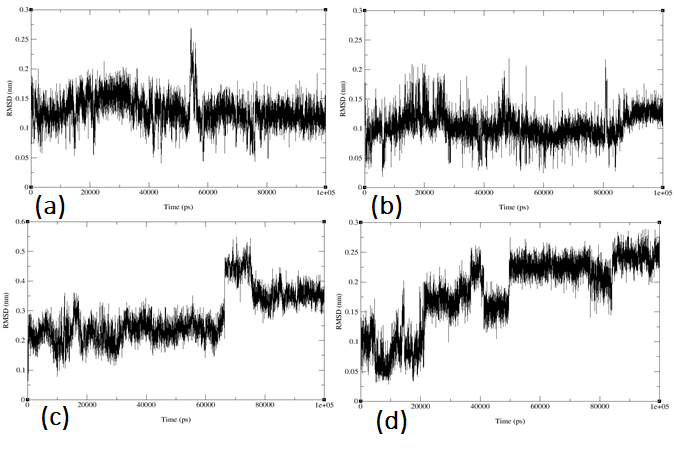
**

**Fig. S21.** RMSD evolution for the protein (1X2J)-ligand complexes (a) ligand (**7**), (b) ligand (**10**), (c) ligand (**14**), and (d) Reference drug (Taxifolin) during 100 ns MD simulation.


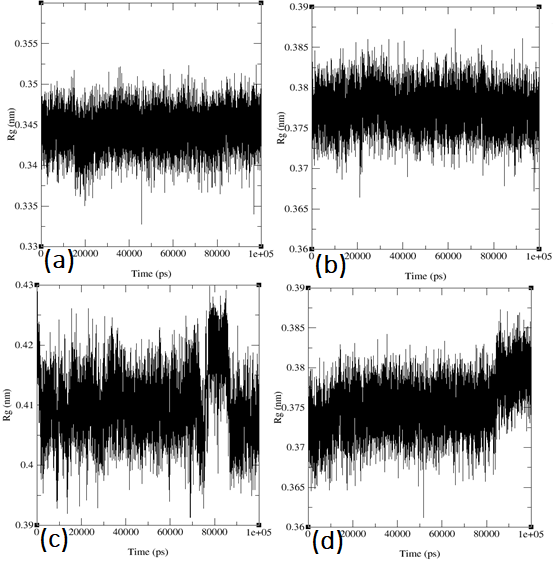


**Fig. 22.** MD simulation evolution of Rg for graphs (a) ligand (**7**), (b) ligand (**10**), (c) ligand (**14**), and (d) Reference drug (Taxifolin) during 100 ns MD simulation


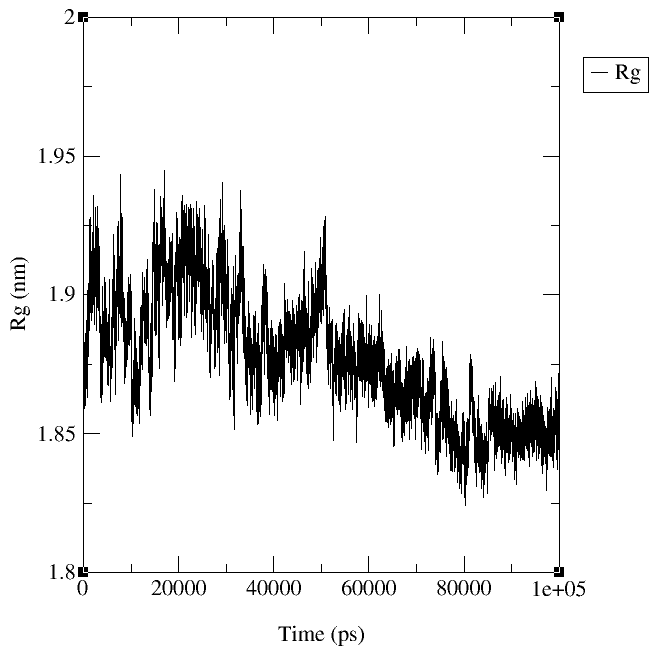

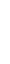

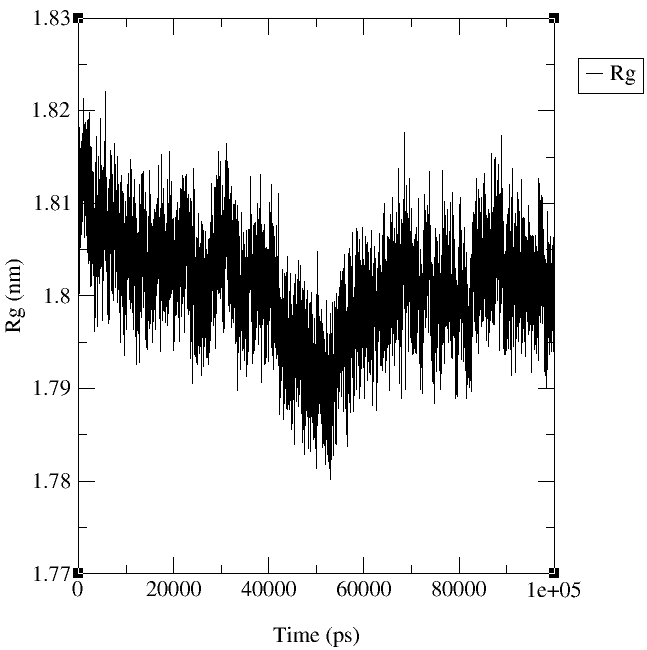

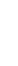

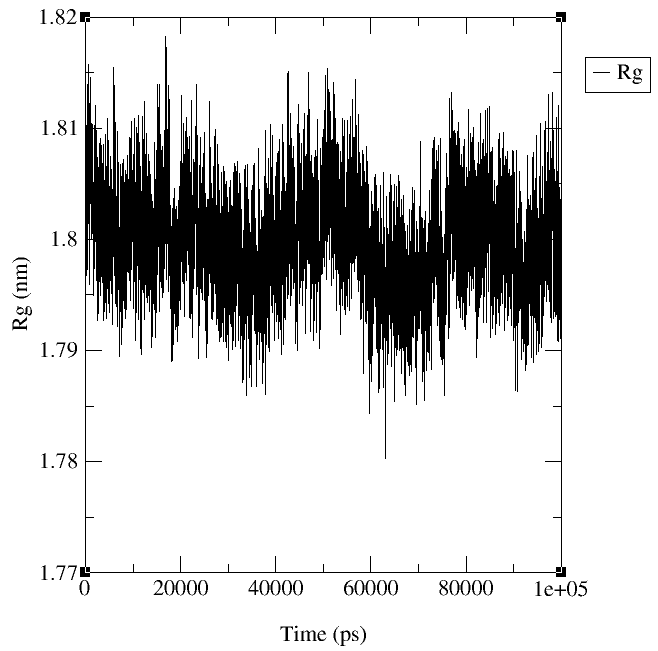

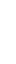

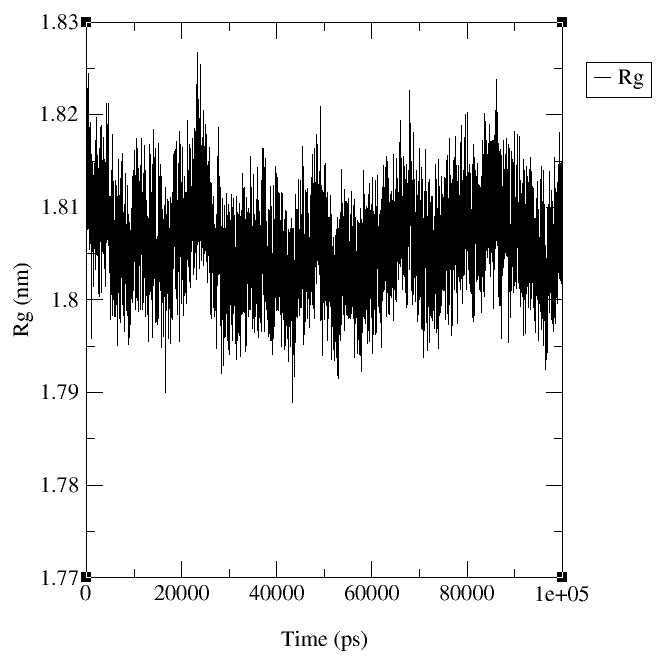

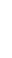


**(a)**

**(b)**

**(c)**

**(d)**

**Fig. 23.** MD simulation evolution of Rg for the protein (1X2J), (a) ligand (**7**), (b) ligand (**10**), (c) ligand (**14**), and (d) Reference drug (Taxifolin) during 100 ns MD simulation

**
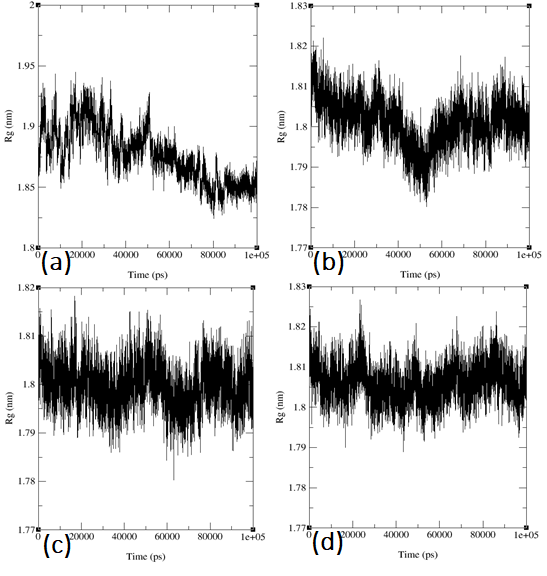
**

**Fig. S24.** Rg evolution for the protein (1X2J)-ligand complexes (a) ligand (**7**), (b) ligand (**10**), (c) ligand (**14**), and (d) Reference drug (Taxifolin) during 100 ns MD simulation.

**
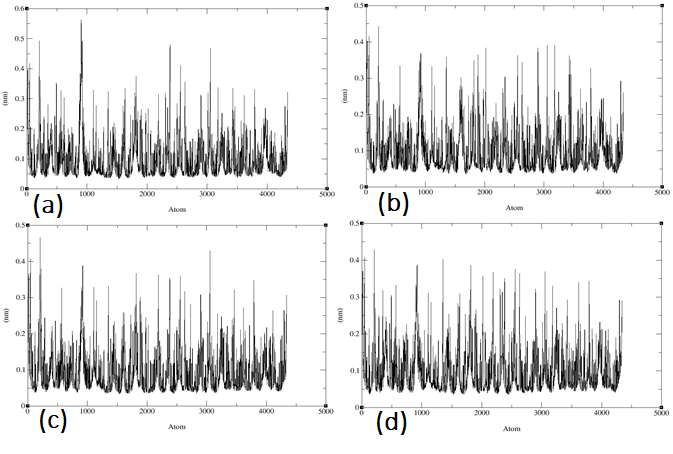
**

**Fig. S25.** RMSF evolution for the protein (1X2J)-ligand complexes (a) ligand (**7**), (b) ligand (**10**), (c) ligand (**14**), and (d) Reference drug (Taxifolin) during 100 ns MD simulation.
